# Tenofovir Alafenamide Versus Tenofovir Disoproxil Fumarate for Preventing Vertical Transmission in Chronic Hepatitis B Mothers: A Systematic Review and Meta-Analysis

**DOI:** 10.1093/cid/ciae288

**Published:** 2024-05-28

**Authors:** Calvin Q Pan, Lin Zhu, Andy S Yu, Yuchan Zhao, Bo Zhu, Erhei Dai

**Affiliations:** Guangzhou Medical Research Institute of Infectious Diseases, Center for Liver Diseases, Guangzhou Eighth People's Hospital, Guangzhou Medical University, Guangzhou, Guangdong, China; Division of Gastroenterology and Hepatology, Department of Medicine, NYU Langone Health, New York University Grossman School of Medicine, New York, New York, USA; Department of Infectious Disease and Clinical Microbiology, Beijing Chao-Yang Hospital, Capital Medical University, Beijing, China; Pacific Gastroenterology and Endoscopy, San Jose, California, USA; Hebei Key Laboratory of Immune Mechanism of Major Infectious Diseases and New Technology of Diagnosis and Treatment, The Fifth Hospital of Shijiazhuang, Hebei Medical University, Shijiazhuang, China; Quality Control Department, The First Affiliated Hospital of Shandong First Medical University, Jinan, China; Hebei Key Laboratory of Immune Mechanism of Major Infectious Diseases and New Technology of Diagnosis and Treatment, The Fifth Hospital of Shijiazhuang, Hebei Medical University, Shijiazhuang, China

**Keywords:** hepatitis B virus, pregnancy, mother-to-child transmission, TDF, TAF

## Abstract

**Objective:**

International guidelines recommend maternal tenofovir disoproxil fumarate (TDF) therapy accompanied by infant immunoprophylaxis to prevent hepatitis B virus (HBV) mother-to-child transmission (MTCT) in highly viremic mothers. However, pooled analyses for tenofovir alafenamide (TAF) effects and comparisons between the 2 regimens are lacking.

**Design:**

In this meta-analysis, pairs of independent reviewers performed multiple database searches from inception to 31 March 2024 and extracted data from cohort studies and randomized controlled trials (RCTs) in highly viremic mothers. The outcomes of interest were the reduction of MTCT and safety in the TDF-treated, TAF-treated, and control groups.

**Results:**

We included 31 studies with 2588 highly viremic mothers receiving TDF, 280 receiving TAF, and 1600 receiving no treatment. Compared to the control, TDF therapy reduced the MTCT rate in infants aged 6–12 months (risk ratio: 0.10, 95% confidence interval [CI] .07–.16). Pairwise meta-analysis between TAF and TDF revealed similar effects on reducing MTCT (risk ratio: 1.09, 95% confidence interval .16–7.61). Network meta-analysis showed equal efficacy of the 2 regimens in reducing MTCT (risk ratio: 1.09, 95% CI .15–7.65). The surface under the cumulative ranking curve revealed TDF as the best regimen compared with TAF (probability ranking: .77 vs .72), while receiving a placebo during pregnancy had the lowest efficacy (probability ranking 0.01). There were no safety concerns for mothers and infants in all regimens.

**Conclusions:**

Compared to placebo or no treatment, maternal TDF and TAF prophylaxis are equally effective and without safety concerns in reducing MTCT in highly viremic mothers.

## Teaser

The study conducted pair-wise and network meta-analyses to compare the efficacy and safety of maternal TAF versus TDF in preventing HBV vertical transmission among highly viremic mothers. The findings indicated that both regimens were equally effective without safety concerns.

Hepatitis B virus (HBV) infection stands as a significant public health threat, responsible for approximately 820 000 deaths attributed to complications such as cirrhosis and liver cancer [1, 2]. Chronic HBV infection (CHB) predominantly arises from early-life exposure to HBV [3, 4], notably through mother-to-child transmission (MTCT). In striving for the World Health Organization's (WHO) objective of eradicating global HBV infection by 2030, preventing MTCT in pregnant women with CHB emerges as a pivotal measure to curtail new instances of chronic HBV infection [3].

Presently, international guidelines advocate for the administration of a series of HBV vaccines to all infants born to CHB mothers within their first year of life [5, 6]. Furthermore, infants born to HBeAg-positive mothers are recommended to receive a birth dose of HBV immunoglobulin (HBIg) alongside the HBV vaccine [3, 4, 7]. Given that maternal HBV DNA levels exceeding 200 000 IU/mL at delivery heighten the risk of immunoprophylaxis failure in infants [8–11], these mothers are advised to undergo tenofovir disoproxil fumarate (TDF) therapy from gestational weeks 24–32 until delivery to mitigate MTCT. Alternatively, second-line (non-preferred) therapies such as telbivudine or lamivudine may be considered, although they carry the risk of antiviral resistance and maternal viremia rebound [5, 6, 12–14].

Although entecavir, TDF, and tenofovir alafenamide (TAF) represent first-line antiviral treatments for CHB [5, 6], the utilization of TAF therapy in pregnant mothers lacks endorsement in international guidelines [3]. A recent review and meta-analysis by Funk and colleagues encompassing data predating 2020 evaluated the efficacy and safety of TDF, lamivudine, and telbivudine prophylaxis for MTCT prevention [15] yet omitted considerations regarding maternal TAF therapy for MTCT prevention. Furthermore, the study did not provide insights into the long-term safety outcomes following fetal exposure to TDF.

Recently, numerous cohort studies and a randomized trial investigating maternal TAF prophylaxis for MTCT prevention have been published, accompanied by additional evidence from long-term follow-up studies on fetal exposure to maternal TDF therapy [9, 16, 17]. Consequently, we conducted both paired-wise and network data analyses to compare the efficacy and safety of prepartum antiviral prophylaxis with TAF therapy, TDF therapy, and placebo (or non-treatment) in preventing MTCT. Additionally, we synthesized the newly available long-term safety data concerning fetal exposure to maternal TDF therapy [9, 16, 17]. We contend that our review furnishes crucial insights to aid clinicians in managing CHB mothers. Importantly, the findings from our current meta-analysis may inform updates to international guidelines, potentially including maternal TAF prophylaxis as another first-line option for highly viremic mothers.

## METHODS

### Eligibility Criteria and Search Strategy

In adherence to a pre-registered protocol in PROSPERO (CRD 42021258449), this review was conducted and results were reported following PRISMA guidelines [18]. We included cohort studies or randomized controlled trials (RCTs) published in full that met the following criteria: (1) enrollment of CHB pregnant mothers with HBV DNA ≥200 000 IU/mL; (2) administration of appropriate immunoprophylaxis to infants; (3) utilization of TAF or TDF during pregnancy in one study arm for MTCT prevention; and (4) reporting of clinical outcomes with aggregate data, including MTCT rate indicated by infant HBsAg positivity and/or detectable HBV DNA after 6 months, along with maternal/infant safety data. Exclusion criteria comprised: (1) animal or translational studies; (2) maternal coinfection with hepatitis A, C, D, E virus, or human immunodeficiency virus (HIV); (3) study treatment arm with <10 patients or providing only second-line antiviral therapy such as telbivudine, adefovir, or lamivudine; and (4) cohort studies (non-RCTs) with a Newcastle Ottawa scale score <5 indicating high risk of bias.

A literature search was conducted across 3 English-language databases (PubMed, Embase, and Cochrane) and 2 Chinese-language databases (CNKI and Wanfang) from inception until 31 March 2024. Search strategies employed keywords encompassing “HBV,” “pregnancy,” “antiviral treatment,” and “MTCT” (Supplementary Appendix 1) to identify relevant articles.

### Study Selection and Data Extraction

Three investigators independently screened titles, keywords, and abstracts in published articles across both English-language (L. Z., B. Z., and A. S. Y.) and Chinese-language databases (L. Z., B. Z., and Y. Z.). Eligible studies were identified, and full-text papers were reviewed individually by each investigator. Discrepancies in study selection were resolved through consensus or discussions with corresponding authors as third reviewers (C. Q. P. and E. D.), who arbitrated any disagreements.

Relevant data were extracted in duplicate from each eligible study by 2 groups of investigators using a standardized form piloted by the study team. Attempts were made to clarify duplicated study populations with corresponding authors, particularly when assessing studies from the same sites with overlapping enrollment criteria, recruitment periods, and intervention types. Only the most recent publication was included if multiple articles reported the same study population, unless a different publication exhibited lower risk of bias.

### Outcomes and Risk of Bias Assessment

Outcomes of interest included: (1) reduction of MTCT rates and safety in TDF-treated, TAF-treated, and control groups; (2) effects of TDF or TAF on MTCT rates when initiated during the second versus third trimester; (3) effects of birth-dose immunoprophylaxis timing on MTCT rates; (4) infant safety outcomes including fetal death, prematurity rates, congenital malformations, and adverse events; (5) assessment of infant physical development at birth and beyond 6 months; (6) maternal outcomes, including changes in alanine aminotransferase (ALT) levels; and (7) maternal adverse events and obstetric complications.

Two investigators (L. Z. and B. Z.) independently assessed the risk of bias to evaluate systematic error [19]. Quality of the evidence, including certainty in estimates derived from network meta-analysis, was evaluated using the Grading of Recommendations Assessment, Development, and Evaluation (GRADE) approach [20], with ratings classified into high, moderate, low, and very low levels. Discrepancies were resolved through group consensus.

### Data Analysis and Statistical Methods

When conducting a pairwise meta-analysis for dichotomous outcomes between the 2 regimens [21], we employed a random-effects model to estimate pooled relative risk (RR) and 95% confidence intervals (CIs) for the differences. This analysis was based on data derived from per-protocol analyses in individual full-text papers, utilizing binomial distributions. For continuous outcomes, we calculated the weighted mean difference between baseline values and those at the longest follow-up duration for each study, estimating pooled effects. Directed meta-analysis was conducted using software including STATA (version 17.0) and R Studio (version 1.3.1093). Statistical heterogeneity was assessed using Cochran Q statistic and I^2^ statistic, where a *P* value <.1 and an I^2^ value ≥50% indicate high heterogeneity. If the I^2^ value is <50%, heterogeneity among studies is deemed acceptable. To include all relevant data regardless of the chosen effect measure, trials with zero events were assessed, utilizing the continuity correction (adding 0.5) method, as sample sizes of 2-arm studies were well balanced. Publication bias was evaluated through the examination of funnel plots and Egger's regression asymmetry test [22].

## RESULTS

**Figure 1.**
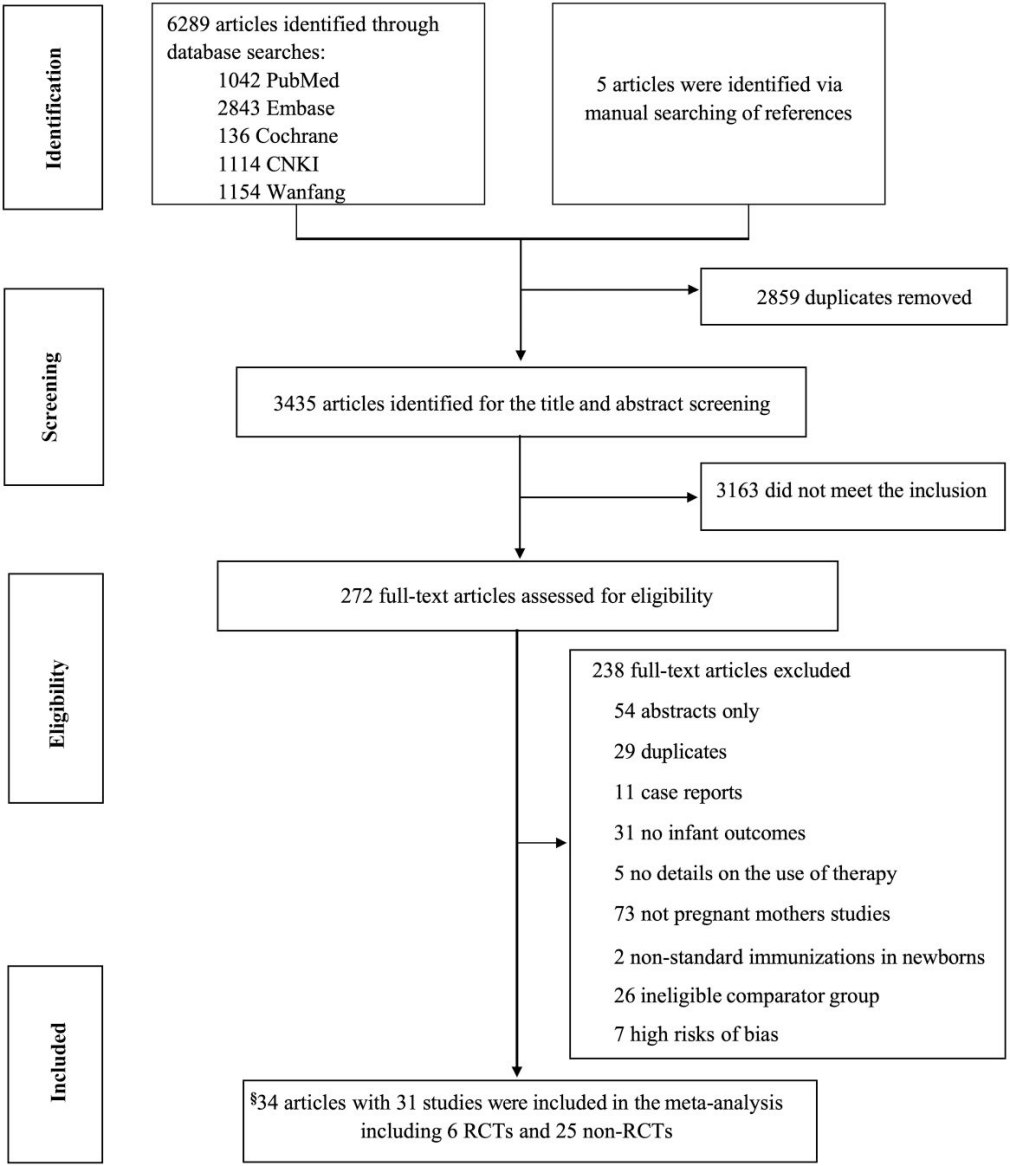
Study selection process. This figure depicts the data selection process for systematic review and meta-analysis through the search of multiple databases. A total of 6289 citations were identified across 5 databases. Following the application of inclusion and exclusion criteria, 31 studies were ultimately selected and included in the meta-analysis. § Three studies published both interim and long-term outcome reports (original articles) on the same cohorts. Abbreviations: non-RCTs, non-randomized controlled trials; RCTs, randomized controlled trials.

A total of 6289 citations were identified across 5 databases, comprising 1042 from PubMed, 2843 from Embase, 136 from Cochrane, 1114 from CNKI, and 1154 from Wanfang. Additionally, 5 citations were manually identified through reference searches. Among the 272 citations assessed in full text, 31 studies were ultimately included in the analysis (Figure 1). The average weighted kappa for study selection was 0.91 (95% CI: .83–.98).

### Characteristics of Included Studies

Of the 31 eligible studies, 6 were RCTs and 25 were non-RCTs, with a total enrollment of 4468 CHB pregnant women (2588 TDF-treated, 280 TAF-treated, and 1600 untreated). Twenty-seven studies (87%) were conducted in China [10, 23–48] with one study each in Canada [49], Thailand [50], Australia [51], or Turkey [52]. All studies excluded mothers coinfected with HCV, HIV, or HDV. Maternal intervention commenced either in the second or third trimester until delivery or postpartum weeks 4–12. Thirty studies (96.77%) reported timely immunoprophylaxis with HBV vaccine, with HBIG administered to all infants. Further characteristics of the included studies are summarized in Table 1.

**Table 1.** Characteristics of the Studies Included in the Current Meta-analysis

Author (Year)	Country	Study Design (RCTs/non-RCTs) [Period-years]	Mean/median Age (Years), Treatment vs Control	Pregnant Women on Treatment vs Control (N)	Infants Assessed in the Treatment/ Control Groups (N)	Inclusion Criteria for HBeAg/HBV-DNA (log10 IU/mL)	Time of Therapy (Gestational Weeks/Weeks After Delivery)	Baseline Mean/Median HBV-DNA (log10 IU/mL) in Treatment vs Control Groups	Baseline Mean/Median ALT (U/L) in Treatment vs Control Groups	Birth Dose of HBIG (Dose, Timing)	Birth Dose of the Vaccine (Dose, Timing)	Subsequent Vaccine (Months)	MTCT Rates, % (N) in Treatment vs Control Groups
Maternal TDF therapy (300 mg orally once a day) vs Placebo													
Chen H et al (2015)^a^	China (Taiwan)	Non-RCTs [2011–13]	32.5 ± 3.2 vs 32.4 ± 3.1	62/56	65/56	Positive/ ≥ 7.5	30–32/4 (postpartum)	8.25 ± 0.45 vs 8.24 ± 0.35	23.27 ± 36.2 vs 16.59 ± 14.43	100 IU, < 24 hr	5 ug, < 2 4 hr	1, 6	1.5% (1/65) vs 10.7% (6/56)
Pan CQ et al (2016)^a^	China	RCTs [2012–13]	27.4 ± 3.0 vs 26.8 ± 3.0	97/100	95/88	Positive/ ≥ 5.0	30–32/4 (postpartum)	8.2 ± 0.5 vs 8.0 ± 0.7	23.0 ± 22.4 vs 20.5 ± 15.4	200 IU, < 12 hr	10 ug, < 12 hr	1, 6	0% (0/95) vs 6.8% (6/88)
Samadi K et al (2016)	Canada	Non-RCTs [2011–14]	30 (28, 34) vs 32	23/138	24/146	Both/ > 7.7	28–32/12 (postpartum)	7.7 (3.2–8.1) vs 2.3 (1.6–3.1)	30 (18–50) vs 17 (12–24)	NR, at birth	NR, at birth	2, 6	0% (0/24) vs 1.4% (2/146)
Liu M et al (2017)	China	RCTs [2014–16]	30 (22, 38) vs 29	20/20	20/20	positive/ ≥ 5.0	28–30/At delivery	6.51 ± 0.91 vs 6.47 ± 1.00	38 (11–154) vs 46 (8–126)	200 IU, < 24 hr	10 ug, < 24 h	1, 6	5% (1/20) vs 30% (6/20)
Chen W et al (2017)	China	Non-RCTs [2014–15]	28.7 ± 5.7 vs 29.9 ± 5.1	30/44	30/44	positive/ ≥ 6.0	28/At delivery	7.5 ± 0.5 vs 7.5 ± 0.55	70.40 ± 15.44 vs 68.98 ± 16.35	100 IU, at birth	10 ug, at birth	1, 6	0% (0/30) vs 25% (11/44)
Jourdain G et al (2018)^a^	Thailand	RCTs [2013–15]	25.5 (22.6, 29.1) vs 26.7	152/154	147/147	positive/NR	28/8 (postpartum)	7.6 ± 1.5 vs 7.3 ± 1.7	NR	200 IU, < 3 hr	10 ug, < 3 hr	1, 2, 4, 6	0% (0/147) vs 2.0% (3/147)
Lin Y et al (2018)	China	RCTs [2013–16]	28.3 ± 3.6 vs 28.1 ± 3.4	59/52	58/52	positive/ ≥ 6.0	24/4 (postpartum)	7.44 ± 8.0 vs 7.66 ± 0.55	54.62 ± 105.7 vs 57.5 ± 103.3	NR, < 24 hr	NR, < 12 hr	1, 6	0% (0/58) vs 13.5% (7/52)
Greenup AJ et al (2014)	Australia	Non-RCTs [2007–10]	NR	58/20	58/20	Both/ > 7.0	32/12 (postpartum)	7.8 vs NR	28 (22–36) vs 25 (17–31)	NR, at birth	NR, at birth	2, 4, 6	2.3% (1/44) vs 20% (2/10)
Celen MK et al (2013)	Turkey	Non-RCTs [2010–12]	28.2 ± 4.1 vs 26.9 ± 2.9	21/24	21/23	positive/ ≥ 7.0	18–27/4 (postpartum)	8.28 vs 8.31	56 (22–71) vs 52 (19–77)	200 IU, < 24 hr	20 ug, < 24 hr	4 824	0% (0/21) vs 8.7% (2/23)
Wang YC et al (2020)	China	Non-RCTs [2014–17]	25.6 ± 7.1 vs 24.95 ± 6.1	72/56	72/56	positive/ ≥ 6.0	28/NR	7.32 ± 5.78 vs 7.24 ± 4.96	NR	NR, < 24 hr	NR, < 72 hr	1, 6	0% (0/72) vs 17.9% (10/56)
Chen CY et al (2019)	China	Non-RCTs [2014–16]	25.9 ± 3.09 vs 25.38 ± 2.9	80/84	80/84	positive/ ≥ 6.0	<12/NR	7.63 ± 0.39 vs 7.55 ± 0.38	327.85 ± 84.42 vs 310.49 ± 55.19	NR, at birth	NR, at birth	NR	0% (0/80) vs 8.3% (7/84)
Shen GJ et al (2021)	China	Non-RCTs [2015–16]	25.4 ± 3.4 vs 25.1 ± 3.0	40/31	40/31	NR/ ≥ 6.0	26–32/At delivery	7.34 ± 0.65 vs 7.21 ± 0.76	21.70 ± 5.40 vs 20.50 ± 4.4	100 IU, < 12 hr	10 ug, < 12 hr	1, 6	0% (0/40) vs 9.7% (3/31)
Zhang JM et al (2021)	China	Non-RCTs [2018–19]	26.97 ± 4.8 vs 26.70 ± 4.81	39/37	39/37	NR/ ≥ 6.0	24/At delivery	7.71 ± 0.77 vs 7.67 ± 0.69	24.64 ± 11.12 vs 23.27 ± 9.32	100 IU, < 12 hr	10 ug, < 12 hr	1, 6	0% (0/39) vs 10.8% (4/37)
Liu JF et al (2019)	China	Non-RCTs [2010–16]	28.4 ± 4.4 vs 27.1 ± 4.7	325/136	325/136	NR/ ≥ 6.0	22–28/12 (postpartum)	7.68 ± 0.70 vs 7.71 ± 0.79	53.34 ± 71.87 vs 41.16 ± 62.46	200 IU, < 24 hr	10 ug, < 24 hr	1, 6	2.5% (8/325) vs 22.1% (30/136)
Zeng J et al (2019)	China	Non-RCTs [2013–17]	26.5 ± 9.5 vs 25.7 ± 10.9	51/36	51/36	positive/ ≥ 7.0	22–28/NR	7.9 ± 0.8 vs 7.7 ± 0.5	143.30 ± 104.60 vs 132.30 ± 78.30	200 IU, < 6 hr	20 ug, < 12 hr	1, 6	0% (0/51) vs 11.1% (4/36)
Chang K et al (2019)	China (Taiwan)	Non-RCTs [2011–16]	32.8 ± 3.6 vs 22	110/91	115/93	positive/ ≥ 7.5	30–32/4 (postpartum)	8.25 ± 0.48 vs 8.29 ± 0.40	20.88 ± 28.94 vs 19.10 ± 23.85	100 IU, < 24 hr	NR, < 24 hr	1, 6	17.4% (2/115) vs 10.8% (10/93)
Wang Y et al (2019)	China	Non-RCTs [2014–16]	29.5 ± 3.8 vs 28.7 ± 4.2	128/72	128/72	positive/ ≥ 6.0	28/4 (postpartum)	7.87 ± 0.51 vs 7.83 ± 0.65	16.50 (12.00, 23.00) vs 14.00 (10.00, 18.00)	100 IU, < 6 hr	20 ug, < 6 hr	1, 6	0% (0/128) vs 5.6% (4/72)
Mao C et al (2019)	China	Non-RCTs [2016–19]	26.6 ± 3.5 vs 25.7 ± 3.9	156/102	156/102	NR/ ≥ 6.0	24/At delivery	5.68 ± 2.54 vs 5.45 ± 2.67	23.41 ± 4.63 vs 22.79 ± 4.61	200 IU, < 12 hr	10 ug, < 12 hr	1, 6	1.28% (2/156) vs 8.8% (9/102)
Ma L et al (2019)	China	Non-RCTs [2015–17]	25.4 ± 3.7 vs 26.1 ± 2.6	56/27	56/27	NR/ > 7.0	28/At delivery	7.90 ± 1.0 vs 7.8 ± 0.8	NR	100 IU, NR	10 ug, NR	1, 6	0% (0/56) vs 18.5% (5/27)
Gao X et al (2020)	China	Non-RCTs [2010–18]	29.6 ± 2.8 vs 29.1 ± 3.4	81/63	81/63	Both/ ≥ 5.0	NR	6.3 ± 1.0 vs 6.4 ± 1.2	209.60 ± 140.30 vs 187.90 ± 118.70	200 IU, < 2 hr	10 ug, < 2 hr	1, 6	0% (0/81) vs 6.3% (4/63)
Ye Z et al (2021)	China	Non-RCTs [2016–20]	28.3 ± 2.1 vs 29.0 ± 1.9	26/26	26/26	NR/ ≥ 6.0	24–28/At delivery	7.74 ± 0.52 vs 7.5 ± 0.5	NR	100 IU, < 24 hr	10 ug, < 24 hr	1, 6	0% (0/26) vs 15.4% (4/26)
Kuang C et al (2021)	China	Non-RCTs [2017–19]	25.3 ± 1.6 vs 25.2 ± 1.6	80/83	80/83	positive/ ≥ 6.3	18/At delivery	7.54 ± 0.37 vs 7.47 ± 0.29	327. 12 ± 83. 22 vs 327. 33 ± 83. 31	NR	NR	NR	0% (0/80) vs 8.4% (7/83)
Cui D et al (2021)	China	Non-RCTs [2017–19]	30.1 ± 4.4 vs 30.2 ± 4.4	54/54	54/54	NR/ ≥ 5.3	28/At delivery	7.57 ± 1.23 vs 7.59 ± 1.21	21.70 ± 5.40 vs 20.50 ± 4.40	100 IU, < 24 hr	10 ug, < 24 hr	1, 6	0% (0/54) vs 13.0% (7/54)
Ran R et al (2021)^b^	China	Non-RCTs [2016–18]	29 (27, 32)	253/NR	253/NR	NR/ ≥ 6.0	23–26/4 (postpartum)	8.21 (8.04, 8.23) vs 8.10 (7.77, 8.23) no placebo	4.41 (3.42, 4.89) vs 4.53 (3.29, 5.40) no placebo	200 IU, < 2 hr	10 ug, < 2 hr	1, 6	0% (0/253)
			30 (28, 33)				27–34/4 (postpartum)						
Hu MF et al (2018)^b^	China	Non-RCTs [2016–18]	28.4 ± 1.4	90/30	90/30	NR/ ≥ 6.0	Pre-pregnancy	7. 44 ± 0. 39	5. 84 ± 1. 35	200 IU, < 2 hr	10 ug, < 2 hr	1, 6	0% (0/90) vs 10% (3/30)
			23.2 ± 3.3				14	7. 50 ± 0. 47	5. 55 ± 1. 19				
			24.4 ± 3.1				28	7. 38 ± 0. 66	5. 67 ± 1. 25				
Wang HB et al (2018)^b^	China	Non-RCTs [2013–16]	NR	100/20	100/20	NR	20	7.0	33.00 ± 10.84	200 IU, < 2 hr	10 ug, < 2 hr	1, 6	0% (0/100) vs 10% (2/20)
							24	7.1	35.65 ± 20.82				
							28	7.2	27.05 ± 17.42				
							32	7.2	32.55 ± 15.44				
							36	6.7	37.10 ± 17.19				
Huang XL et al (2023)	China	RCTs [2020–21]	28.46 ± 3.18 vs 28.15 ± 3.29	44/44	44/44	positive/ ≥ 5.3	24/At delivery	6.95 ± 0.96 vs 6.89 ± 0.85	NR	100 IU, < 12 hr	10 ug, < 12 hr	1, 6	0% (0/44) vs 15.9% (7/44)
Maternal TAF therapy (25 mg orally once a day) vs TDF therapy (300 mg orally once a day)													
Li B et al (2021)	China	RCTs [2019]	27.5 ± 4.1 vs 26.9 ± 3.9	36/36	36/36	positive/ ≥ 6.0	24/At delivery	7.95 ± 0.4 vs 7.85 ± 0.41	36.37 ± 14.21 vs 38.39 ± 17.75	100 IU, < 12 hr	10 ug, < 12 hr	1, 6	0% (0/36) vs 0% (0/36)
Zeng Q et al (2021)	China	Non-RCTs [2019]	29.6 ± 4.5 vs 29.3 ± 4.2	116/116	117/116	Both/ ≥ 5.0	24–35/At delivery	7.8 ± 0.7 vs 7.8 ± 0.7	17.40 ± 7.80 vs 17.20 ± 6.80	NR, < 12 hr	NR, < 12 hr	1, 6	0% (0/117) vs 0% (0/116)
Zeng Q et al (2021)	China	Non-RCTs [2019–20]	28.8 ± 4.5 vs NR	103/104	102/104	Both/ ≥ 5.0	NR	5.1 ± 3.4 vs 4.6 ± 3.4	122.20 ± 97.50 vs 94.60 ± 78.30	100 IU, < 12 hr	10 ug, < 12 hr	1, 6	0% (0/102) vs 0% (0/104)
Pan SF et al (2024)^c^	China	Non-RCTs [2018–21]	30.4 ± 3.9 vs 29.6 ± 3.2	25/35	25/35	positive/ ≥ 5.3	20–23/NR	7.8 ± 0.7 vs 7.6 ± 0.8	18.0 (16.5, 32.0) vs 25.5 (15.3, 33.3)	100 IU, < 24 hr	10 ug, < 24 hr	1, 6	0% (0/25) vs 0% (0/35)

Abbreviations: ALT, alanine aminotransferase; HBIG, Hepatitis B immune globulin; MTCT, Mother-to-child transmission; Non-RCTs, non-randomized controlled trials; NR, not reported; RCTs, randomized controlled trials; TAF, tenofovir alafenamide; TDF, tenofovir disoproxil fumarate.

^a^These 3 studies also included 3 long-term studies: Pan CQ (2022) [9], Wen WH (2020) [16], and Salvadori N (2019) [17].

^b^3 studies have compared the therapeutic effects of TDF with different initiate times.

^c^This study compare the efficacy and safety of telbivudine (LdT), tenofoviralafenamide fumarate (TAF), and tenofovir disoproxil fumarate (TDF).

To analyze the pooled efficacy effect of each intervention, we stratified patients from the 31 studies into 3 subgroups: mothers receiving TDF prophylaxis, mothers receiving TAF, and those receiving placebo or no antepartum antiviral therapy. Detailed descriptions of the risk of bias assessment are presented in Supplementary Tables 1 and 2.

### Pair-wise Meta-Analysis of HBV Transmission Rates

**Figure 2.**
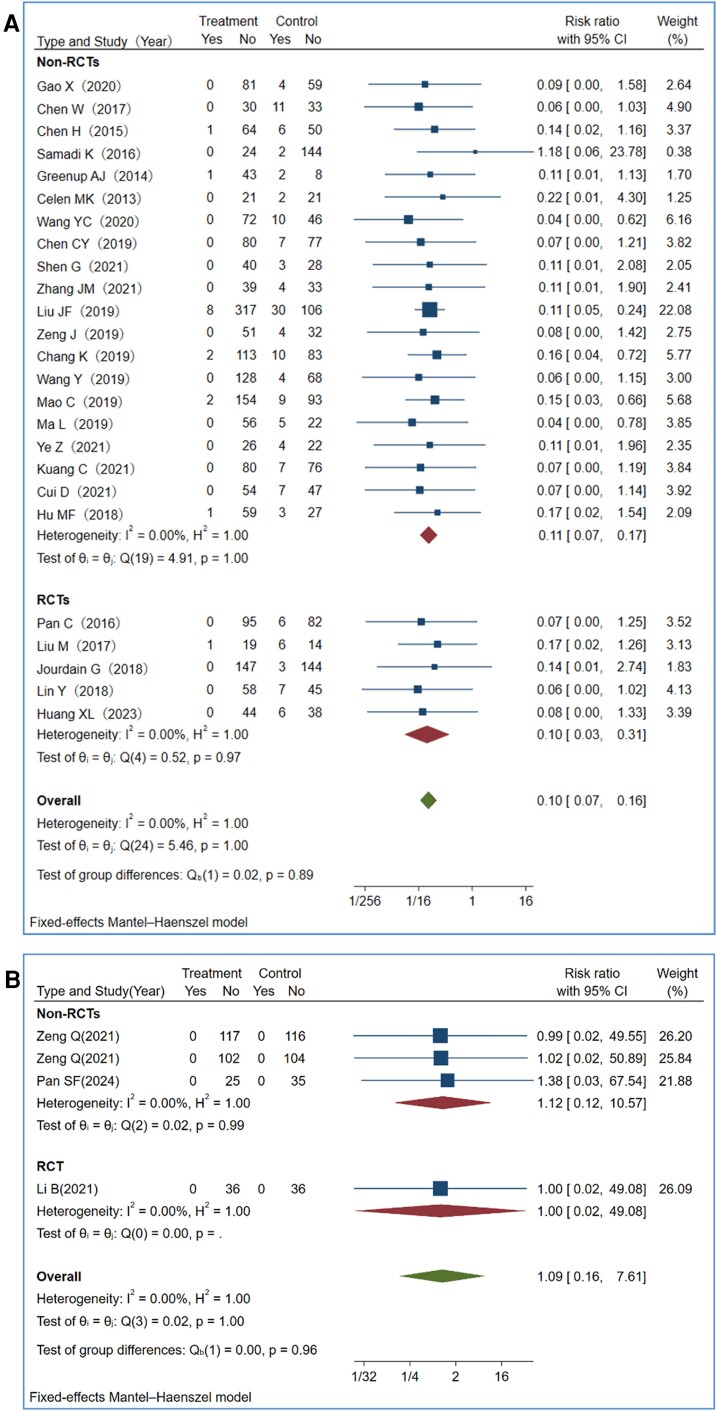
Efficacy of maternal TDF or TAF prophylaxis in preventing MTCT. Assessment of MTCT rates after maternal prophylaxis with TDF or TAF, stratified by study design (RCTs and non-RCTs). *A*, Efficacy of TDF by study design. *B*, Efficacy of TAF by study design. Abbreviations: CI, confidence interval; MTCT, mother-to-child transmission; Non-RCTs, non-randomized controlled trials; RCT, randomized controlled trial; TAF, tenofovir alafenamide; TDF, tenofovir disoproxil fumarate; Yes/No, events numbers/no events numbers.

Maternal TDF therapy, compared to placebo/no treatment, significantly reduced MTCT rates. Pooled RRs for TDF intervention versus control in 27 studies, 5 RCTs, and 22 non-RCTs were 0.10 (95% CI: .07–.16), 0.10 (95% CI: .03–.31), and 0.11 (95% CI: .07–.17), respectively (Figure 2*A*). TDF therapy reduced the likelihood of MTCT, defined by infant HBsAg seropositivity alone (2.24%) or detectable HBV DNA and/or positive HBsAg in infants (0.58%).

Maternal TDF or TAF prophylaxis demonstrated equal effectiveness in reducing MTCT across both RCT and non-RCT studies, with comparable maternal baseline and infant characteristics (Supplementary Tables 3 and 4). When comparing MTCT rates between the 2 regimens (Figure 2*B*), the pooled RR was 1.09 (95% CI: .16–7.61). No statistical heterogeneity (I^2^ = 0/0%) was observed in any meta-analyses utilizing maternal TDF or TAF prophylaxis.

To assess between-study heterogeneity and the summary effect influenced by a specific study, we conducted a sensitivity analysis on all TDF versus control studies by omitting each trial one by one (Supplementary Figure 1), revealing no single study causing heterogeneity or inconsistency. The heterogeneity of TAF versus TDF was not analyzed due to the limited number of available studies.

### Network Meta-Analysis and Efficacy Ranking

Given that published TAF prophylaxis studies were solely compared with TDF prophylaxis in the pairwise meta-analysis [45–47, 53], a network meta-analysis was conducted to assess the efficacy of 3 approaches by comparing MTCT rates in the TDF-treated, TAF-treated, and placebo (or non-treated) patient groups [10, 23–30, 49–53]. Results indicated comparable efficacy between maternal TAF and TDF regimens (*P* value = .68), alongside immunoprophylaxis for infants (risk ratio: 0.10, 95% CI: .07–.16). Further evaluation of the probability of being the best regimen for preventing MTCT was conducted using a probability ranking of the two antiviral regimens and placebo, assessed by the surface under the cumulative ranking curve (SUCRA, Supplementary Appendix 7). The SUCRA comparison suggested that maternal TDF therapy had the highest probability of being the best or most effective regimen for the outcome of interest compared to TAF therapy (probability of .77 vs .72). Receiving a placebo or no treatment exhibited the lowest probability (0.01) of preventing MTCT (Supplementary Figures 2*A* and 2*B*). However, the pairwise analysis suggested that TAF and TDF were equally effective in preventing MTCT of HBV in this special population.

### Subgroup Analyses of MTCT Rates

Efficacy endpoints on MTCT rates did not differ in TDF or TAF intervention according to subgroup analyses stratified by study type (Figure 2), maternal mean HBV DNA levels (6.0–6.9, 7.0–7.9, and 8.0–8.9 logs 10 IU/mL) at baseline, and HBeAg status (Supplementary Figures 3*A* and 3*B*), or publication language (Supplementary Figure 3*C*). In TDF versus control studies, all aforementioned subgroup analyses indicated statistical significance in reducing MTCT rates with TDF prophylaxis compared to placebo.

**Figure 3.**
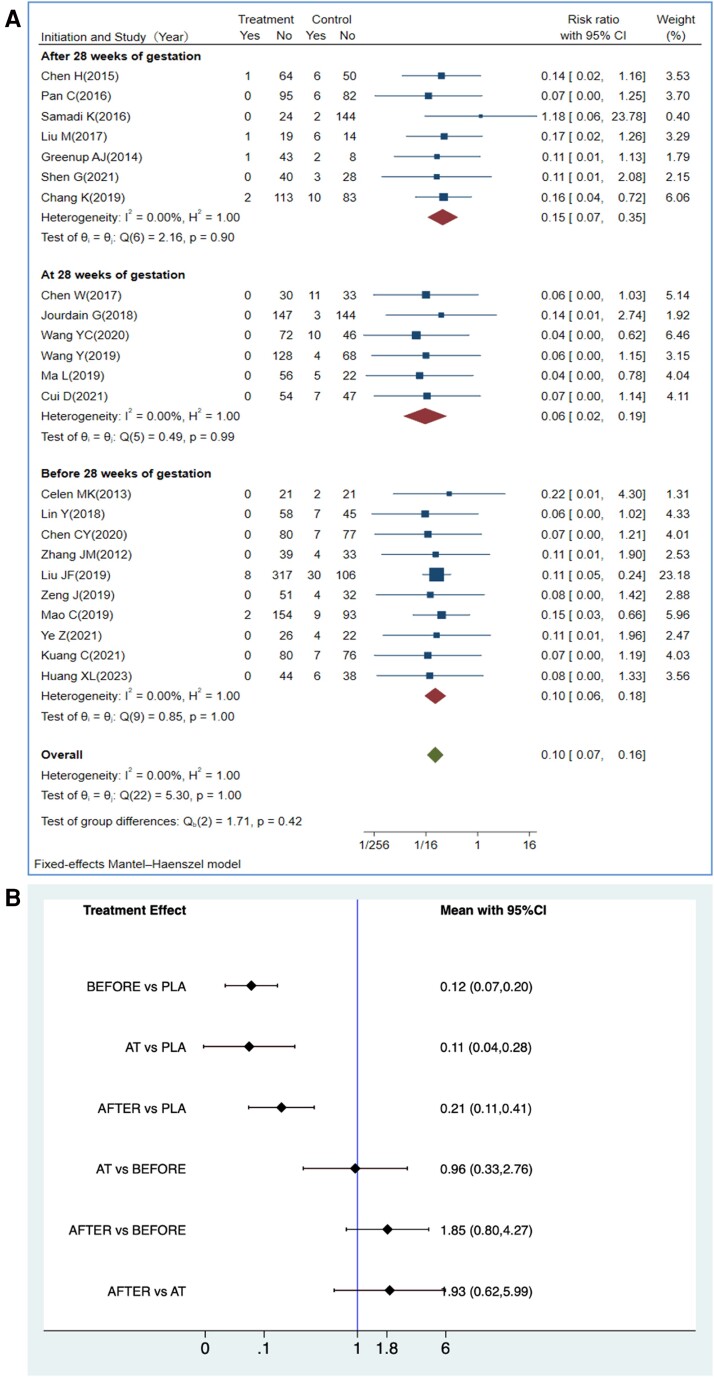
Efficacy of initiating TDF therapy at the second vs the third trimesters. Comparison of MTCT rates when maternal TDF prophylaxis was initiated before, at, or after gestational week 28. TAF data were not included due to all studies initiating maternal prophylaxis at gestational week 28. *A*, Efficacy of TDF therapy by the timing of initiating the therapy. *B*, Head-to-head comparison of earlier vs later initiation of TDF therapy. Abbreviations: AFTER, initiation time of TDF therapy after 28 wks; AT, initiation time of TDF therapy at 28 wks; BEFORE, initiation time of TDF therapy before 28 wks; CI, confidence interval; PLA, placebo; TDF, tenofovir disoproxil fumarate; Yes/No, events numbers/no events numbers.

Regarding the optimal timing for initiating TDF therapy, we compared 3 subgroups: mothers who initiated TDF before gestational week 28, those who started TDF at gestational week 28, and those who received TDF after gestational week 28 (Figure 3). Pooled analyses revealed similar MTCT rates among sub-groups. TAF data were not analyzed as all studies initiated TAF treatment at gestational week 24. Although all birth doses of immunoprophylaxis were administered within 24 hours of birth (Supplementary Figures 3*D* and 3*E*), we stratified them into 3 subgroups based on the timing of HBV vaccine and HBIg administration (within 6 hours, 7–12 hours, and 13–24 hours). Among the 3 subgroups, the baseline maternal HBV DNA levels did not differ, and there was no statistically significant difference in the MTCT rates.

### Infant Safety

**Figure 4.**
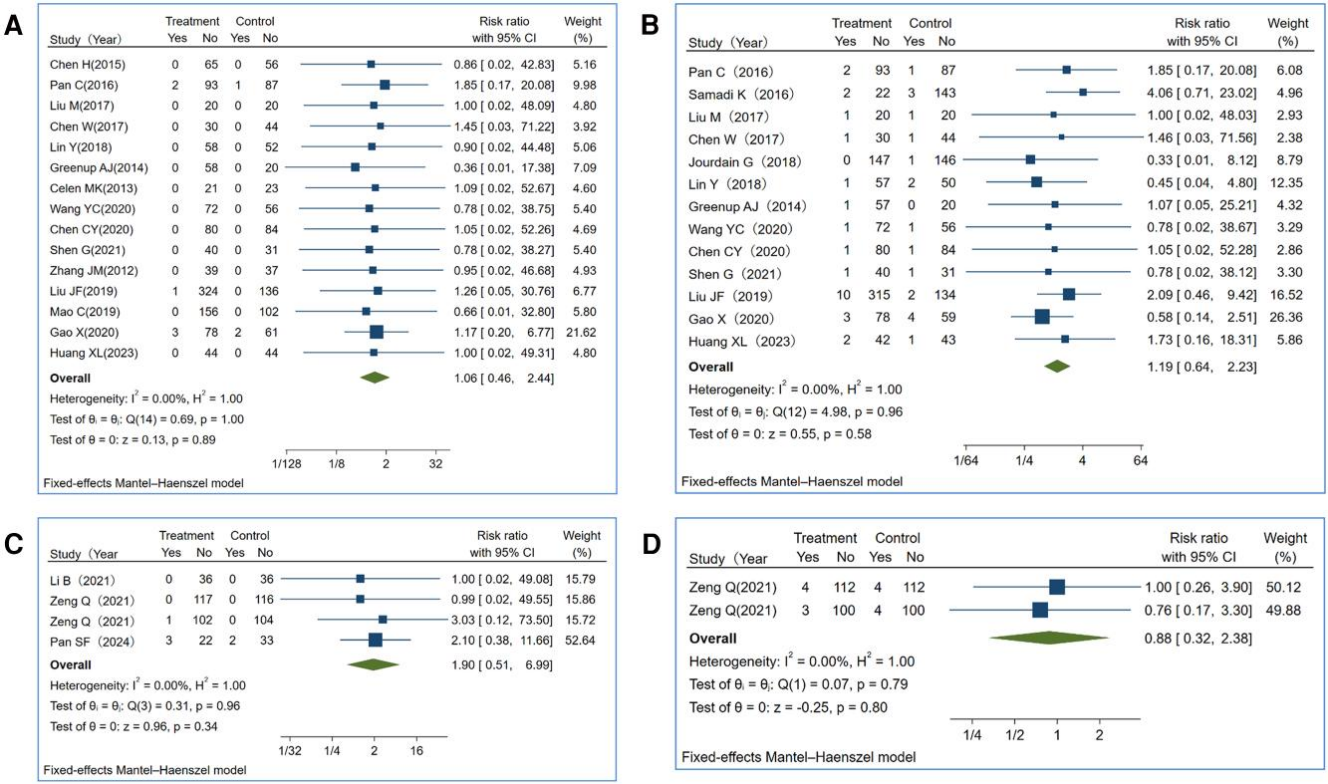
Forest plots of infants' congenital malformation and prematurity rates. Paired comparison of infant negative outcomes between fetal exposure to TDF and placebo (or no treatment) or between fetal exposure to TDF and fetal exposure to TAF. Major outcomes included congenital malformation and prematurity. *A*, Congenital malformations rates for studies comparing TDF therapy vs control. *B*, Prematurity rates for studies comparing TDF therapy vs control. *C*, Congenital malformation rates for studies comparing TAF therapy vs TDF therapy. *D*, Prematurity rates for studies comparing TDF therapy vs TAF therapy. Abbreviations: CI, confidence interval; TAF, tenofovir alafenamide; TDF, tenofovir disoproxil fumarate; Yes/No, events numbers/no events numbers.

In the 15 TDF studies and 4 TAF studies reporting infant outcomes, there was no evidence associating these regimens with negative fetal/infant outcomes (Figure 4). Two RCTs reported a total of 3 fetal deaths in TDF-treated mothers, resulting in a combined RR of 1.11 (95% CI: .5–2.45) [10, 50]. However, no fetal deaths were reported in all TAF studies (Supplementary Figures 4*A* and 4*B*). Regarding prematurity, the pooled RR was 1.19 (95% CI: .64–2.23) when comparing the TDF group with the control group, and 1.90 (95% CI: .51–6.99) when comparing the TAF and TDF groups (Figures 4*B* and 4*D*). In the pooled analysis of the frequency of congenital abnormalities (Figures 4*A* and 4*C*), there was no statistically significant difference when comparing the TDF with the control groups (*P* = .89) or between the TAF-treated and TDF-treated groups (*P* = .34).

Other safety outcomes for infants, including the Apgar score (1 minute), physical growth parameters, and the frequency of grade 3 or 4 adverse events, were comparable among the TDF-treated, TAF-treated, and placebo (non-treated) groups (Supplementary Figures 4*C*–4*G*). Although none of the TAF studies reported bone mineral density scores in infants, the TDF studies providing data revealed similar scores between TDF-exposed infants and controls in pooled analyses (Supplementary Figure 4*H*). Additionally, infants followed up for 2–5 years after fetal TDF exposure showed no statistical significance in physical growth (*P* = .92) and bone mineral density (*P* = .92) compared with the control group (Supplementary Figures 4*I* and 4*J*).

### Maternal Safety

**Figure 5.**
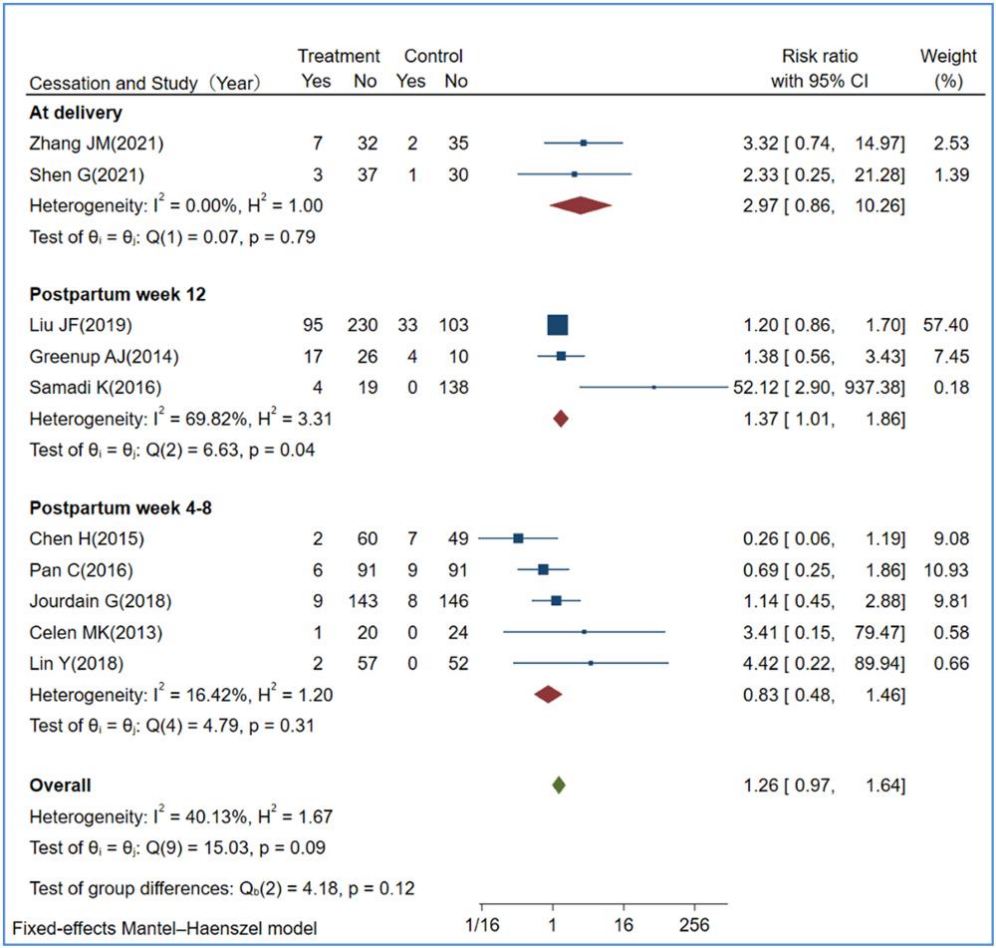
Forest plot of ALT flares by the time of TDF cessation. Only data for TDF vs control were analyzed for postpartum ALT flares, which were stratified by the time of TDF cessation. Because all 4 of the studies on TAF included in the current review had the same design and discontinued antiviral therapy at delivery, the comparison could not be made for the different time points of TAF cessation. Abbreviations: ALT, alanine aminotransferase; CI, confidence interval; TAF, tenofovir alafenamide; TDF, tenofovir disoproxil fumarate; Yes/No, events numbers/no events numbers.

Data for TDF, but not for TAF, versus control were available for assessing ALT flares at different time points of antiviral cessation. ALT flare outcomes did not differ when comparing TDF cessation among delivery, postpartum week 4, week 12, or after week 12 (Figure 5). Pregnancy complications were reported in 18 studies, with 147/943 (15.6%), 378/1493 (25.3%), and 48/244 (19.7%) cases in the control, TDF-treated, and TAF-treated groups, respectively (Supplementary Figures 5*A*–5*B*). Pooled analyses showed comparable frequency not only between TDF and control groups with RR of 1.23 (95 CI%: .78–1.95), but also between TAF-treated and TDF-treated groups with RR of 0.93 (95 CI%: .66–1.31). There was an increased frequency of creatine kinase elevation in the TDF-treated group with an RR of 5.71 (95 CI%: 1.14–28.58; *P* = .03) versus control (Supplementary Figure 5*C*). One case with an elevation of creatine kinase was reported in TAF studies. The frequency of maternal postpartum hemorrhage and severe adverse events (grades III and IV) in mothers who received TDF therapy did not differ from the control. These safety parameters were also comparable when comparing maternal TAF therapy with the TDF regimen (Supplementary Figures 5*D*–5*F*).

### Publication Bias

We performed a risk of bias assessment for HBV MTCT rates as the primary outcome of interest using funnel plots and Egger's test (Supplementary Figures 6*A*–6*D*), which did not indicate small-sample effects in studies. The *P* values of Egger's test for TDF paired with control studies and TDF paired with TAF studies were 0.34 and 0.89, respectively.

## DISCUSSION

Despite appropriate immunoprophylaxis, MTCT rates remain as high as 10% in mothers with HBV DNA levels >200 000 IU/mL [4, 7, 11, 54, 55]. Previous meta-analyses suggested that maternal lamivudine, telbivudine, or TDF prophylaxis could effectively reduce MTCT rates [15, 25, 56–63]. To our knowledge, this study is the first to assess the pooled effects of TAF for preventing MTCT using both pairwise and network data analyses. Our findings suggest maternal TAF prophylaxis as an effective first-line option for these mothers without safety concerns.

Current international guidelines exhibit a discrepancy in recommending when to initiate antiviral treatment during pregnancy (gestational weeks 28–32 vs 24–28) due to inconsistent findings from published studies [3, 5, 6]. Funk's meta-analysis favored initiating antiviral during the second trimester based on data primarily from studies on maternal lamivudine or telbivudine prophylaxis. Our analyses indicate comparable efficacy when TAF or TDF is initiated during the second versus the third trimester. We speculate that the conclusion of early antiviral use from Funk's study may reflect the suboptimal antiviral potency when using second-line therapy. In a viral kinetic study, Pan et al also observed a comparable percentage of child-bearing-age women with high viremia levels achieving target levels of <200 000 IU/mL when treated with TDF for 12 versus 24 weeks (90% [64/71] vs 93% [66/71], *P* = .55) [64].

The major concern of postpartum cessation of antiviral treatment is the risk of postpartum ALT flares. When comparing cessation time points (at delivery, postpartum week 4, week 12, or after week 12), pooled analyses showed that the severity or frequency of ALT flares was not affected by cessation timing of TAF or TDF therapy. Thus, maternal prophylaxis with TDF or TAF should be discontinued at delivery to avoid unnecessary treatment. Further prospective RCTs are needed to provide high-quality evidence to determine this conclusively.

Finally, our study found that TDF or TAF prophylaxis was safe for both mothers and infants. These findings align with a recent antiretroviral pregnancy registry (APR) interim report, involving 2016 and 173 pregnancies with TDF and TAF regimen exposure, respectively [58]. The rates (95% CI) of congenital defects among live births after TDF and TAF exposure during the second/third trimester were 2.7% (2.0%, 3.5%) and 3.5% (1.3%, 7.4%), respectively. These data, along with Funk's pooled analysis, support the safe use of TDF or TAF for mothers during late pregnancy [15, 65]. Additionally, the US guidelines for the use of antiretroviral agents in adults and adolescents with HIV also prefer TDF or TAF as the antiretroviral drugs throughout pregnancy for women with HIV [66]. As maternal TDF treatment was limited to 10–16 weeks, the negative effects of TDF on maternal renal function or infant's bone mineral density were not statistically significant in our study, which is expected due to the short duration of exposure.

This study has several limitations, including the lack of TAF long-term safety outcomes and TAF data being primarily derived from cohort studies and one small RCT, which are subject to selection bias. Further TAF studies with large sample sizes, including bone mineral density assessment and long-term follow-up, are needed to confirm our findings. Although both regimens theoretically reduce HCC by preventing MTCT, long-term treatments for maternal disease with TAF versus TDF on HCC reduction deserve further investigation.

In conclusion, this study indicates that maternal TDF and TAF prophylaxes are equally effective in reducing MTCT and are without safety concerns in highly viremic mothers. Initiating TDF therapy at gestational weeks of 28 had similar efficacy when compared to the second-trimester approach. For mothers without postpartum treatment indication for CHB, TDF therapy might be discontinued at delivery. This meta-analysis may serve as evidence for future updates on guidelines for the management of CHB.

## Supplementary Data

Supplementary materials are available at *Clinical Infectious Diseases* online. Consisting of data provided by the authors to benefit the reader, the posted materials are not copyedited and are the sole responsibility of the authors, so questions or comments should be addressed to the corresponding author.

## Supplementary Material

ciae288_Supplementary_Data

## Notes

***Author contributions.*** C. Q. P. proposed the review and protocol; L. Z., B. Z., A. S. Y., Y. C. Z., E. H. D., and C. Q. P. contributed to the systematic review and data extraction. C. Q. P., L. Z., and B. Z. analyzed the data and wrote the manuscript. C. Q. P. further revised the manuscript, conducted a critical review, communicated with the journal, and addressed the reviewer's comments. All authors reviewed and approved the final version of the manuscript. The corresponding authors attest that all listed authors meet the authorship criteria and that no others meeting the criteria have been omitted.

***Acknowledgments.*** The authors extend their gratitude to all investigators who contributed to this study.

***Data sharing.*** Data analyzed in this study were collected from published studies, and specific individual participant data were not utilized. All data analyzed in the current study are accessible via the databases listed in our methods section. For access to individual participant data, requests should be directed to the corresponding author of the respective published study.

***Financial support***. This study is funded by the Key Project Plan of Medical Science Research in Hebei Province (grant number 20221686). However, the grant provider had no involvement in the study design, data collection, data analysis, data interpretation, writing of the report, or decision on selecting a journal for publication.
