# Supplementary material for: Tenofovir Alafenamide Versus Tenofovir Disoproxil Fumarate for Preventing Vertical Transmission in Chronic Hepatitis B Mothers: A Systematic Review and Meta-Analysis
Source: Clin Infect Dis. 2024 May 28;79(4):953–64. doi: 10.1093/cid/ciae288 (PMC11478587; doi:10.1093/cid/ciae288)
Supplement: ciae288_Supplementary_Data [file ciae288_supplementary_data.docx]

**Supplemental Online Content**

This appendix has been provided by the authors to give readers additional information regarding their study.

Supplement to: Pan CQ, et al. Tenofovir Alafenamide versus Tenofovir Disoproxil Fumarate for Preventing Vertical Transmission in Chronic Hepatitis B Mothers: A Systematic Review and Meta-Analysis

**CONTENTS**

**Supplementary Appendix 1. Search strategy Page 1**

**Supplementary Appendix 2. Eligibility criteria of included studies Page 6**

**Supplementary Appendix 3. Abstracts and newly published studies Page 7**

**Supplementary Appendix 4. Quality assessment of the included studies Page 10**

Table S1. Assessment of the risk of bias using the Cochrane tools Page 10

Table S2. Assessment of the risk of bias using the Newcastle-Ottawa Scale Page 15

**Supplementary Appendix 5. Maternal and infant characteristics and outcomes Page 29**

Table S3A. Maternal baseline characteristics and the virological responses after maternal

TDF or TAF therapy Page 29

Table S3B. The characteristics of infants at birth and the efficacy outcomes of preventing

HBV vertical transmission Page 33

**Supplementary Appendix 6. Assessment of between-study heterogeneity and the summary**

**effect influenced by a particular study Page 37**

Figure S1. Sensitivity analysis in all TDF studies by omitting each trial one by one Page 37

**Supplementary Appendix 7. Efficacy rank of TAF vs TAF on preventing MTCT estimated**

**by the network meta-analysis…………………………………………………………….Page 38**

Figure S2A. Forest plot for the overall efficacy of TDF vs TDF on preventing MTCT in the

network meta-analysis…………………………………………………………………..Page 38

Figure S2B. The surface under the cumulative ranking curves for TDF vs TAF …Page 39

**Supplementary Appendix 8. Efficacy of antiviral prophylaxis using TDF or TAF by the**

**sub-group analyses………………………………………………………………………Page 40**

Figure S3A. Efficacy of TDF in the prevention of MTCT with different levels of HBV-DNA

at baseline of mothers…………………………………………………………………Page 40

Figure S3B. Efficacy of TDF in the prevention of MTCT with different status of HBeAg of

mothers………………………………………………………………………………..Page 41

Figure S3C. Efficacy of TDF in the prevention of MTCT with different Languages….Page 42

Figure S3D. Efficacy of TDF in the prevention of MTCT with different timing of administrating

birth doses of the HBV vaccine…………………………………………………Page 43

Figure S3E. Efficacy of TDF in the prevention of MTCT with different timing of administrating

birth doses of HBIg ……………………………………………………………....Page 44

**Supplementary Appendix 9. Safety of antiviral prophylaxis using TDF or**

**TAF………………………………………………………………………………………..Page 45**

**Supplementary Appendix 10. Fetal safety of peripartum antiviral prophylaxis using**

**TDF or TAF………………………………………………………………………………Page 49**

Figure S4A. Fetal safety of TDF in fetal death……………………………………… Page 49

Figure S4B. Fetal safety of TAF in fetal death……………………………………… Page 50

Figure S4C. Fetal safety of TDF in Apgar score (1 minute)………………………….Page 51

Figure S4D. Fetal safety of TAF in Apgar score (1 minute) …………………………Page 52

Figure S4E. Fetal safety of TDF in physical growth………………………………….Page 53

Figure S4F. Fetal safety of TAF in physical growth………………………………….Page 54

Figure S4G. Fetal safety of TDF in grade 3 or 4 adverse events……………………...Page 55

Figure S4H. Fetal safety of TDF in bone mineral density scores...…………………...Page 56

Figure S4I. Fetal safety of TDF in the physical growth of long-term observations… Page 57

Figure S4J. Fetal safety of TDF in bone mineral density scores of long-term observations

…………………………………………………………………………………………Page 58

**Supplementary Appendix 11. Maternal safety of peripartum antiviral prophylaxis using**

**TDF or TAF………………………………………………………………………………Page 59**

Figure S5A. Maternal safety of TDF in pregnancy complications……………………Page 59

Figure S5B. Maternal safety of TAF in pregnancy complications……………………Page 60

Figure S5C. Maternal safety of TDF in the elevation of creatine kinase……………...Page 61

Figure S5D. Maternal safety of TDF in postpartum hemorrhage……………………..Page 62

Figure S5E. Maternal safety of TAF in postpartum hemorrhage……………………..Page 63

Figure S5F. Maternal safety of TDF in severe adverse events (grades III and IV)…...Page 64

**Supplementary Appendix 12. Publication bias………………………………………….Page 65**

Figure S6A. Publication Bias Assessment (Funnel Plots) of the primary outcome in TDF

studies………………………………………………………………………………..Page 65

Figure S6B. Publication Bias Assessment (Egger’s test) of the primary outcome in TDF

studies………………………………………………………………………………..Page 66

Figure S6C. Publication Bias Assessment (Funnel Plots) of the primary outcome in TAF

studies………………………………………………………………………………..Page 67

Figure S6D. Publication Bias Assessment (Egger’s test) of the primary outcome in TAF

studies………………………………………………………………………………..Page 68

**Supplementary Appendix 1. Search strategy**

**PubMed, from database inception to Oct 31, 2022**

**Searching terms：**

1. ("Hepatitis B"[Mesh]) OR (Hepatitis B Virus Infection [Title/Abstract])

2. ("Pregnancy"[Mesh]) OR ((Pregnancies [Title/Abstract]) OR (Gestation [Title/Abstract]))

3. ("Pregnant Women"[Mesh]) OR (Pregnancy [Title/Abstract])

4. 2 OR 3

5. ("Infant, Newborn"[Mesh]) OR (((((((Infants, Newborn[Title/Abstract]) OR (Newborn Infant[Title/Abstract])) OR (Newborn Infants[Title/Abstract])) OR (Newborns[Title/Abstract])) OR (Newborn[Title/Abstract])) OR (Neonate[Title/Abstract])) OR (Neonates[Title/Abstract]))

6. 4 OR 5

7. ("Infectious Disease Transmission, Vertical"[Mesh]) OR (((((((((((((((((Vertical Infection Transmission[Title/Abstract]) OR (Vertical Infectious Disease Transmission[Title/Abstract])) OR (Infection Transmission, Vertical[Title/Abstract])) OR (Maternal-Fetal Infection Transmission[Title/Abstract])) OR (Transmission, Vertical Infection[Title/Abstract])) OR (Maternal Fetal Infection Transmission[Title/Abstract])) OR (Transmission, Maternal-Fetal Infection[Title/Abstract])) OR (Infection Transmission, Maternal-Fetal[Title/Abstract])) OR (Infection Transmission, Maternal Fetal[Title/Abstract])) OR (Infection Transmission, Fetomaternal[Title/Abstract])) OR (Mother-to-Child Transmission[Title/Abstract])) OR (Mother to Child Transmission[Title/Abstract])) OR (Mother-to-Child Transmissions[Title/Abstract])) OR (Transmission, Mother-to-Child[Title/Abstract])) OR (Transmissions, Mother-to-Child[Title/Abstract])) OR (Fetomaternal Infection Transmission[Title/Abstract])) OR (Transmission, Fetomaternal Infection[Title/Abstract]))

8. 6 OR 7

9. ("Human hepatitis B virus immunoglobulin" [Mesh] OR (HBIG[Title/Abstract])

10. ("Vaccines" [Mesh] OR (Vaccine [Title/Abstract])

11. ("Antiviral Agents"[Mesh]) OR (((((((((Agents, Antiviral[Title/Abstract]) OR (Antivirals[Title/Abstract])) OR (Antiviral[Title/Abstract])) OR (Antiviral Drugs[Title/Abstract])) OR (Drugs, Antiviral[Title/Abstract])) OR (Antiviral Drug[Title/Abstract])) OR (Drug, Antiviral[Title/Abstract])) OR (Antiviral Agent[Title/Abstract])) OR (Agent, Antiviral[Title/Abstract]))

12. (("Tenofovir"[Mesh]) OR ((((((Tenofovir Disoproxil Fumarate[Title/Abstract]) OR (Disoproxil Fumarate, Tenofovir[Title/Abstract])) OR (Fumarate, Tenofovir Disoproxil[Title/Abstract])) OR (Tenofovir Disoproxil[Title/Abstract])) OR (Disoproxil, Tenofovir[Title/Abstract])) OR (Viread [Title/Abstract]))) OR (TDF[Text Word])

13. (Tenofovir alafenamide [Supplementary Concept]) OR (TAF [Text Word])

14. 9 OR 10 OR 11 OR 12 OR 13

15. 1 AND 8 AND 14

**Embase, from database inception to Oct 31, 2022**

**Searching terms：**

1. ' hepatitis b'/exp OR ' hepatitis b virus infection': ab, ti

2. 'pregnancy'/exp OR ('pregnancies': ab,ti OR 'gestation':ab, ti) OR 'pregnant woman'/exp OR 'pregnancy':ab,ti

3. 'newborn'/exp OR ('infants, newborn': ab,ti OR 'newborn infant':ab, ti OR 'newborn infants':ab,ti OR 'newborns':ab,ti OR 'newborn':ab,ti OR 'neonate':ab,ti OR 'neonates':ab,ti )

4. 2 OR 3

5. 'vertical transmission'/exp OR ('vertical infection transmission':ab,ti OR 'vertical infectious disease transmission':ab,ti OR 'infection transmission, vertical':ab,ti OR 'transmission, vertical infection':ab,ti OR 'maternal-fetal infection transmission':ab,ti OR 'maternal-fetal infection transmission':ab,ti OR 'transmission, maternal-fetal infection':ab, ti OR infection transmission, maternal-fetal':a b, ti OR 'infection transmission, maternal-fetal': ab ti OR 'infection transmission, fetomaternal: ab ti OR 'mother-to-child transmission ': ab,t I OR 'mother to child transmission n':ab, ti OR 'mother-to-child transmissi ons': ab, ti OR 'transmission, mother-to-child':ab, ti OR 'transmissions, mother-to-c hild':ab, ti OR 'fetomaternal infection transmission': ab, ti OR 'transmission, fetomaternal infection': ab, ti)

6. 'antivirus agent'/exp OR ('agents, antiviral':ab, ti OR 'antivirals':ab, ti OR 'antiviral':ab, ti OR 'antiviral drugs':ab, ti OR 'drugs, antiviral':a b, ti OR 'antiviral drug': ab, ti OR 'drug, antiviral': ti OR 'antiviral agent': ab, ti OR 'agent, antiviral, ti)

7. 'tenofovir'/exp OR('tenofovir disoproxil fumarate':ab,ti OR 'disoproxil fumarate, tenofovir':ab,ti OR 'fumarate, tenofovir disoproxil':ab,ti OR 'tenofovir disoproxil':ab,ti OR 'disoproxil, tenofovir':ab,ti OR 'viread':ab,ti) OR tdf

8. 'tenofovir alafenamide'/exp OR taf

9. 6 OR 7 OR 8

10. 1 AND 4 AND 5 AND 9

**Cochrane library, from database inception to Oct 31, 2022**

**Searching terms：**

#1 MeSH descriptor: [Hepatitis B] explode all trees

#2 (Hepatitis B Virus Infection): ti,ab,kw

#3 #1 OR #2

#4 MeSH descriptor: [Vaccines] explode all trees

#5 (Vaccines): ti,ab,kw

#6 #4 OR #5

#7 MeSH descriptor: [Pregnancy] explode all trees

#8 (Pregnancies): ti,ab,kw OR (Gestation): ti,ab,kw

#9 #7 OR #8

#10 MeSH descriptor: [Pregnant Women] explode all trees

#11 (Pregnancy): ti,ab,kw

#12 #10 OR #11

#13 #9 OR #12

#14 MeSH descriptor: [Infant] explode all trees

#15 (Infants, Newborn):ti,ab,kw OR (Newborn Infant):ti,ab,kw OR (Newborn Infants):ti,ab,kw OR (Newborns):ti,ab,kw OR (Newborn):ti,ab,kw OR (Neonate):ti,ab,kw OR (Neonates):ti,ab,kw

#16 #14 OR #15

#17 #13 OR #16

#18 MeSH descriptor: [Infectious Disease Transmission, Vertical] explode all trees

#19 (Vertical Infection Transmission): ti,ab,kw OR (Vertical Infectious Disease Transmission):ti,ab,kw OR (Infection Transmission, Vertical):ti,ab,kw OR (Transmission, Vertical Infection):t I, ab,kw OR (Maternal-Fetal Infection Transmission):ti,ab,kw OR (Maternal Fetal Infection Transmission):ti,ab,kw OR (Transmission, Maternal-Fetal Infection):ti,ab,kw OR (Infection Transmission, Maternal-Fetal):ti,ab,kw OR (Infection Transmission, Maternal Fetal):ti,ab,kw OR (Infection Transmission, Fetomaternal):ti,ab,kw OR (Mother-to-Child Transmission):ti,ab,kw OR (Mother to Child Transmission):ti,ab,kw OR (Mother-to-Child Transmissions):ti,ab,kw OR (Transmission, Mother-to-Child):ti,ab,kw OR (Transmissions, Mother-to-Child):ti,ab,kw OR (Fetomaternal Infection Transmission):ti,ab,kw OR (Transmission, Fetomaternal Infection):ti,ab,kw

#20 #18 OR #19

#21 #17 OR #20

#22 MeSH descriptor: [Antiviral Agents] explode all trees

#23 (Agents, Antiviral):ti,ab,kw OR (Antivirals):ti,ab,kw OR (Antiviral):ti,ab,kw OR (Antiviral Drugs):ti,ab,kw OR (Drugs, Antiviral):ti,ab,kw OR (Antiviral Drug):ti,ab,kw OR (Drug, Antiviral):ti,ab,kw OR (Antiviral Agent):ti,ab,kw OR (Agent, Antiviral):ti,ab,kw

#24 #22 OR #23

#25 MeSH descriptor: [Tenofovir] explode all trees

#26 (Tenofovir Disoproxil Fumarate):ti,ab,kw OR (Disoproxil Fumarate, Tenofovir):ti,ab,kw OR (Fumarate, Tenofovir Disoproxil):ti,ab,kw OR (Tenofovir Disoproxil):ti,ab,kw OR (Disoproxil, Tenofovir):ti,ab,kw OR (Viread):ti,ab,kw

#27 #25 OR #26

#28 (TDF) (Word variations have been searched)

#29 #27 OR #28

#30 (tenofovir alafenamide) OR (TAF) (Word variations have been searched)

#31 #24 OR #29 OR #30

#32 #6 OR #31

#33 #3 AND #21 AND #32

**CNKI, from database inception to Oct 31, 2022**

**Searching terms：**

1. SU %= '乙型肝炎' OR SU %= '乙肝病毒感染' OR SU %= 'HBV' OR SU %= '乙型病毒性肝炎'

2. SU %= '妊娠' OR SU %= '怀孕' OR SU %= '孕妇' OR SU %= '婴儿' OR SU %= '新生儿' OR SU %= '垂直传播' OR SU %= '母婴传播' OR SU %= '母婴传播阻断' OR SU %= '母婴感染' OR SU %= '血液传播'

3. (SU %= '乙肝疫苗' OR SU %= '免疫球蛋白' OR SU %= '乙肝免疫球蛋白' OR SU %= 'HBIG'） OR (SU %= ' 抗病毒' OR SU %= '抗病毒治疗' OR SU %= '抗病毒药物' OR SU %= '核苷酸类似物' OR SU %= '替诺福韦' OR SU %= '替诺福韦酯' OR SU %= '富马酸替诺福韦二吡呋酯' OR SU %= 'TDF' OR SU %= '丙酚替诺福韦' OR SU %= '替诺福韦艾拉酚胺' OR SU %= 'TAF')

4. 1 AND 2 AND 3

**Wanfang, from database inception to Oct 31, 2022**

**Searching terms：**

1. 主题:(乙型肝炎) or 主题:(乙型病毒型肝炎) or 主题:(乙肝病毒感染) or 主

题:(HBV)

2. 主题:(妊娠) or 主题:(怀孕) or 主题:(孕妇) or 主题:(婴儿) or 主题:(新生儿) or 主题:(垂直传播) or 主题:(母婴传播) or 主题:(母婴传播阻断) or 主题:(血液传播) or 主题:(母婴感染)

3. 主题:(乙肝疫苗) or 主题:(免疫球蛋白) or 主题:(乙肝免疫球蛋白) or 主题: (HBIG) or 主题:(抗病毒) or 主题:(抗病毒治疗) or 主题:(抗病毒药物) or 主题:(核苷酸类似物) or 主题:(替诺福韦) or 主题:(替诺福韦酯) or 主题:(富马酸替诺福韦二吡呋酯) or 主题:(TDF) or 主题:(丙酚替诺福韦) or 主题:(替诺福韦艾拉酚胺) or 主题: (TAF)

4. 1 AND 2 AND 3

**Supplementary Appendix 2. Eligibility criteria of included studies**

|  | **Inclusion criteria** | **Exclusion criteria** |
| --- | --- | --- |
| **Participants** | Pregnant women with high levels of HBV DNA. | Patients are confused with hepatitis C, hepatitis D, or HIV. |
| **Intervention** | Mothers: TDF or TAF during pregnancy.  Infants: received immunization during the first week postpartum. | Mothers: Using other antiviral therapy during pregnancy. Patients receiving steroids, chemotherapy/immunotherapy, liver transplantation, and hemodialysis.  Infants: didn’t receive immunization during the first week postpartum. |
| **Comparators** | Mothers: received no intervention or placebo. | Mothers: received other antiviral treatment. |
| **Outcomes** | Primary outcome: MTCT, indicated by infant HBsAg positivity or HBV DNA positivity, or both, at age 6–12 months.  Secondary outcomes: Infants or maternal adverse events were reported. | No relevance with MTCT. |
| **Study**  **design/Settings** | RCTs and Non-RCTs of interventions were eligible if they were described as prospective or retrospective cohort studies, with control populations. | Uncontrolled studies or studies published as abstracts only. Non-randomized studies with a high risk of bias on the Newcastle-Ottawa Scale (ie, a score of ≤5). |

**Supplementary Appendix 3. Abstracts and newly published studies**

| **Title** | **Author (Year)** | **Conferences/Journals** | **Study Design** | **Groups** | **Sample Size (Mothers and Infants)** | **Principal Findings** |
| --- | --- | --- | --- | --- | --- | --- |
| Tenofovir-DF therapy prevents hepatitis B vertical transmission in highly viremic mothers without HBV immunoglobulin (HBIG) for infants | Pan CQ, et al. (2022) | AASLD | RCT | TDF (gestational week 16) vs TDF (gestational week 28) | Mothers: 128 vs 137  Infants: 128 vs 141 | - The median duration of the TDF treatment was 23 weeks and 11 weeks for mothers in the experimental and comparator groups (p <0.001), respectively. - At delivery, the maternal median [IQR] HBV-DNA level (log10 IU/ml) was significantly lower in the experimental group (2.4 [1.9, 3.0] vs 3.6 [2.9, 4.6]; p <0.001). - The per-protocol, last observation carried forward, and sensitivity analyses showed that transmission rates did not differ significantly between groups. - The congenital defect rates were similar between groups (Experimental-2.3% [3/131] vs Comparator-6.3% [9/142; p=0.10). |
| The Comparison of tenofovir alafenamide fumarate with tenofovir disoproxil fumarate in preventing hepatitis B transmission in mothers with high viral load: a retrospective cohort study | Zhu YX, et al. (2022) | EASL | Retrospective  cohort study | TAF vs TDF | Mothers: 51 vs 51  Infants: 51 vs 51 | - The levels of HBV-DNA decline in TDF-treated mothers were compared to TAF-treated mothers (3.70 ± 0.91 log10IU/ml vs 3.43 ± 1.30 log10IU/ml, P > 0.05) before delivery. - The rates of MTCT in the TAF group were similar to the TDF group (with the transmission of 0.00% [0 of 39] vs 0.00% [0 of 29], p > 0.05) in the per-protocol analysis. - As for BMD, no significant difference in the Z value of newborns was found between the two groups. - However, the expression level of ALP was significantly higher in the TDF group than TAF group. |
| Tenofovir alafenamide in blocking the mother-to-child transmission of hepatitis B virus: A multi-center, prospective clinical study | Han GR, et al. (2022) | J Matern Fetal Neonatal Med. | Multi-center, prospective study; single-arm study | TAF | Mothers: 89  Infants: 91  (two sets of twins) | - The HBsAg positive rate was 0% at seven months in 91 infants, with no growth retardation and congenital defects. - At delivery, 82.02% (73/89) mothers achieved HBV DNA < 200,000 IU/mL, and 21.35% (19/89) achieved HBV DNA < 500 IU/ml. - Nine of 82 mothers who stopped TAF treatment after delivery had mild ALT elevation. |

**Supplementary Appendix 4. Quality assessment of the included studies**

Supplementary Table S1. Assessment of the risk of bias using the Cochrane tools on RCT studies

| **Author (Year)** | **Selection bias** | | **Performance bias** | **Detection bias** | **Attrition bias** | | | **Reporting bias** |
| --- | --- | --- | --- | --- | --- | --- | --- | --- |
|  | **Random sequence generation** | **Allocation concealment** | **Blinding of participants, personnel** | **Blinding of outcome assessment** | **Incomplete outcome data addressed** | | | **Selective reporting** |
|  |  |  |  |  | **MTCT** | **Infant Safety** | **Mother safety** |  |
| **Maternal TDF therapy (300mg orally once a day)** | | | | | | | | |
| Pan CQ et al. (2016) | Low risk | High risk | High risk | High risk | Low risk | Low risk | Low risk | Low risk |
|  | Described as follows： Enrollment at each center was performed with the use of blocks and randomized for sample balance. Using a randomization table, we randomly assigned 200 mothers, in a 1:1 ratio. | Comment: no relevant description. | Described as follows: open-label. | Described as follows: open-label. | Described as follows: Loss to follow-up is detailed carefully in Figure 1(95% the in treated growth p, 88% in the control group). | Comment: Reported incidence and proportion of adverse events in infants. | Comment: Reported incidence and proportion of adverse events in mothers. | Comment: There is negative event reporting and no tendency to report selectively. |
| Jourdain G et al. (2018) | Low risk | Low Risk | Low Risk | Low Risk | Low Risk | Low risk | High risk | Low risk |
|  | Described as follows： Participants were randomly assigned in a 1:1 ratio (permuted blocks ). | Described as follows： The participants, the trial staff on site and at the coordination center, the investigators, and the laboratory personnel were unaware of the trial-group assignments. | Described as follows： matching placebo (similar to active tablets minus the active pharmaceutical ingredient)” | Described as follows： The participants, the trial staff on site and at the coordination center, the investigators, and the laboratory personnel were unaware of the trial_x0002_group assignments.” | Comment: Follow-up rates and reasons for loss to follow-up are documented in detail | Comment: All relevant adverse events addressed, including growth | Comment: Some important adverse events were not recorded and only common adverse events such as elevated transaminases were described | Comment: The protocol is available online. The current outcomes of interest in a recording were pre-specified. |
| Lin Y et al. (2018) | Low risk | Low risk | Unclear risk | Low risk | High risk | High risk | High risk | Low risk |
|  | Described as follows： A random number table was used to group the pregnancies into each group (60 individuals per group) based on their enrollment time. Simple randomization was performed… | Described as follows: Sealed envelopes were used for concealment of the random allocation. | Comment: The article mentioned that the participants did not know whether they received the intervention, but did not say whether the control group was also unaware | Described as follows: Persons who examined the viral DNA loads and evaluated the outcomes of the patients did not know whether the patients had accepted the intervention.” | Comment: The follow-up rate was only 87% in the control group and 100% in the experimental group, and no reason for loss to follow-up was described. | Comment: Safety results not described in detail | Comment: Safety results not described in detail | Comment: The protocol is available online. The current outcomes of interest in a recording were pre-specified. |
| Huang XL（2023） | Low risk | Unclear risk | High risk | High risk | Low Risk | Low Risk | Low Risk | Low risk |
|  | According to the random number table method, they were divided into a study group and a control group, 44 cases each. | Comment: no concealment described | Pregnant women in the control group received health guidance during pregnancy | Comment: No description | The follow-up rate and the MTCT rate were recorded in detail | Neonatal and perinatal adverse events were recorded | Adverse reactions in pregnant women were recorded | Comment: there is negative event reporting, and there is no tendency to selectively report. |
| Liu M et al. (2017) | Low risk | Unclear risk | Unclear risk | High risk | Low Risk | Low Risk | Low risk | Low risk |
|  | Described as follows： participants were randomly assigned in a 1:1 ratio. | Comment: no concealment described | Described as follows： The control group did not receive any antiviral treatment. | Comment: It is easy for analysts to know what treatment a pregnant woman is receiving | Comment: Infants delivered by mothers in both the TDF group and the control group completed postpartum follow-up。 | Common adverse events were reported | Comment: There were no important adverse events in the mothers during the medication and labor. | Comment: There is negative event reporting, and there is no tendency to report selectively. |
| **Maternal TAF therapy (25mg orally once a day)** | | | | | | | | |
| Li B et al. (2021) | Low risk | High risk | High risk | High risk | Low Risk | Low Risk | Low Risk | Low risk |
|  | Described as follows： Pregnant women (n=72) were randomly divided into two groups | Comment: no concealment described | Comment: no described | Comment: No description, it is easy for the evaluator to know how the pregnant woman was treated | Comment: Infants delivered by mothers in both groups completed postpartum follow-up。 | Common adverse events were reported | Describes the occurrence of some important events | Comment: The protocol is available online. The current outcome of interest was pre-specified |

Supplementary Table S2. Assessment of the risk of bias using the Newcastle-Ottawa Scale in non-RCT studies

| **Author (Year)** | **Representativeness of the exposed cohort** | **Selection of the non-**exposed cohort | **Ascertainment of exposure** | **Demonstration that outcome of interest was not present at baseline** | **Comparability of cohorts based on the design or analysis** | **Assessment of outcomes** | **Was follow-up long enough for outcomes to occur** | **Adequacy of follow-up of cohorts** | **Total number of stars (risk of bias)** |
| --- | --- | --- | --- | --- | --- | --- | --- | --- | --- |
| **Maternal TDF therapy (300mg orally once a day)** | | | | | | | | | |
| Chen H et al. (2015) | 1 | 1 | 1 | 1 | 2 | 1 | 1 | 1 |  |
|  | At least somewhat representative of the average HBV-infected pregnant woman. | Drawn from the same community (same inclusion and exclusion criteria also). | Regular testing (and pre-delivery testing) of HBV DNA levels were correlated with the duration of treatment in mothers. | Always | Comparable for HBV DNA level and comparable HBeAg positive. The same regimen for infant immunoprophylaxis. | Describes test assays used for HBsAg and HBV DNA and acknowledges a study laboratory. | Yes | LFU reported and<20% LFU in all treatment and control groups. | 9 (low) |
| Samadi K et al. (2016) | 1 | 0 | 1 | 1 | 1 | 1 | 1 | 1 |  |
|  | At least somewhat representative of the average HBV-infected pregnant woman. | Not the same population, the untreated did not have high viremia or pre-existing liver disease, whereas the treated did. | all women are screened for HBV using HBsAg serology and tested. | Always | Not comparable for HBV DNA level or HBeAg positive. However, the immunization regimen of infants was the same. | Unified detection and use of the same method. | Yes | <80% follow-up in both treated and control groups. | 6 (high) |
| Chen WJ et al. (2017) | 1 | 1 | 1 | 1 | 2 | 1 | 1 | 0 |  |
|  | At least somewhat representative of the average HBV-infected pregnant woman. | Drawn from the same community (same inclusion and exclusion criteria also). | The HBV DNA levels of mothers were tested before taking the drug, 6 weeks after taking the drug, and before delivery. | Always | Comparable for HBV DNA level and comparable HBeAg positive. The same regimen for infant immunoprophylaxis. | Uniform detection reagents and description of detection methods. | Yes | No description of loss to follow-up. | 8 (low) |
| Greenup AJ et al. (2014) | 1 | 1 | 1 | 1 | 2 | 0 | 1 | 0 |  |
|  | At least somewhat representative of the average HBV-infected pregnant woman. | Drawn from the same community (same inclusion and exclusion criteria also). | Reporting on adherence within the paper, reduction of viral load used to assess women's response to treatment. | Always | Comparable for HBV DNA level and comparable HBeAg positive. The same regimen for infant immunoprophylaxis and confirmation that all infants received it. | No details were given on laboratory methods for infants, and no details of which assay was used for testing HBsAg. | Yes | The control group lost more to follow-up, more than 20%. | 7 (low) |
| Celen MK et al. (2013) | 1 | 1 | 0 | 1 | 2 | 1 | 1 | 0 |  |
|  | At least somewhat representative of the average HBV-infected pregnant woman. | Drawn from the same community (same inclusion and exclusion criteria also). | Do not provide many details on the decrease of HBV DNA level and another discussion of maternal adherence. | Always | Comparable for HBV DNA level and comparable HBeAg positive. The same regimen for infant immunoprophylaxis. | Describes testing done and refers to a central laboratory employed for this study. | Yes | None reported(retrospective) | 7 (low) |
| Wang YC et al. (2020) | 1 | 1 | 1 | 1 | 2 | 1 | 1 | 1 |  |
|  | At least somewhat representative of the average HBV-infected pregnant woman. | Drawn from the same community (same inclusion and exclusion criteria also). | HBVDNA, calcium, phosphorus, and other indicators were detected at the 28th week of pregnancy and within 24h postpartum in both groups. | Always | Comparable for HBV DNA level and comparable HBeAg positive. The same regimen for infant immunoprophylaxis. | Use uniform detection reagents and uniform determination by laboratory personnel. | Yes | Complete follow-up | 9 (low) |
| Chen CY et al. (2019) | 1 | 1 | 1 | 1 | 2 | 1 | 1 | 0 |  |
|  | At least somewhat representative of the average HBV-infected pregnant woman. | Drawn from the same community (same inclusion and exclusion criteria also). | Pregnant women in the treatment group were tested for HBV DNA and other indicators at 18, 24, and 36 weeks of treatment. | Always | Comparable for HBV DNA level and comparable HBeAg positive. The same regimen for infant immunoprophylaxis. | Use uniform detection reagents and equipment. | Yes | No description of loss to follow-up. | 8 (low) |
| Shen GJ et al. (2021) | 1 | 1 | 1 | 1 | 1 | 0 | 1 | 0 |  |
|  | At least somewhat representative of the average HBV-infected pregnant woman | Drawn from the same community (same inclusion and exclusion criteria also) | Serum HBV DNA was detected in the two groups of pregnant women before taking the drug, during delivery, and after delivery. | Always | Comparable for HBV DNA level and the same regimen for infant immunoprophylaxis. However, the HBeAg situation is not stated. | no detailed description | Yes | No description of loss to follow-up. | 6 (high) |
| Zhang JM et al. (2021) | 1 | 1 | 1 | 1 | 1 | 1 | 1 | 0 |  |
|  | At least somewhat representative of the average HBV-infected pregnant woman. | Drawn from the same community (same inclusion and exclusion criteria also). | The levels of serum HBV-DNA and liver function were detected in the two groups of pregnant women at 24 weeks of gestation, before delivery, 4 weeks after delivery, 12 weeks after delivery, and 24 weeks after delivery. | Always | Comparable for HBV DNA level and the same regimen for infant immunoprophylaxis. However, the HBeAg situation is not stated. | The detection method was unified, but it did not say whether the same reagent was used and whether the detection was unified. | Yes | Safety of follow-up infants 28 weeks after delivery not described. | 6 (high) |
| Liu JF et al. (2019) | 1 | 1 | 1 | 1 | 1 | 1 | 1 | 1 |  |
|  | At least somewhat representative of the average HBV-infected pregnant woman. | Drawn from the same community (same inclusion and exclusion criteria also). | The patients with continued treatment also received  follow-up at 12 weeks, 24 weeks, 28 weeks, and 52 weeks after delivery. | Always | Comparable for HBV DNA level and the same regimen for infant immunoprophylaxis. However, the HBeAg situation is not stated. | Using unified testing reagents and testing in the same laboratory. | Yes | 3 cases were lost to follow-up, and the reasons for the loss to follow-up were introduced. | 8 (low) |
| Chang K et al. (2019) | 1 | 1 | 1 | 1 | 2 | 1 | 1 | 1 |  |
|  | At least somewhat representative of the average HBV-infected pregnant woman. | Drawn from the same community (same inclusion and exclusion criteria also). | Pregnant women have regular check-ups. | Always | Comparable for HBV DNA level and comparable HBeAg positive. The same regimen for infant immunoprophylaxis. | Describes testing done and refers to a central laboratory employed for this study. | Yes | There was one case lost to follow-up in both groups, and the loss rate was less than 20%. | 9 (low) |
| Wang Y et al. (2019) | 1 | 1 | 1 | 1 | 2 | 1 | 1 | 1 |  |
|  | At least somewhat representative of the average HBV-infected pregnant woman. | Drawn from the same community (same inclusion and exclusion criteria also). | Serum HBV DNA levels were observed in all pregnant women before medication, during delivery, and 12 weeks postpartum. | Always | Comparable for HBV DNA level and comparable HBeAg positive. The same regimen for infant immunoprophylaxis. | Using unified reagents and detection equipment. | Yes | 2 cases were lost to follow-up in the treatment group (2/128). | 9 (low) |
| Mao C et al. (2019) | 1 | 1 | 1 | 1 | 1 | 0 | 1 | 0 |  |
|  | At least somewhat representative of the average HBV-infected pregnant woman. | Drawn from the same community (same inclusion and exclusion criteria also). | The researchers regularly observe the detection indicators. | Always | Comparable for HBV DNA level and the same regimen for infant immunoprophylaxis. However, the HBeAg situation is not stated. | Introduced the detection method and some instruments and equipment but did not say whether the detection was unified. | Yes | No safety data for infants 28 weeks after delivery. | 6 (high) |
| Ma L et al. (2019) | 1 | 1 | 1 | 1 | 1 | 0 | 1 | 0 |  |
|  | At least somewhat representative of the average HBV-infected pregnant woman. | Drawn from the same community (same inclusion and exclusion criteria also). | All pregnant women with positive HBV markers were included, and HBV DNA, HBsAg, and HBeAg were detected at the time of enrollment and delivery. | Always | Pregnant women with positive HBV markers at baseline, and infants on the same immunization regimen. | The detection method and some detection reagents are introduced, and it was not described whether the detection was unified. | Yes | No safety data for infants 6 months after delivery. | 6 (high) |
| Gao X et al. (2020) | 1 | 1 | 1 | 1 | 2 | 0 | 1 | 0 |  |
|  | At least somewhat representative of the average HBV-infected pregnant woman. | Drawn from the same community (same inclusion and exclusion criteria also). | The study was a retrospective cohort study, collecting data at baseline, before delivery, and 28 weeks after delivery. | Always | Comparable for HBV DNA level and comparable HBeAg positive. The same regimen for infant immunoprophylaxis. | Although the data came from the same hospital, the testing reagents, instruments, and methods were not described, so it was impossible to determine whether they were unified. | Yes | No safety data for infants 6 months after delivery. | 6 (high) |
| Zeng J et al. (2019) | 1 | 1 | 1 | 1 | 2 | 1 | 1 | 1 |  |
|  | At least somewhat representative of the average HBV-infected pregnant woman. | Drawn from the same community (same inclusion and exclusion criteria also). | Investigators regularly observed and checked indicators. | Always | Comparable for HBV DNA level and comparable HBeAg positive. The same regimen for infant immunoprophylaxis. | Using unified reagents and detection equipment. | Yes | Low dropout rate. | 9 (low) |
| Ye Z et al. (2021) | 1 | 1 | 1 | 1 | 1 | 0 | 1 | 0 |  |
|  | At least somewhat representative of the average HBV-infected pregnant woman. | Drawn from the same community (same inclusion and exclusion criteria also). | The protocol for the observed changes in HBV-DNA load in the two groups of pregnant women was not detailed. | Always | Comparable for HBV DNA level and the same regimen for infant immunoprophylaxis. However, the HBeAg situation is not stated. | no detailed description. | Yes | No safety data for infants 6 months after delivery. | 6 (high) |
| Kuang C et al. (2021) | 1 | 1 | 0 | 1 | 2 | 0 | 1 | 0 |  |
|  | At least somewhat representative of the average HBV-infected pregnant woman. | Drawn from the same community (same inclusion and exclusion criteria also). | no other discussion of maternal adherence. | Always | Comparable for HBV DNA level and comparable HBeAg positive. Specific regimens for immunoprophylaxis were not stated. | No testing details for each outcome, including reagents, equipment, and methods. | Yes | No safety results for mothers and infants. | 6 (high) |
| Cui D et al. (2021) | 1 | 1 | 0 | 1 | 1 | 0 | 1 | 1 |  |
|  | At least somewhat representative of the average HBV-infected pregnant woman. | Drawn from the same community (same inclusion and exclusion criteria also). | The study is a retrospective cohort study, collecting data according to time points, without specifying the details to ensure the integrity of data collection. | Always | Comparable for HBV DNA level and the same regimen for infant immunoprophylaxis. However, the HBeAg situation is not stated. | The method of index measurement was the same, and it was not stated whether it was uniformly detected. | Yes | Low dropout rate. | 6 (high) |
| Ran R et al. (2021) | 1 | 0 | 1 | 1 | 1 | 0 | 1 | 1 |  |
|  | At least somewhat representative of the average HBV-infected pregnant woman | This study mainly talks about the efficacy of TDF with different initiation time | The study was a retrospective cohort study, collecting data at baseline, before delivery, and 28 weeks after delivery. | Always | Comparable for HBV DNA level and comparable HBeAg positive. The same regimen for infant immunoprophylaxis. | No testing details for each outcome, including reagents, equipment, and methods. | Yes | Low dropout rate. | 6 (high) |
| Hu MF et al. (2018) | 1 | 1 | 1 | 1 | 1 | 0 | 1 | 1 |  |
|  | At least somewhat representative of the average HBV-infected pregnant woman | Drawn from the same community (same inclusion and exclusion criteria also) | The study was a retrospective cohort study, collecting data at baseline, before delivery, and 28 weeks after delivery. | Always | Comparable for HBV DNA level and comparable HBeAg positive. The same regimen for infant immunoprophylaxis. | No testing details for each outcome, including reagents, equipment, and methods. | Yes | Low dropout rate. | 7 (low) |
| Wang HB et al. (2018) | 1 | 1 | 1 | 1 | 1 | 0 | 1 | 1 |  |
|  | At least somewhat representative of the average HBV-infected pregnant woman | Drawn from the same community (same inclusion and exclusion criteria also) | The study was a retrospective cohort study, collecting data at baseline, before delivery, and 28 weeks after delivery. | Always | Comparable for HBV DNA level and comparable HBeAg positive. The same regimen for infant immunoprophylaxis. | No testing details for each outcome, including reagents, equipment, and methods. | Yes | Low dropout rate. | 7 (low) |
| **Maternal TAF therapy (25mg orally once a day)** | | | | | | | | | |
| Zeng Q et al. (2021) | 1 | 1 | 1 | 1 | 1 | 0 | 1 | 1 |  |
|  | At least somewhat representative of the average HBV-infected pregnant woman | Drawn from the same community (same inclusion and exclusion criteria also) | 232 mothers were eventually enrolled 1:1 for treatment with TAF or TDF | Always | Comparable for HBV DNA level and the same regimen for infant immunoprophylaxis. However ut the HBeAg situation is not stated | not described | Yes | Low dropout rate | 7 (low) |
| Zeng Q et al. (2021) | 1 | 1 | 1 | 1 | 1 | 0 | 1 | 1 |  |
|  | At least somewhat representative of the average HBV-infected pregnant woman. | Drawn from the same community (same inclusion and exclusion criteria also). | The TAF and TDF selections were based on the pregnant women’s personal or their  family’s preferences. | Always | Comparable for HBV DNA level and the same regimen for infant immunoprophylaxis. However, the HBeAg situation is not stated. | not described | Yes | Low dropout rate. | 7 (low) |
| Pan SF et al.(2024) | 1 | 1 | 1 | 1 | 2 | 0 | 1 | 1 |  |
|  | At least somewhat representative of the average HBV-infected pregnant woman | Drawn from the same community(same inclusion and exclusion criteria also) | Venous blood was collected before, during delivery, and 1 and 2 months after treatment, and HBV DNA and liver function were measured, respectively. A total of 96 pregnant women were divided into three groups according to their options: LdT, TAF, and TDF. | Always | Comparable for HBV DNA level 、ALT level and comparable HBeAg positive.The same regimen for infant immunoprophylaxis. | not described | Yes | Based on the inclusion and exclusion criteria, we  included 123 patients, 27 were lost in the follow‐up  phase due to various reasons such as COVID‐19. | 8(low) |

**Supplementary Appendix 5. Maternal and infant characteristics in the studies as well as treatment outcomes**

Supplementary Table S3A. Maternal baseline characteristics and the virological responses after maternal TDF or TAF therapy

| **Author**  **(Year)** | **Country** | **Study design**  **(RCT/non-RCT)** | **Time**  **period** | **Pregnant**  **Women in treatment/control groups,**  **N** | **Inclusion criteria for HBeAg/HBV-DNA (log10 IU/mL)** | **Initiation/discontinuation times of treatment, wk** | **Age,**  **Mean±SD or median (range) in treatment versus control groups** | **Mean/Median HBV-DNA**  **(log10 IU/mL) at baseline in treatment versus control groups** | **Mean/Median HBV-DNA**  **(log10 IU/mL) at delivery** **in treatment versus control groups** | **HBV DNA suppression at delivery, %(N) in treatment versus control groups** | **Alanine aminotransferase (ALT)**  **normalization**  **%(N) in treatment versus control groups** | **HBeAg** **loss,**  **%(N) in treatment versus control groups** | **HBeAg seroconversion, %(N) in treatment versus control groups** |
| --- | --- | --- | --- | --- | --- | --- | --- | --- | --- | --- | --- | --- | --- |
| **Maternal TDF therapy (300mg orally once a day) vs Placebo** | | | | | | | | | | | | | |
| Chen H et al. (2015) | China (Taiwan) | Non-RCT | 2011 － 2013 | 62/56 | positive/≥7.5 | 30-32/4 (postpartum) | 32.5 ± 3.2 vs 32.4 ± 3.1 | 8.25 ± 0.45 vs 8.24 ± 0.35 | 4.29 ± 0.93 vs 8.1 ± 0.56 | 98.39% (61/62) vs 1.79% (1/56) | 4.84% (3/62) vs 0% (0/56) | NR | 4.84% (3/62) vs 0% (0/56) |
| Pan C et al. (2016) | China | RCT | 2012 － 2013 | 97/100 | positive/≥5.0 | 30-32/4 (postpartum) | 27.4 ± 3.0 vs 26.8 ± 3.0 | 8.2 ± 0.5 vs 8.0 ± 0.7 | 4.7 (4.1 – 5.3) vs 8.0 (7.5 – 8.3) | 68% (66/97) vs 2% (2/100) | NR | 0% (0/97) vs 4% (4/100) | 1.03% (1/97) vs 0% (0/100) |
| Samadi K et al. (2016) | Canada | Non-RCT | 2011 － 2014 | 23/138 | Both/>7.7 | 28-32/12 (postpartum) | 30 (28, 34) vs 32 | 7.7 (3.2 – 8.1) vs 2.3 (1.6 – 3.1) | 2.3 vs 2.7 | 43.48% (10/23) vs 0% (0/138) | 43.48% (10/23) vs. 0% (0/146) | NR | NR |
| Liu M et al. (2017) | China | RCT | 2014 － 2016 | 20/20 | positive/≥5.0 | 28-30/At delivery | 30 (22, 38) vs 29 | 6.51±0.91 vs 6.47 ± 1.00 | NR | 90% (18/20) vs 15% (3/20) | 90% (18/20) vs 65% (13/20) | NR | 10% (2/20) vs 0% (0/20) |
| Chen W et al. (2017) | China | Non-RCT | 2014 － 2015 | 30/44 | positive/≥6.0 | 28/At delivery | 28.7 ± 5.7 vs 29.9 ± 5.1 | 7.5 ± 0.5 vs 7.5 ± 0.55 | 2.86 ± 0.39 vs 7.59 ± 0.7 | NR | NR | NR | NR |
| Jourdain G et al. (2018) | Thailand | RCT | 2013 － 2015 | 152/154 | positive/NR | 28/8 (postpartum) | 25.5 (22.6, 29.1) vs 26.7 | 7.6 ± 1.5 vs 7.3 ± 1.7 | 4.0 ± 1.6 vs 7.3 ± 1.7 | NR | NR | NR | NR |
| Lin Y et al. (2018) | China | RCT | 2013 － 2016 | 59/52 | positive/≥6.0 | 24/4 (postpartum) | 28.3 ± 3.6 vs 28.1 ± 3.4 | 7.44 ± 8.0 vs 7.66 ± 0.55 | 2.54 ± 0.60 vs 7.60 ± 0.60 | 90% (53/59) vs 0% (0/52) | NR | NR | NR |
| Greenup AJ et al. (2014) | Australia | Non-RCT | 2007 － 2010 | 58/20 | Both/>7.0 | 32/12 (postpartum) | NR | 7.8 vs NR | 4.4 vs NR | 98.28% (57/58) vs 0% (0/20) | NR | NR | NR |
| Celen MK et al. (2013) | Turkey | Non-RCT | 2010 － 2012 | 21/24 | positive/≥7.0 | 18-27/4 (postpartum) | 28.2 ± 4.1 vs 26.9 ± 2.9 | 8.28 vs 8.31 | NR | 61.90% (13/21) vs 0% (0/24) | 80.95% (17/21) vs 62.50% (15/24) | NR | NR |
| Wang YC et al. (2020) | China | Non-RCT | 2014 － 2017 | 72/56 | positive/≥6.0 | 28/NR | 25.6 ± 7.1 vs 24.95 ± 6.1 | 7.32 ± 5.78 vs 7.24 ± 4.96 | NR | 69.44% (50/72) vs 0% (0/56) | NR | NR | NR |
| Chen CY et al. (2019) | China | Non-RCT | 2014 － 2016 | 80/84 | positive/≥6.0 | <12/NR | 25.9 ± 3.09 vs 25.38 ± 2.9 | 7.63 ± 0.39 vs 7.55 ± 0.38 | NR | 91.3% (73/80) vs 0% (0/84) | 88.8% (71/80) vs 58.7% (37/84) | NR | NR |
| Shen GJ et al. (2021) | China | Non-RCT | 2015 － 2016 | 40/31 | NR/≥6.0 | 26-32/At delivery | 25.4 ± 3.4 vs 25.1 ± 3.0 | 7.34 ± 0.65 vs 7.21 ± 0.76 | 4.20 ± 0.57 vs 7.12 ± 0.43 | NR | 10% (4/40) vs 3.23% (1/31) | NR | NR |
| Zhang JM et al. (2021) | China | Non-RCT | 2018 － 2019 | 39/37 | NR/≥6.0 | 24/At delivery | 26.97 ± 4.8 vs 26.70 ± 4.81 | 7.71 ± 0.77 vs 7.67 ± 0.69 | NR | NR | NR | NR | NR |
| Liu JF et al.  (2019) | China | Non-RCT | 2010 － 2016 | 325/136 | NR/≥6.0 | 22-28/12 (postpartum) | 28.4 ± 4.4 vs 27.1 ± 4.7 | 7.68 ± 0.70 vs 7.71 ± 0.79 | NR | 100% (325/325) vs 0% (0/136) | 78.95% (75/95) vs 0% (0/136) | 4% (13/325) vs 0% (0/136) | NR |
| Zeng J et al. (2019) | China | Non-RCT | 2013 － 2017 | 51/36 | positive/≥7.0 | 22-28/NR | 26.5 ± 9.5 vs 25.7 ± 10.9 | 7.9 ± 0.8 vs 7.7 ± 0.5 | 1.6 ± 1.3 vs 7.69 ± 0.53 | NR | NR | NR | NR |
| Chang K et al. (2019) | China (Taiwan) | Non-RCT | 2011 － 2016 | 110/91 | positive/≥7.5 | 30-32/4 (postpartum) | 32.8 ± 3.6 vs 22 | 8.25 ± 0.48 vs 8.29 ± 0.40 | 4.29 ± 0.98 vs 8.12 ± 0.68 | 96.36% (106/110) vs 3.3% (3/91) | NR | NR | 2.73% (3/110) vs 0% (0/91) |
| Wang Y et al. (2019) | China | Non-RCT | 2014 － 2016 | 128/72 | positive/≥6.0 | 28/4 (postpartum) | 29.5 ± 3.8 vs 28.7 ± 4.2 | 7.87 ± 0.51 vs 7.83 ± 0.65 | 4.79 ± 0.89 vs 7.60 ± 0.73 | NR | NR | NR | NR |
| Mao C et al. (2019) | China | Non-RCT | 2016 － 2019 | 156/102 | NR/≥6.0 | 24/At delivery | 26.6 ± 3.5 vs 25.7 ± 3.9 | 5.68 ± 2.54 vs 5.45 ± 2.67 | 3.12 ± 2.21 vs 5.57 ± 2.42 | NR | NR | NR | NR |
| Ma L et al. (2019) | China | Non-RCT | 2015 － 2017 | 56/27 | NR/>7.0 | 28/At delivery | 25.4 ± 3.7 vs 26.1 ± 2.6 | 7.9 ± 1.0 vs 7.8 ± 0.8 | 3.9 ± 0.8 vs 7.6 ± 0.5 | NR | NR | NR | NR |
| Gao X et al. (2020) | China | Non-RCT | 2010 － 2018 | 81/63 | Both/≥5.0 | NR | 29.6 ± 2.8 vs 29.1 ± 3.4 | 6.3 ± 1.0 vs 6.4 ± 1.2 | 3.4 ± 0.5 vs 6.3 ± 1.5 | 95.1% (77/81) vs 4.8% (3/63) | NR | NR | NR |
| Ye Z et al. (2021) | China | Non-RCT | 2016 － 2020 | 26/26 | NR/≥6.0 | 24-28/At delivery | 28.3 ± 2.1 vs 29.0 ± 1.9 | 7.74 ± 0.52 vs 7.5 ± 0.5 | 3.75 ± 0.36 vs 7.2 ± 0.4 | NR | NR | NR | NR |
| Kuang C et al. (2021) | China | Non-RCT | 2017 － 2019 | 80/83 | positive/≥6.3 | 18/At delivery | 25.3 ± 1.6 vs 25.2 ± 1.6 | 7.54 ± 0.37 vs 7.47 ± 0.29 | NR | 91.25% (73/80) vs 0% (0/83) | 88.75％ (71/80) vs 43.37％ (36/83) | NR | NR |
| Cui D et al. (2021) | China | Non-RCT | 2017 － 2019 | 54/54 | NR/≥5.3 | 28/At delivery | 30.1 ± 4.4 vs 30.2 ± 4.4 | 7.57 ± 1.23 vs 7.59 ± 1.21 | NR | 83.33% (45/54) vs 3.7% (2/54) | NR | NR | NR |
| Ran R et al. (2021) | China | Non-RCT | 2016 － 2018 | 253/NR | NR/≥6.0 | 23-26/4 (postpartum) | 29 (27, 32) | 8.21 (8.04, 8.23) vs 8.10 (7.77, 8.23) no placebo | 4.41 (3.42, 4.89) vs 4.53 (3.29, 5.40) no placebo | NR | NR | NR | NR |
|  |  |  |  |  |  | 27-34/4 (postpartum) | 30 (28, 33) |  |  |  |  |  |  |
| Hu MF et al. (2018) | China | Non-RCT | 2016 － 2018 | 90/30 | NR/≥6.0 | Pre-pregnancy | 28.4 ± 1.4 | 7. 44 ± 0. 39 | 5. 84 ± 1. 35 | NR | NR | NR | NR |
|  |  |  |  |  |  | 14 | 23.2 ± 3.3 | 7. 50 ± 0. 47 | 5. 55 ± 1. 19 |  |  |  |  |
|  |  |  |  |  |  | 28 | 24.4 ± 3.1 | 7. 38 ± 0. 66 | 5. 67 ± 1. 25 |  |  |  |  |
| Wang HB et al. (2018) | China | Non-RCT | 2013 － 2016 | 100/20 | NR | 20 | NR | 7.0 | NR | NR | NR | NR | NR |
|  |  |  |  |  |  | 24 |  | 7.1 |  |  |  |  |  |
|  |  |  |  |  |  | 28 |  | 7.2 |  |  |  |  |  |
|  |  |  |  |  |  | 32 |  | 7.2 |  |  |  |  |  |
|  |  |  |  |  |  | 36 |  | 6.7 |  |  |  |  |  |
| Huang XL et al. (2023) | China | RCTs  [2020-21] | 2020 － 2021 | 44/44 | positive/≥5.3 | 24/At delivery | 28.46 ± 3.18 vs 28.15 ± 3.29 | 6.95 ± 0.96 vs 6.89 ± 0.85 | 3.21 ± 0.74 vs 6.53 ± 0.79 | NR | NR | NR | NR |
| **Maternal TAF therapy (25mg orally once a day) vs TDF therapy (300mg orally once a day)** | | | | | | | | | | | | | |
| Li B et al. (2021) | China | RCT | 2019 | 36/36 | positive/≥6.0 | 24/At delivery | 27.5 ± 4.1 vs 26.9 ± 3.9 | 7.9 ± 0.4 vs 7.9 ± 0.4 | 3.5 ± 0.6 vs 3.5 ± 0.6 | 100% (36/36) vs 100% (36/36) | NR | NR | NR |
| Zeng Q et al. (2021) | China | Non-RCT | 2019 | 116/116 | Both/≥5.0 | 24-35/At delivery | 29.6 ± 4.5 vs 29.3 ± 4.2 | 7.8 ± 0.7 vs 7.8 ± 0.7 | 3.5 ± 0.9 vs 3.4 ± 1.0 | 100% (116/116) vs 100% (116/116) | NR | 0% (0/116) vs 0% (0/116) | 7.76% (9/116) vs 7.76% (9/116) |
| Zeng Q et al. (2021) | China | Non-RCT | 2019 － 2020 | 103/104 | Both/≥5.0 | NR | 28.8 ± 4.5 vs NR | 5.1 ± 3.4 vs 4.6 ± 3.4 | 1.2 ± 1.7 vs 0.9 ± 1.4 | 100% (103/103) vs 100% (104/104) | 94.7% (97/103) vs 95.5% (99/104) | NR | 22% (23/103) vs 21.1% (22/104) |
| Pan SF et al. (2024)^&^ | China | Non-RCT | 2018 － 2021 | 25/35 | positive/≥5.3 | 20-23/NR | 30.4 ± 3.9 vs 29.6 ± 3.2 | 7.8 ± 0.7 vs 7.6 ± 0.8 | 3.5 (2.9, 4.0) vs 2.4 (2.0, 3.4) | NR | NR | NR | NR |

TDF: Tenofovir disoproxil fumarate; TAF: Tenofovir alafenamide; LdT: Telbivudine; MTCT: Mother-to-child transmission; ALT: Alanine aminotransferase; HBeAg: Hepatitis B e antigen; NR: Not reported;

^&^: This study compares the efficacy and safety of telbivudine (LdT), tenofoviralafenamide fumarate (TAF), and tenofovir disoproxil fumarate (TDF).

Table S3B. The characteristics of infants at birth and the efficacy outcomes of preventing HBV vertical transmission

| **Author**  **(Year)** | **Infants**  **Assessed in treatment/control groups,**  **N** | **Cesarean section in treatment/control groups,**  **N** | **The birth dose of HBIG**  **(dose, timing)** | **The birth dose of the Vaccine**  **(dose, timing)** | **Subsequent vaccine**  **(months)** | **Endpoint measurement of HBsAg**  **(months)** | **Endpoint measurement of HBV-DNA**  **(months)** | **HBsAg positive at birth, % (N)** | **Detectable HBV DNA at birth, % (N)** | **HBsAg positive at the age of 6-12 months, % (N)** | **Detectable HBV DNA at the age of 6-12 months, % (N)** | **Criteria for MTCT** |
| --- | --- | --- | --- | --- | --- | --- | --- | --- | --- | --- | --- | --- |
| **Maternal TDF therapy (300mg orally once a day) vs Placebo** | | | | | | | | | | | | |
| Chen H et al. (2015) | 65/56 | 27/17 | 100IU, <24hr | 5ug, <24hr | 1, 6 | 0, 6, 12 | 0, 6, 12 | 10.8% (7/65) vs 17.8% (10/56) | 6.2% (4/65) vs 31.5% (17/56) | 1.5% (1/65) vs 10.7% (6/56) | NR | HBsAg positivity at 6 months |
| Pan C et al. (2016) | 95/88 | 47/50 | 200IU, <12hr | 10ug, <12hr | 1, 6 | 0, 7 | 0, 7 | 6.2% (6/97) vs 4.0% (4/100) | 3.1% (3/97) vs 15.0% (15/100) | 5.2% (5/97) vs 18.0% (18/100) | NR | HBV DNA>20IU/mL or HBsAg positivity at 7 months |
| Samadi K et al. (2016) | 24/146 | 8/36 | NR, at birth | NR, at birth | 2, 6 | 0, 7 | 0, 7 | NR | NR | 0% (0/12) vs 1.3% (1/73) | NR | HBsAg positivity at 7 months |
| Liu M et al. (2017) | 20/20 | 7/5 | 200IU, <24hr | 10ug, <24h | 1, 6 | 7 | 7 | NR | NR | 5% (1/20) vs 30% (6/20) | 5% (1/20) vs 20% (4/20) | HBsAg positivity at 7 months |
| Chen W et al. (2017) | 30/44 | NR | 100IU, at birth | 10ug, at birth | 1, 6 | 0, 7, 12 | 0, 7, 12 | 3% (1/30) vs 40.9% (18/44) | 3% (1/30) vs 34.1% (15/44) | 3% (1/30) vs 34.1% (15/44) | 0% (0/30) vs 25% (11/44) | HBV DNA and HBsAg positivity at 7 months and 12 months |
| Jourdain G et al. (2018) | 147/147 | 38/47 | 200IU, <3hr | 10ug, <3hr | 1, 2, 4, 6 | NR | 6 | 0% (0/147) vs 1% (2/147) | 0% (0/147) vs 1% (2/147) | 0% (0/147) vs 2% (3/147) | 0% (0/147) vs 2% (3/147) | HBV DNA and HBsAg positivity at 6 months |
| Lin Y et al. (2018) | 58/52 | 21/17 | NR, <24hr | NR, <12hr | 1, 6 | 7 | 7 | NR | NR | 0% (0/58) vs 7.7% (4/52) | 0% (0/58) vs 13.5% (7/52) | HBV DNA or HBsAg positivity at 7 months |
| Greenup AJ et al. (2014) | 58/20 | 0/0 | NR, at birth | NR, at birth | 2, 4, 6 | 9 | NR | NR | 0.02% (1/44) vs 20% (2/10) | NR | 0.02% (1/44) vs 20% (2/10) | HBsAg positivity at 9 months |
| Celen MK et al. (2013) | 21/23 | NR | 200IU, <24hr | 20ug, <24hr | 4,8,24 | 7 | 7 | 0% (0/21) vs 8.3% (2/23) | 0% (0/21) vs 8.3% (2/23) | 0% (0/21) vs 8.3% (2/23) | 0% (0/21) vs 8.3% (2/23) | HBV DNA and HBsAg positivity at 7 months |
| Wang YC et al. (2020) | 72/56 | NR | NR, <24hr | NR, <72hr | 1,6 | 0 | 0 | NR | NR | 0% (0/72) vs 17.9% (10/56) | 0% (0/72) vs 14.3% (8/56) | HBsAg positivity at delivery |
| Chen CY et al. (2019) | 80/84 | NR | NR, at birth | NR, at birth | NR | 0, 6, 12 | 0, 6, 12 | NR | NR | 0% (0/80) vs 8.3% (7/84) | 0% (0/80) vs 8.3% (7/84) | HBV DNA and HBsAg positivity at 6 months and 12 months |
| Shen GJ et al. (2021) | 40/31 | NR | 100IU, <12hr | 10ug, <12hr | 1,6 | 7 | 7 | NR | NR | 0% (0/39) vs 10.8% (4/37） | NR | HBV DNA>20IU/mL or HBsAg positivity at 7 months |
| Zhang JM et al. (2021) | 39/37 | NR | 100IU, <12hr | 10ug, <12hr | 1,6 | 7 | 7 | NR | NR | 4.02% (1/325) vs 5% (7/136) | NR | HBsAg positivity at 7 months |
| Liu JF et al.(2019) | 325/136 | 162/67 | 200IU, <24hr | 10ug, <24hr | 1,6 | 7 | 7 | NR | NR | 0% (0/51) vs 11.1% (4/36) | NR | HBV DNA>20IU/mL or HBsAg positivity at 7 months |
| Zeng J et al. (2019) | 51/36 | 20/11 | 200IU, <6hr | 20ug, <12hr | 1,6 | 6, 12 | 6, 12 | 9.56% (11/115) vs 16.13% (15/93) | 5.22% (6/115) vs30.11% (28/93) | 1.74% (2/115) vs 11.83% (11/93) | NR | HBsAg positivity at 6 months and 12 months |
| Chang K et al. (2019) | 115/93 | 36/27 | 100IU, <24hr | NR, <24hr | 1,6 | 0, 6, 12 | 0, 6, 12 | NR | NR | 0%（0/128）vs 5.6%（4/72） | 0%（0/128）vs 5.6%（4/72） | HBV DNA and HBsAg positivity at 6 months and 12 months |
| Wang Y et al. (2019) | 128/72 | NR | 100IU, <6hr | 20ug, <6hr | 1,6 | 7 | 7 | NR | NR | 1.28% (2/156) vs 8.82% (9/102) | 0% (0/156) vs 5.88% (6/102) | HBV DNA and HBsAg positivity at 7 months |
| Mao C et al. (2019) | 156/102 | 38/26 | 200IU, <12hr | 10ug, <12hr | 1,6 | 7 | 7 | NR | NR | 0% (0/56) vs 0% (0/56) | 11.1 % (3/27) vs 81.5% (22/27) | HBV DNA or HBsAg positivity at 7 months |
| Ma L et al. (2019) | 56/27 | NR | 100IU, NR | 10ug, NR | 1,6 | 12 | 12 | 18.5% (15/81) vs 36.5% (23/63) | 0% (0/81) vs 7.9% (5/63) | 0% (0/81) vs 6.3% (4/63) | 0% (0/81) vs 6.3% (4/63) | HBV DNA or HBsAg positivity at 12 months |
| Gao X et al. (2020) | 81/63 | 33/29 | 200IU, <2hr | 10ug, <2hr | 1,6 | 0, 7 | 0, 7 | NR | NR | 0% (0/26) vs 15.38% (4/26) | 0% (0/26) vs 15.38% (4/26) | HBV DNA and HBsAg positivity at 7 months |
| Ye Z et al. (2021) | 26/26 | NR | 100IU, <24hr | 10ug, <24hr | 1,6 | 7 | 7 | 0% (0/80) vs 9.64% (8/83) | 0% (0/80) vs 8.43% (7/83) | 0% (0/80) vs 8.43% (7/83) | 0% (0/80) vs 8.43% (7/83) | HBV DNA or HBsAg positivity at 7 months |
| Kuang C et al. (2021) | 80/83 | NR | NR | NR | NR | 0, 6, 12 | 0, 6, 12 | NR | NR | 0% (0/40) vs 9.7% (3/31) | 0% (0/40) vs 9.7% (3/31) | HBV DNA and HBsAg positivity at 6 months and 12 months |
| Cui D et al. (2021) | 54/54 | NR | 100IU, <24hr | 10ug, <24hr | 1,6 | 7 | 7 | NR | NR | 3.7% (2/54) vs 14.81% (8/54) | 0% (0/54) vs 12.96% (7/54) | HBV DNA and HBsAg positivity at 7 months |
| Ran R et al. (2021) | 253/NR | 103/NR | 200IU, <2hr | 10ug, <2hr | 1,6 | 0, 7 | 0, 7 | 13.9% (15/108) vs 10.9% (15/137) | 0% (0/108) vs 0% (0/137) | 0% (0/108) vs 0% (0/137) | 0% (0/108) vs 0% (0/137) | HBsAg positivity at 7 months |
| Hu MF et al. (2018) | 90/30 | NR | 200IU, <2hr | 10ug, <2hr | 1,6 | 0, 7 | 0, 7 | NR | NR | NR | NR | HBV DNA or HBsAg positivity at 7 months |
| Wang HB et al. (2018) | 100/20 | NR | 200IU, <2hr | 10ug, <2hr | 1,6 | 0, 7, 12 | 0, 7, 12 | NR | NR | NR | NR | HBV DNA and HBsAg positivity at 12 months |
| Huang XL et al. (2023) | 44/44 | 23/20 | 100IU, <12hr | 10ug, <12hr | 1,6 | 0, 8 | 0, 8 | 2.3% (1/44) vs 18.2% (8/44) | 0% (0/44) vs 15.9% (7/44) | 0% (0/44) vs 15.9% (7/44) | 0% (0/44) vs 13.6% (6/44) | HBV DNA and HBsAg positivity at 8 months |
| **Maternal TAF therapy (25mg orally once a day) vs TDF therapy (300mg orally once a day)** | | | | | | | | | | | | |
| Li B et al. (2021) | 36/36 | NR | 100IU, <12hr | 10ug, <12hr | 1,6 | 0, 6 | 0, 6 | 0% (0/36) vs 0% (0/36) | 0% (0/36) vs 0% (0/36) | 0% (0/36) vs 0% (0/36) | 0% (0/36) vs 0% (0/36) | HBV DNA and HBsAg positivity at 6 months |
| Zeng Q et al. (2021) | 117/116 | 56/60 | NR, <12hr | NR, <12hr | 1,6 | 7 | 7 | NR | NR | 0% (0/116) vs 0% (0/116) | 0% (0/116) vs 0% (0/116) | HBsAg positivity at 7 months |
| Zeng Q et al. (2021) | 102/104 | 35/39 | 100IU, <12hr | 10ug, <12hr | 1,6 | 7 | 7 | 0% (0/102) vs 0% (0/104) | 0% (0/102) vs 0% (0/104) | 0% (0/102) vs 0% (0/104) | 0% (0/102) vs 0% (0/104) | HBsAg positivity at 7 months |
| Pan SF et al. (2024)^&^ | 25/35 | NR | 100IU, <24hr | 10ug, <24hr | 1,6 | 0, 7 | 0, 7 | 4.0% (1/25) vs 5.7% (2/35) | 0% (0/25) vs 0% (0/35) | 0% (0/25) vs 0% (0/35) | 0% (0/25) vs 0% (0/35) | HBV DNA or HBsAg positivity at 7 months |

TDF: Tenofovir disoproxil fumarate; TAF: Tenofovir alafenamide; LdT: Telbivudine; MTCT: Mother-to-child transmission; ALT: Alanine aminotransferase; HBeAg: Hepatitis B e antigen; HBIG: Hepatitis B immune globulin; BD: Birth dose; NR: Not reported.

^&^: This study compares the efficacy and safety of telbivudine (LdT), tenofoviralafenamide fumarate (TAF), and tenofovir disoproxil fumarate (TDF).

**Supplementary** **Appendix 6.** **Assessment of between-study heterogeneity and the summary effect influenced by a particular study**

Supplementary Figure S1. Sensitivity analysis in all TDF studies by omitting each trial one by one


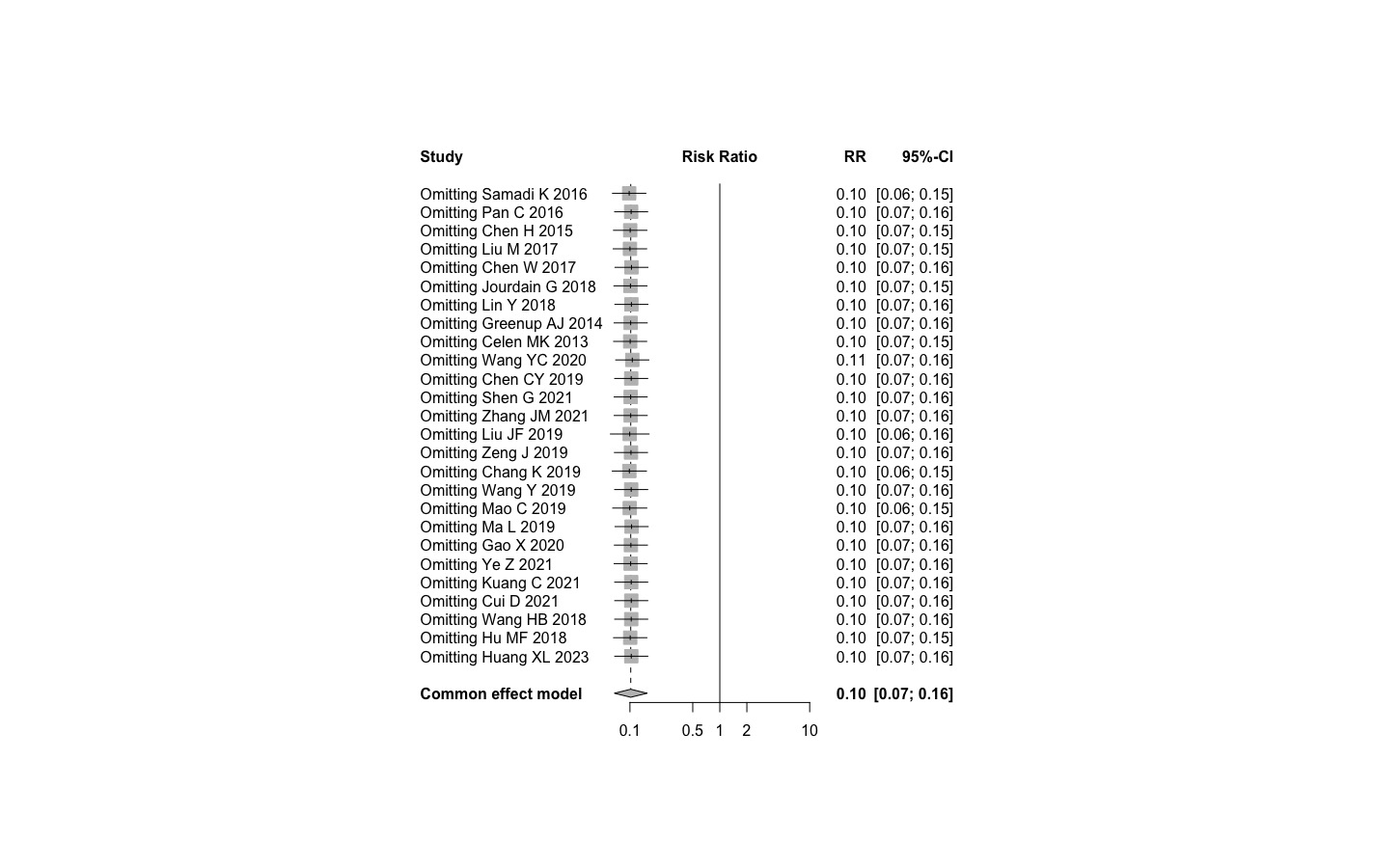


**Supplementary Appendix 7.** **The efficacy rank of TAF vs TAF on preventing MTCT estimated by the network met-analysis**

Supplementary Figure S2A. Forest plot for the overall efficacy of TDF vs TDF on preventing MTCT in network meta-analysis

TAF vs TDF: RR 1.09 (95% CI: 0.15 – 7.65), p-value = 0.99


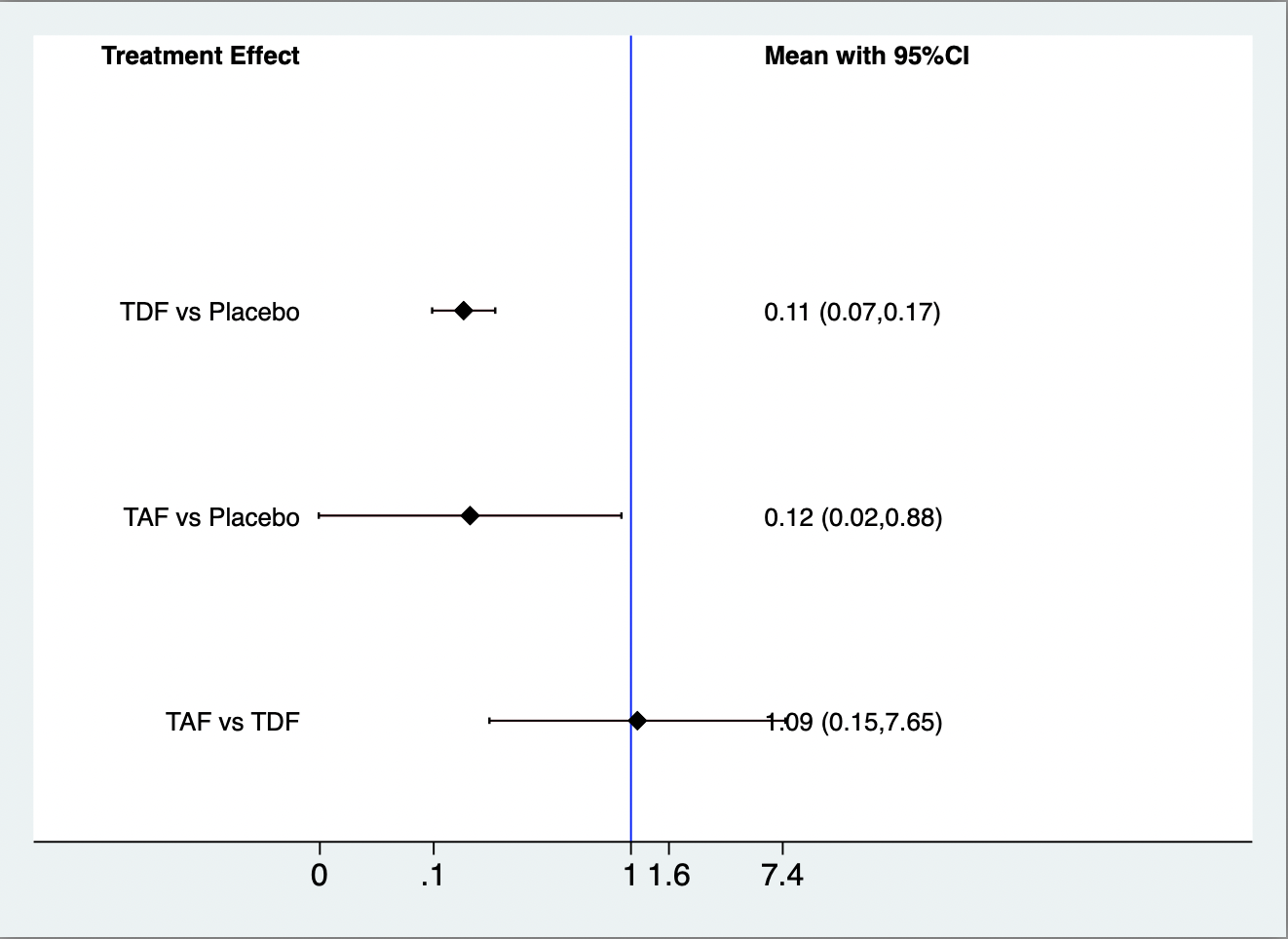


TDF: Tenofovir disoproxil fumarate; TAF: Tenofovir alafenamide; MTCT: Mother-to-child transmission.

Supplementary Figure S2B. The surface under the cumulative ranking curves for the overall efficacy of TDF vs inF on preventing MTCT


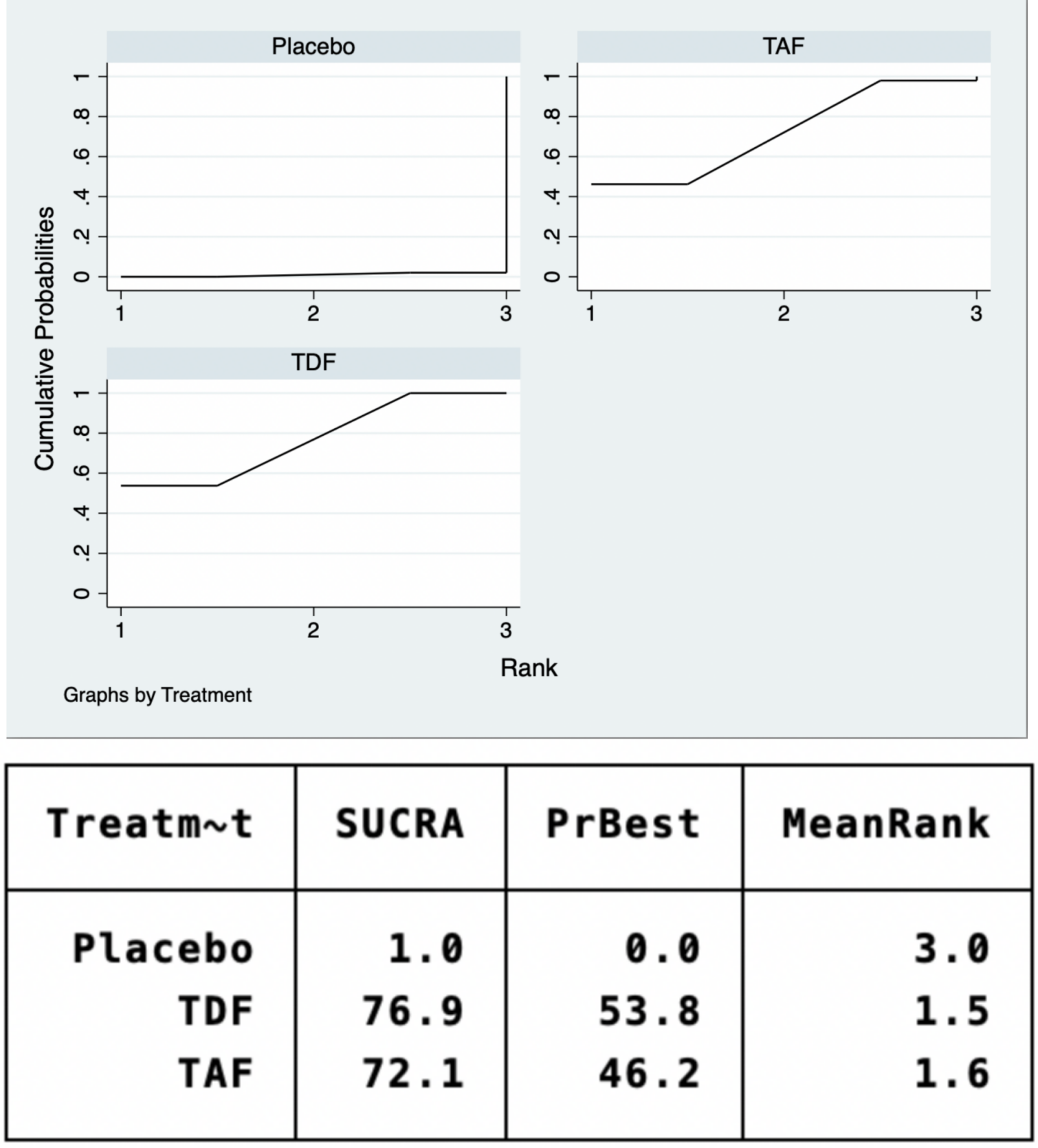


**Supplementary Appendix 8.** **Efficacy of antiviral prophylaxis using TDF or TAF by sub-group analyses**

Supplementary Figure S3A. Efficacy of TDF in the prevention of MTCT with different levels of HBV-DNA at baseline of mothers


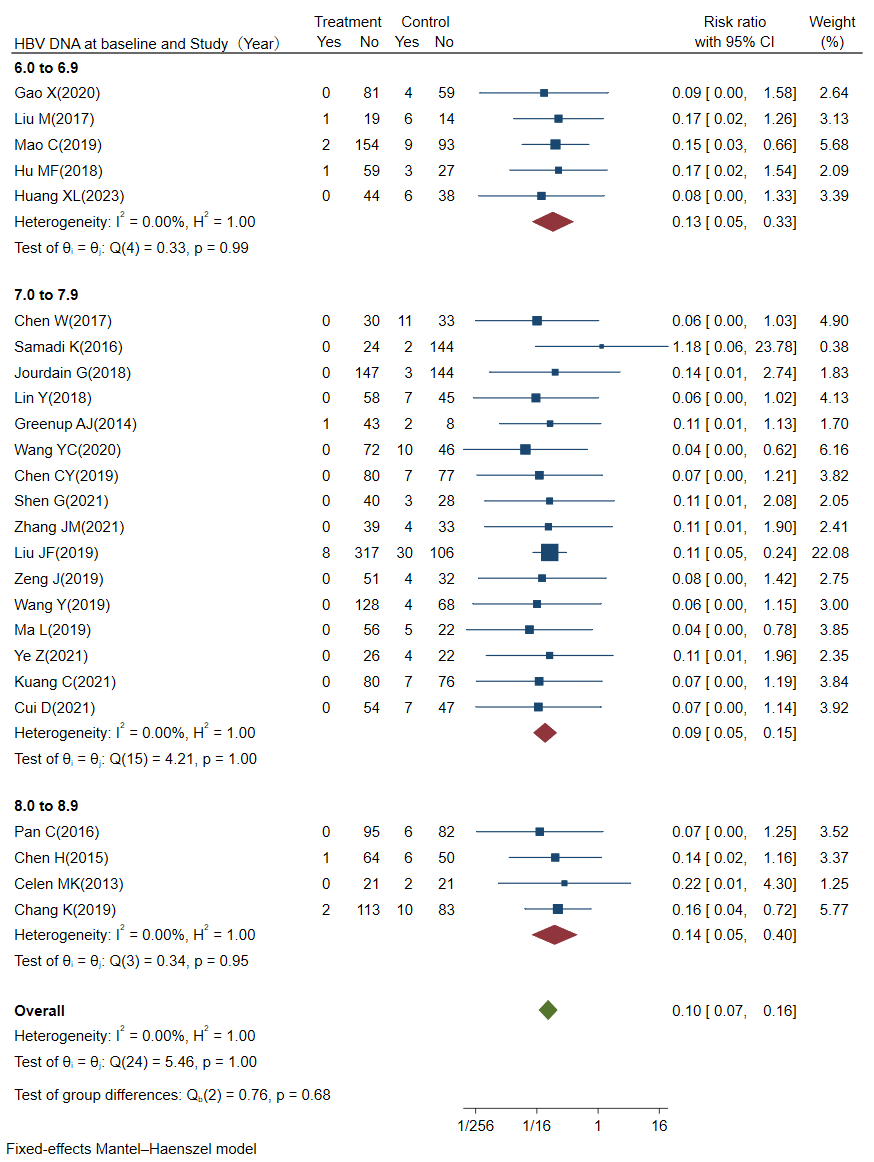


TDF: tenofovir disoproxil fumarate; MTCT: mother-to-child transmission;

Yes/No: events numbers/no events numbers; 95% CI: 95% confidence interval.

Supplementary Figure S3B. Efficacy of TDF in the prevention of MTCT with different statuses of HBeAg of mothers.


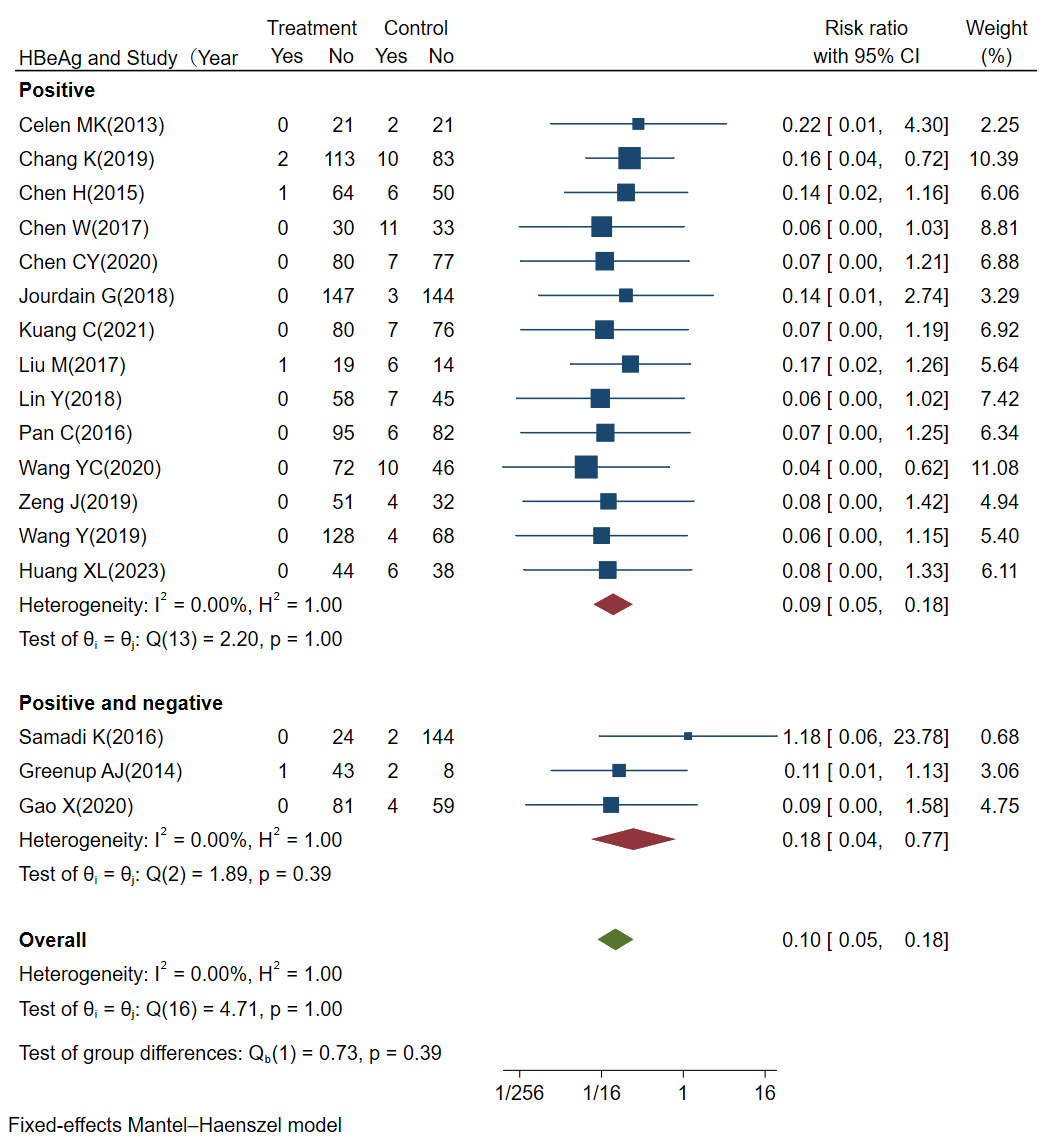


TDF: tenofovir disoproxil fumarate; MTCT: mother-to-child transmission; HBeAg: hepatitis B e antigen.

Yes/No: events numbers/no events numbers; 95% CI: 95% confidence interval.

Supplementary Figure S3C. Efficacy of TDF in the prevention of MTCT with different Languages.


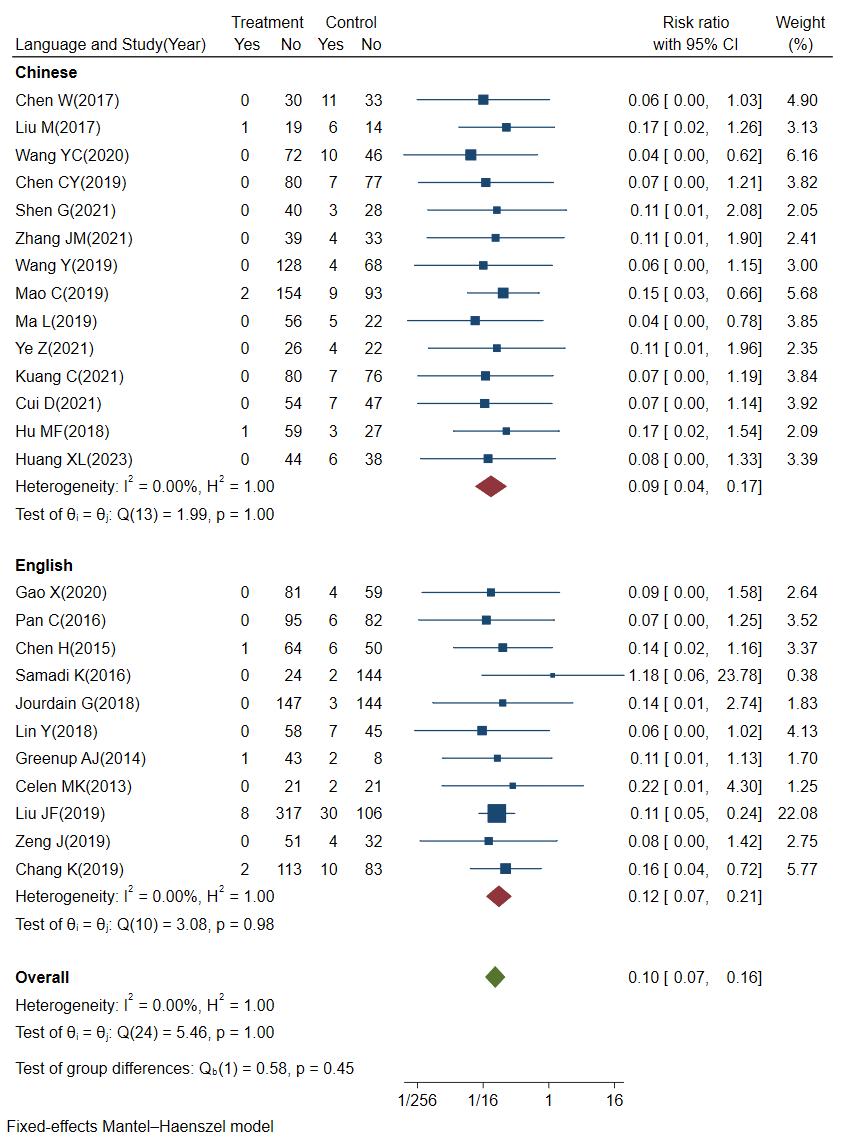


TDF: tenofovir disoproxil fumarate; MTCT: mother-to-child transmission;

Yes/No: events numbers/no events numbers; 95% CI: 95% confidence interval.

Supplementary Figure S3D. Efficacy of TDF in the prevention of MTCT with different timing of administrating birth doses of the HBV vaccine


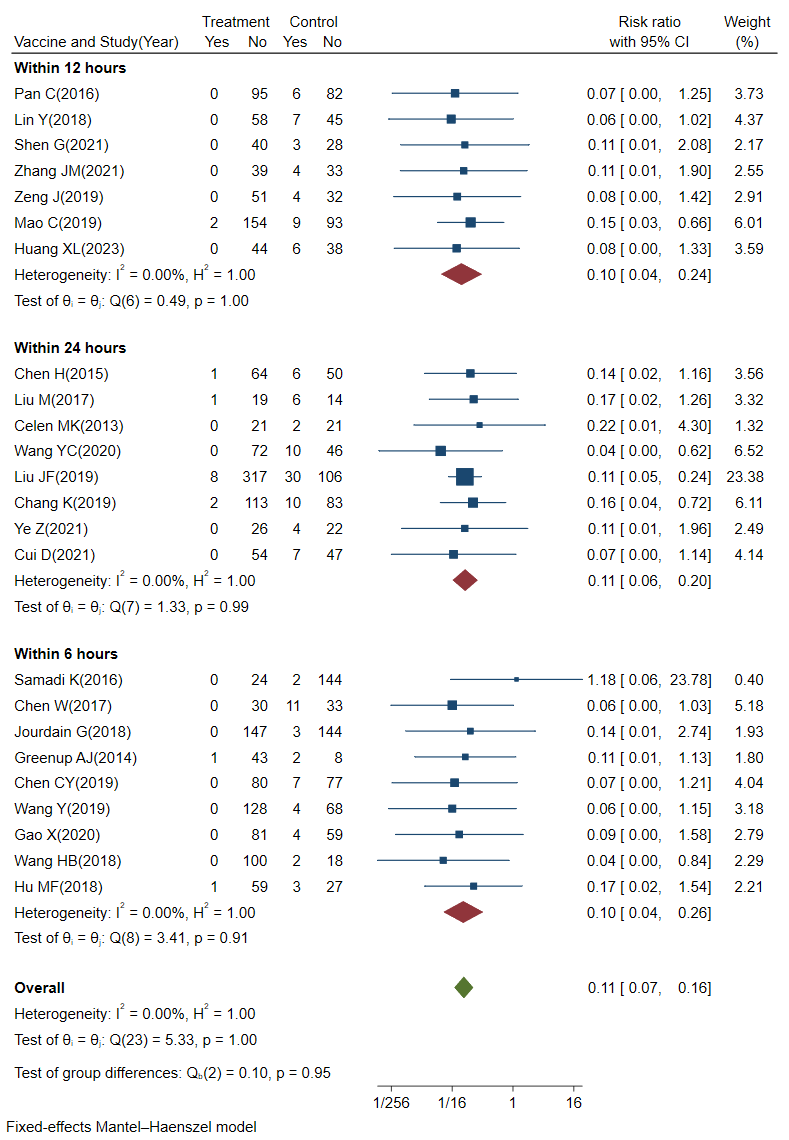


TDF: tenofovir disoproxil fumarate; MTCT: mother-to-child transmission;

Yes/No: events numbers/no events numbers; 95% CI: 95% confidence interval.

Supplementary Figure S3E. Efficacy of TDF in the prevention of MTCT with different timing of administrating birth doses of HBIg


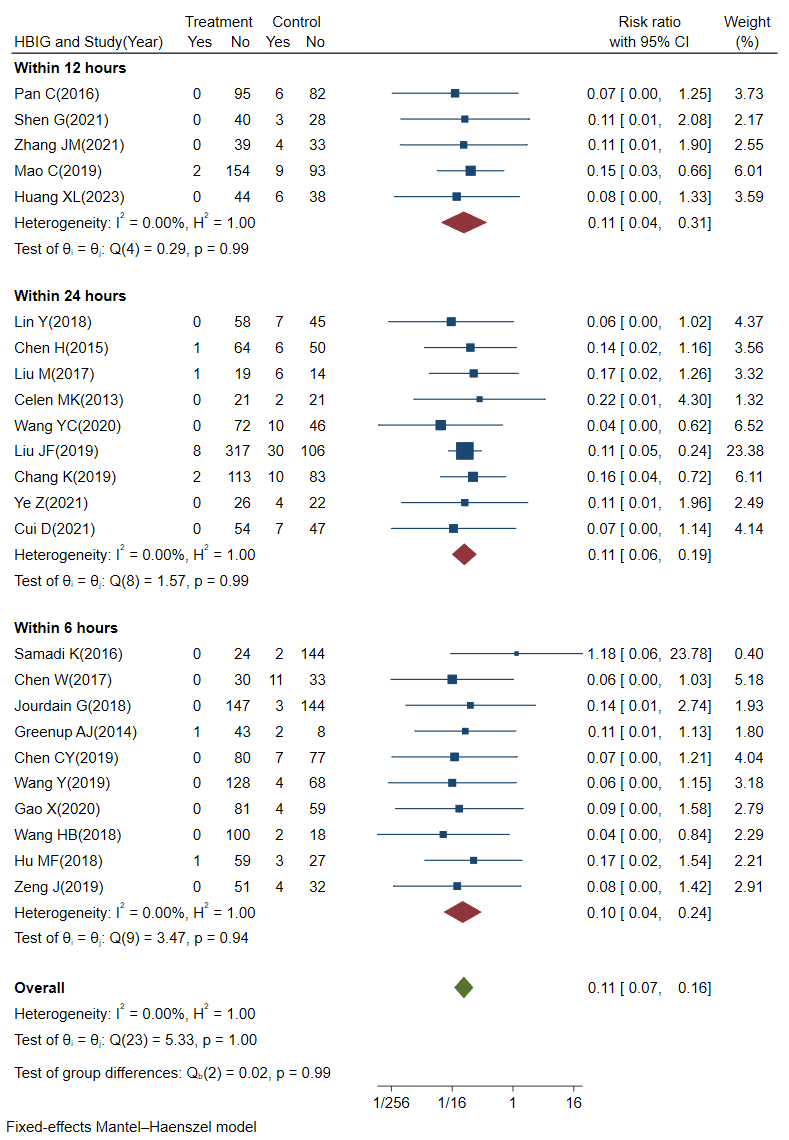


TDF: tenofovir disoproxil fumarate; MTCT: mother-to-child transmission; HBIg: hepatitis B immune globulin;

Yes/No: events numbers/no events numbers; 95% CI: 95% confidence interval.

**Supplementary Appendix 9. Safety of peripartum antiviral prophylaxis using TDF or TAF**

| **Author**  **(Year)** | **Maternal safety**  **（Antiviral agents vs Control）** | | | | | | **Infants’ safety with follow-up in one year**  **（Antiviral agents vs Control）** | | | | | | | **Infants’ safety with follow-up 2-5 years**  **（Antiviral agents vs Control）** | | | |
| --- | --- | --- | --- | --- | --- | --- | --- | --- | --- | --- | --- | --- | --- | --- | --- | --- | --- |
|  | **Fetal death, % (N)** | **Pregnancy Complications, % (N)** | **Post-partum hemorrhage, % (N)** | **Post-partum ALT flare, % (N)** | **CK elevation, % (N)** | **Grade III/IV AE, % (N)** | **Prematurity rate, % (N)** | **Congenital malformation/defect rate, % (N)** | **Apgar score (1 minute) <10, % (N)** | **Physical growth, % (N)** | **Bone density, % (N)** | **Neuro-mental development parameters, % (N)** | **Grade III/IV AE, % (N)** | **Physical growth, % (N)** | **Bone density, % (N)** | **Grade III/IV AE, % (N)** | **Neuro-mental development parameters, % (N)** |
| **Maternal TDF therapy (300mg orally once a day) vs Placebo** | | | | | | | | | | | | | | | | | |
| Chen H et al. (2015) | 0% (0/62) vs 0% (0/56) | NR | NR | 1.6% (1/62) vs 14.3% (8/56) | NR | NR | NR | 0% (0/65) vs 0% (0/56) | 0% (0/65) vs 0% (0/56) | 0% (0/65) vs 0% (0/56) | NR | NR | NR | NR | NR | NR | NR |
| Pan C et al. (2016) | 2.1% (2/97) vs 0% (0/100) | 23.71% (23/97) vs 13% (13/100) | 4.12% (4/97) vs 4% (4/100) | 6.19% (6/97) vs 9% (9/100) | 7.22% (7/97) vs 0% (0/100) | 1.03% (1/97) vs 1% (1/100) | 2.11% (2/95) vs 1.14% (1/88) | 2.11% (2/95) vs 1.14% (1/88) | 0% (0/95) vs 0% (0/88) | 0% (0/95) vs 0% (0/88) | NR | NR | 3.16% (3/95) vs 1.14% (1/88) | NR | NR | NR | NR |
| Samadi K et al. (2016) | 0% (0/23) vs 0.7% (1/138) | NR | NR | 17.39% (4/23) vs NR | NR | NR | 8.33% (2/24) vs 2.05% (3/146) | 0% (0/24) vs NR | NR | NR | NR | NR | NR | NR | NR | NR | NR |
| Liu M et al. (2017) | 0% (0/20) vs 0% (0/20) | 0% (0/20) vs 0% (0/20) | 0% (0/20) vs 0% (0/20) | NR | NR | NR | 0% (0/20) vs 0% (0/20) | 0% (0/20) vs 0% (0/20) | 0% (0/20) vs 0% (0/20) | 0% (0/20) vs 0% (0/20) | NR | NR | NR | NR | NR | NR | NR |
| Chen W et al. (2017) | 0% (0/30) vs 0% (0/44) | 0% (0/30) vs 0% (0/44) | 0% (0/30) vs 0% (0/44) | NR | NR | NR | 0% (0/30) vs 0% (0/44) | 0% (0/30) vs 0% (0/44) | 0% (0/30) vs 0% (0/44) | 0% (0/30) vs 0% (0/44) | NR | NR | NR | NR | NR | NR | NR |
| Jourdain G et al. (2018) | 0.6% (1/168) vs 0% (0/163) | 4% (6/168) vs 8%(13/163) | NR | 5.8% (9/154) vs 3.2% (5/157) | NR | 24.4% (41/168) vs 27.00% (44/163) | 0% (0/161) vs 0.6% (1/160) | NR | NR | 0% (0/161) vs 0% (0/160) | NR | NR | 26.71% (43/161) vs 23.75% (38/160) | NR | NR | NR | NR |
| Lin Y et al. (2018) | 0% (0/59) vs 0% (0/52) | 10.3% (6/59) vs 9.6% (5/52) | 0% (0/59) vs 0% (0/52) | 3.45% (2/59) vs 0% (0/52) | NR | NR | 1.69% (1/59) vs 3.85% （2/52） | 0% (0/59) vs 0% (0/52) | 0% (0/59) vs 0% (0/52) | 0% (0/59) vs 0% (0/52) | NR | NR | NR | NR | NR | NR | NR |
| Greenup AJ et al. (2014) | 0% (0/58) vs 0% (0/20) | 15.52% (9/58) vs NR | 9% (5/58) vs 15% (3/20) | 50% (22/44) vs 28.57% (4/14) | NR | NR | 1.72% (1/58) vs 0% (0/20) | 0% (0/58) vs 0% (0/20) | 0% (0/58) vs 0% (0/20) | 0% (0/58) vs 0% (0/20) | NR | NR | NR | NR | NR | NR | NR |
| Celen MK et al. (2013) | 0% (0/21) vs 4.17% (1/24) | 28.57% (6/21) vs 4.17% (1/24) | NR | 0% (0/21) vs NR | 4.76% (1/21) vs 0% (0/24) | NR | NR | 0% (0/21) vs 0% (0/23) | NR | 0% (0/21) vs 0% (0/23) | NR | NR | NR | NR | NR | NR | NR |
| Wang YC et.al. (2020) | 0% (0/72) vs 0% (0/56) | NR | NR | NR | NR | NR | 0% (0/72) vs 0% (0/56) | 0% (0/72) vs 0% (0/56) | NR | 0% (0/72) vs 0% (0/56) | 0% (0/72) vs 0% (0/56) | NR | NR | NR | NR | NR | NR |
| Chen CY et al. (2020) | 0% (0/80) vs 0% (0/84) | 0% (0/80) vs 0% (0/84) | 0% (0/80) vs 0% (0/84) | 0% (0/80) vs 0% (0/84) | NR | 0% (0/80) vs 0% (0/84) | 0% (0/80) vs 0% (0/84) | 0% (0/80) vs 0% (0/84) | 0% (0/80) vs 0% (0/84) | 0% (0/80) vs 0% (0/84) | 0% (0/80) vs 0% (0/84) | NR | NR | NR | NR | NR | NR |
| Shen GJ et al. (2021) | 0% (0/40) vs 0% (0/31) | 0% (0/40) vs 0% (0/31) | 0% (0/40) vs 0% (0/31) | 0% (0/40) vs 0% (0/31) | 0% (0/40) vs 0% (0/31) | 0% (0/40) vs 0% (0/31) | 0% (0/40) vs 0% (0/31) | 0% (0/40) vs 0% (0/31) | 0% (0/40) vs 0% (0/31) | 0% (0/40) vs 0% (0/31) | NR | NR | NR | NR | NR | NR | NR |
| Zhang JM et al. (2012) | 0% (0/39) vs 0% (0/37) | NR | NR | NR | NR | NR | NR | 0% (0/39) vs 0% (0/37) | 0% (0/39) vs 0% (0/37) | 0% (0/39) vs 0% (0/37) | NR | NR | NR | NR | NR | NR | NR |
| Liu JF et al. (2019) | 0% (0/325) vs 0% (0/136) | 73.54% (239/325) vs 52.21% (71/136) | 0% (0/325) vs 0.74% (1/136) | 29.23% (95/325) vs 24.27% (33/136) | 0% (0/325) vs 0% (0/136) | 0% (0/325) vs 0% (0/136) | 4.26% (10/325) vs 1.47% (2/136) | 0.31% (1/325) vs 0% (0/136) | 2.46% (8/325) vs 0.74% (1/136) | 0% (0/325) vs 0% (0/136) | NR | NR | NR | NR | NR | NR | NR |
| Zeng J et al. (2019) | NR | 7.1% (5/51) vs 6.5% (3/36) | 11.3% (8/51) vs 10.9% (5/36) | NR | NR | NR | NR | NR | 0% (0/51) vs 0% (0/36) | 0% (0/51) vs 0% (0/36) | NR | NR | NR | NR | NR | NR | NR |
| Chang K et al. (2019) | NR | NR | NR | NR | NR | NR | NR | NR | NR | NR | NR | NR | NR | NR | NR | NR | NR |
| Wang Y et al. (2019) | 0% (0/128) vs 0% (0/72) | NR | NR | NR | NR | NR | NR | NR | NR | 0% (0/128) vs 0% (0/72) | 0% (0/128) vs 0% (0/72) | NR | NR | NR | NR | NR | NR |
| Mao C et al. (2019) | NR | 2.6% (4/156) vs 0% (0/102) | NR | NR | NR | 0% (0/156) vs 0% (0/102) | NR | 0% (0/156) vs 0% (0/102) | NR | 0% (0/156) vs 0% (0/102) | NR | NR | NR | NR | NR | NR | NR |
| Ma L et al. (2019) | NR | 7.1% (4/56) vs 0% (0/27) | NR | NR | NR | NR | NR | NR | 0% (0/56) vs 0% (0/27) | 0% (0/56) vs 0% (0/27) | 0% (0/56) vs 0% (0/27) | NR | NR | NR | NR | NR | NR |
| Gao X et al. (2020) | 0% (0/81) vs 0% (0/63) | 34.57% (28/81) vs 65.08% (41/63) | 3.70% (3/81) vs 4.76% (3/63) | NR | 0% (0/81) vs 0% (0/63) | 0% (0/81) vs 0% (0/63) | 3.70% (3/81) vs 6.35% (4/63) | 3.70% (3/81) vs 3.17% (2/63) | 0% (0/81) vs 0% (0/63) | 0% (0/81) vs 0% (0/63) | NR | NR | NR | NR | NR | NR | NR |
| Ye Z et al. (2021) | 0% (0/26) vs 0% (0/26) | 7.69% (2/26) vs 0% (0/26) | NR | NR | NR | NR | NR | NR | NR | NR | NR | NR | NR | NR | NR | NR | NR |
| Kuang C et al. (2021) | 0% (0/80) vs 0% (0/83) | NR | NR | NR | NR | NR | NR | NR | NR | NR | NR | NR | NR | NR | NR | NR | NR |
| Cui D et al. (2021) | 0% (0/54) vs 0% (0/54) | 5.56% (3/54) vs NR | NR | NR | NR | NR | NR | NR | NR | NR | NR | NR | NR | NR | NR | NR | NR |
| Ran R et al. (2021) | NR | NR | NR | NR | NR | NR | 0% (0/108) vs 0% (0/137) | 0% (0/108) vs 0% (0/137) | NR | 0% (0/108) vs 0% (0/137) | NR | NR | NR | NR | NR | NR | NR |
| Hu MF et al. (2018) | NR | 3.33% (3/90) | NR | NR | NR | NR | NR | NR | NR | NR | NR | NR | NR | NR | NR | NR | NR |
| Wang HB et al. (2018) | NR | NR | NR | NR | NR | NR | NR | NR | NR | NR | NR | NR | NR | NR | NR | NR | NR |
| Wen WH et al. (2020) | NR | NR | NR | NR | NR | NR | NR | NR | NR | NR | NR | NR | NR | 0% (0/71) vs 0% (0/57) | 0% (0/71) vs 0% (0/53) | NR | NR |
| Salvadori N et al. (2019) | NR | NR | NR | NR | NR | NR | NR | NR | NR | NR | 0% (0/62) vs 0% (0/53) | NR | NR | NR | NR | NR | NR |
| Pan C et al. (2022) | NR | NR | NR | NR | NR | NR | 2.11% (2/95) vs 1.14% (1/88) | NR | NR | NR | NR | NR | NR | 0% (0/70) vs 0% (0/75) | 0% (0/70) vs 0% (0/75) | 6.74% (6/89) vs 9.30% (8/86) | 0% (0/70) vs 0% (0/75) |
| Huang XL et al. (2023) | NR | 6.82% (3/44) vs 0.00% (0/44) | 13.64% (6/44) vs 18.18% (8/44) | NR | NR | NR | 4.55% (2/44) vs 2.27% (1/44) | NR | NR | 0% (0/44) vs 0% (0/44) | NR | NR | NR | NR | NR | NR | NR |
| **Maternal TAF therapy (25mg orally once a day) vs TDF therapy (300mg orally once a day)** | | | | | | | | | | | | | | | | | |
| Li B et al. (2021) | 0% (0/36) vs 0% (0/36) | NR | NR | 0 vs 0 | NR | NR | NR | 0% (0/36) vs 0% (0/36) | 0% (0/36) vs 0% (0/36) | 0% (0/36) vs 0% (0/36) | NR | NR | NR | NR | NR | NR | NR |
| Zeng Q et al. (2021) | 0% (0/116) vs 0% (0/116) | 19.83% (23/116) vs 18.10% (21/116) | 0% (0/116) vs 0% (0/116) | 0% (0/116) vs 0% (0/116) | NR | NR | 3.4% (4/117) vs 3.4% (4/116) | 0% (0/117) vs 0% (0/116) | 0% (0/117) vs 0% (0/116) | 0% (0/117) vs 0% (0/116) | NR | 0% (0/117) vs 0% (0/116) | NR | NR | NR | NR | NR |
| Zeng Q et al. (2021) | NR | 24.27% (25/103) vs 29.81% (31/104) | 0.97% (1/103) vs 1.92% (2/104) | NR | NR | NR | 2.91% (3/103) vs 3.85% (4/104) | 0.97% (1/103) vs 0% (0/104) | 0% (0/103) vs 0% (0/104) | 0% (0/103) vs 0% (0/104) | NR | NR | NR | NR | 0% (0/103) vs 0% (0/104) | NR | NR |
| Pan SF et al. (2024)^&^ | NR | NR | NR | NR | NR | NR | NR | 12% (3/25) vs 5.7% (2/35) | NR | 0% (0/25) vs 2.9% (1/35) | 0% (0/25) vs 0% (0/35) | 0% (0/25) vs 0% (0/35) | NR | NR | NR | NR | NR |

TDF: Tenofovir disoproxil fumarate; TAF: Tenofovir alafenamide; MTCT: Mother-to-child transmission; ALT: Alanine aminotransferase; CK: Creatine kinase; AE: Advanced event; NR: Not reported.

**Supplementary Appendix 10.** **Fetal safety of peripartum antiviral prophylaxis using TDF or TAF**

Supplementary Figure S4A. Fetal safety of TDF in fetal death


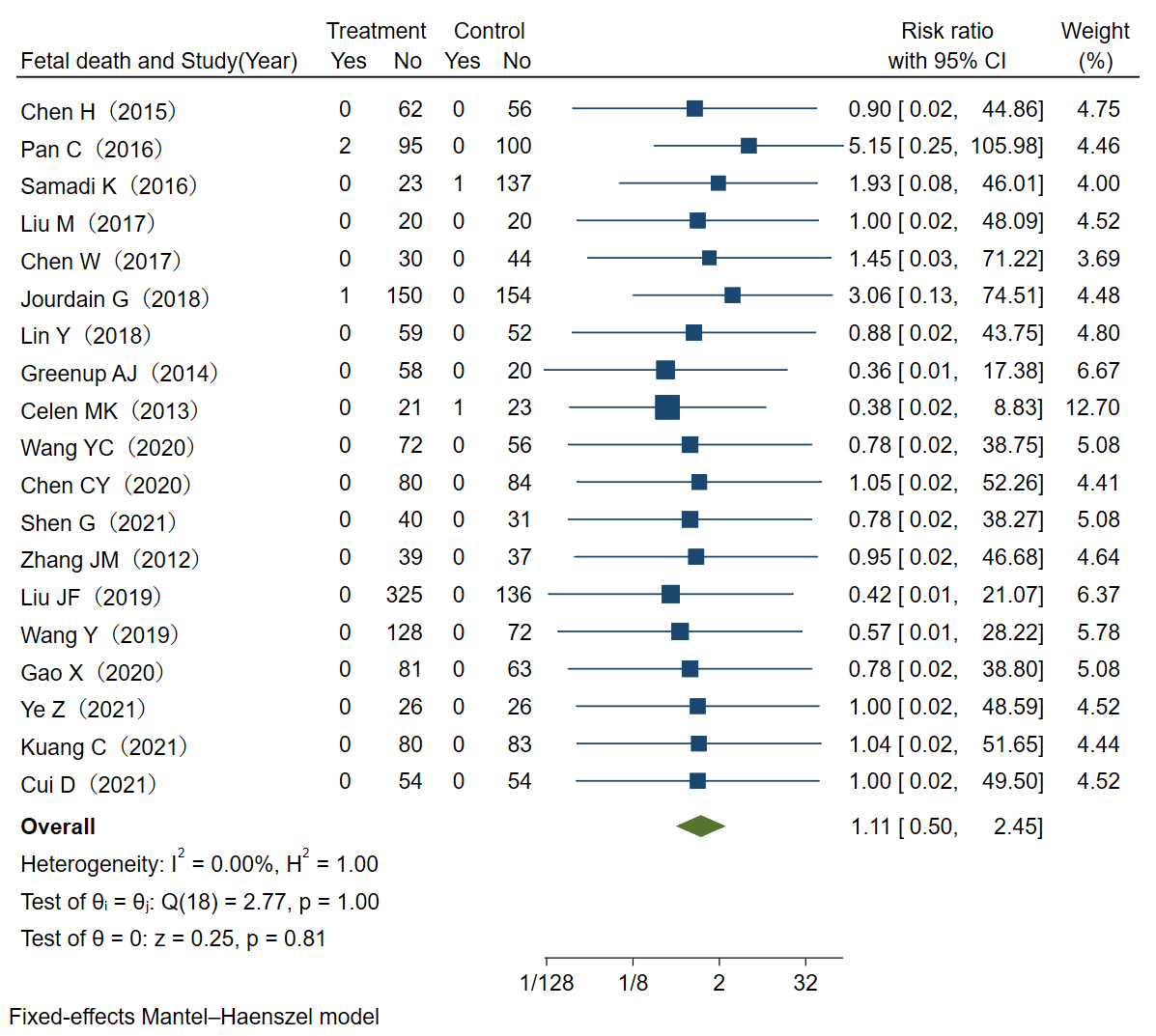


TDF: tenofovir disoproxil fumarate;

95% CI: 95% confidence interval;

Supplementary Figure S4B. Fetal safety of TAF in fetal death


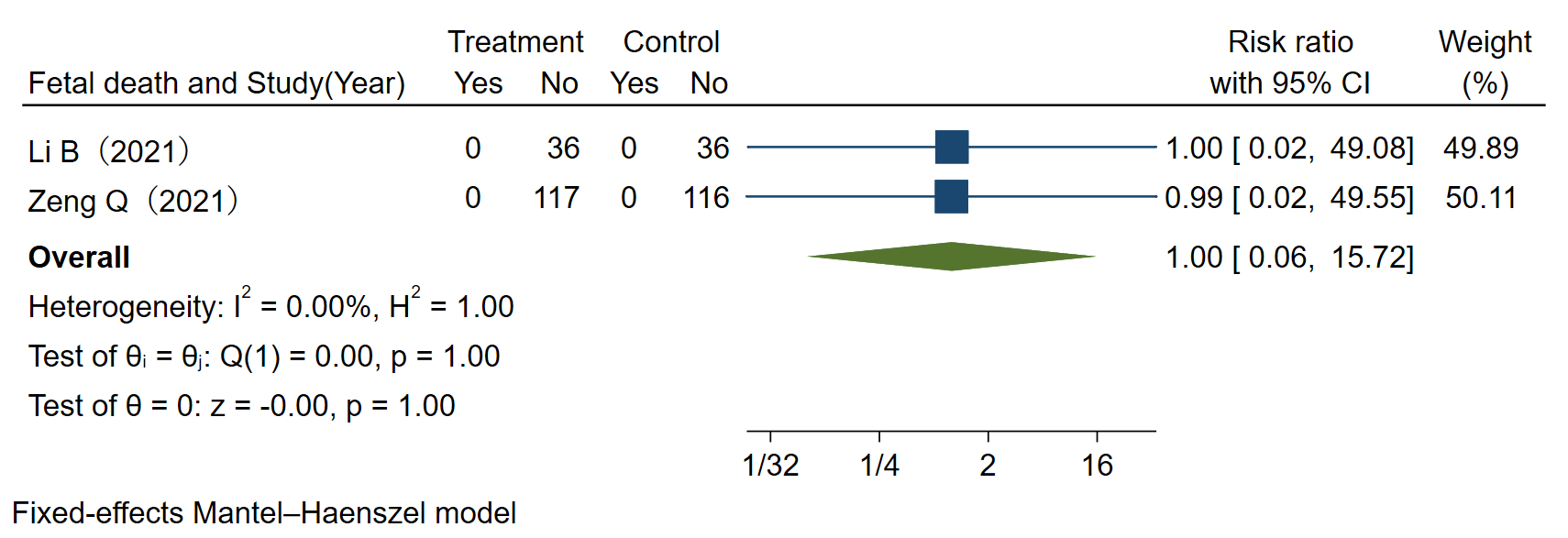


TAF: Tenofovir alafenamide;

95% CI: 95% confidence interval;

Supplementary Figure S4C. Fetal safety of TDF in Apgar score (1 minute)


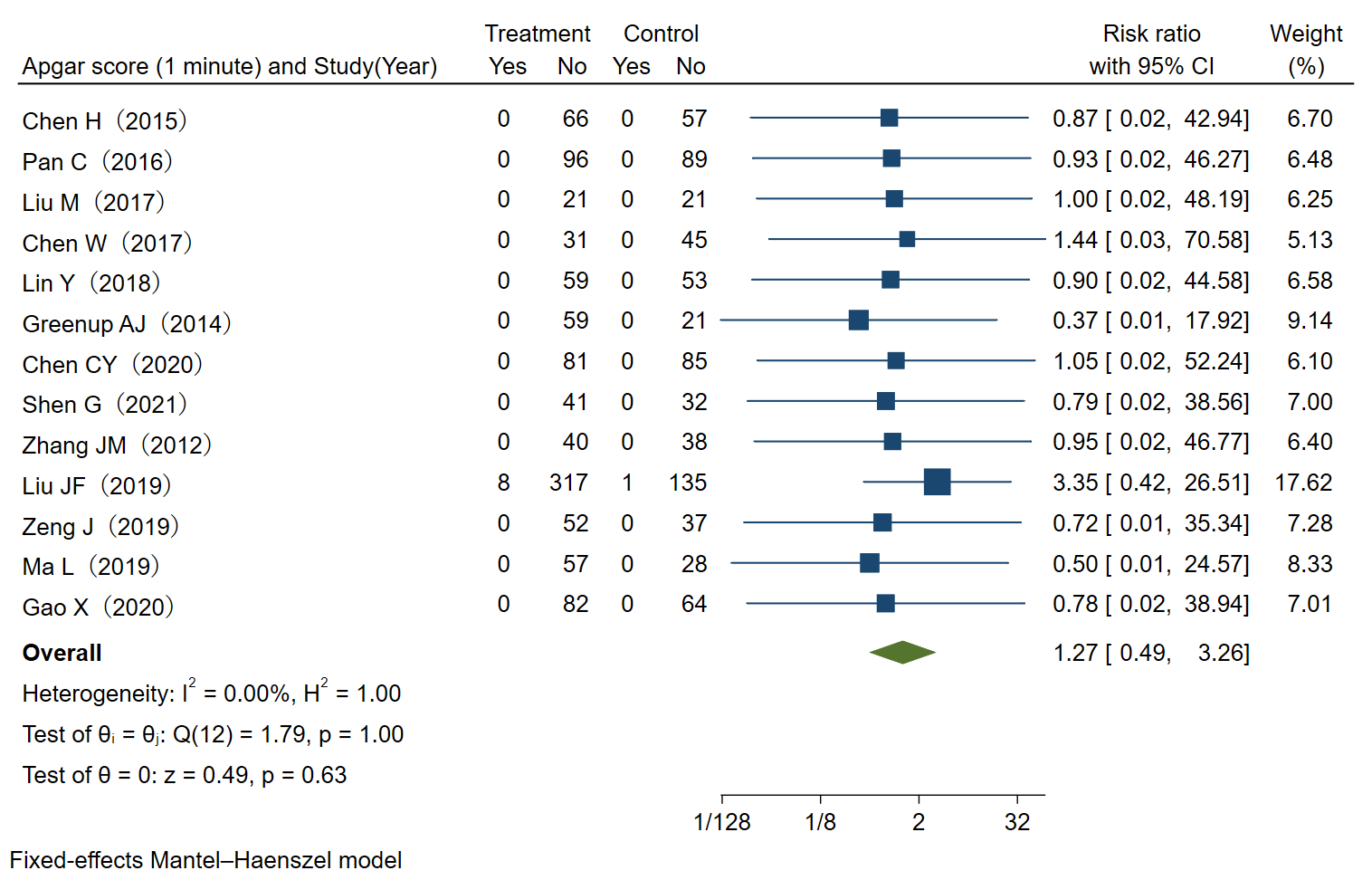


TDF: tenofovir disoproxil fumarate;

95% CI: 95% confidence interval;

Supplementary Figure S4D. Fetal safety of TAF in Apgar score (1 minute)


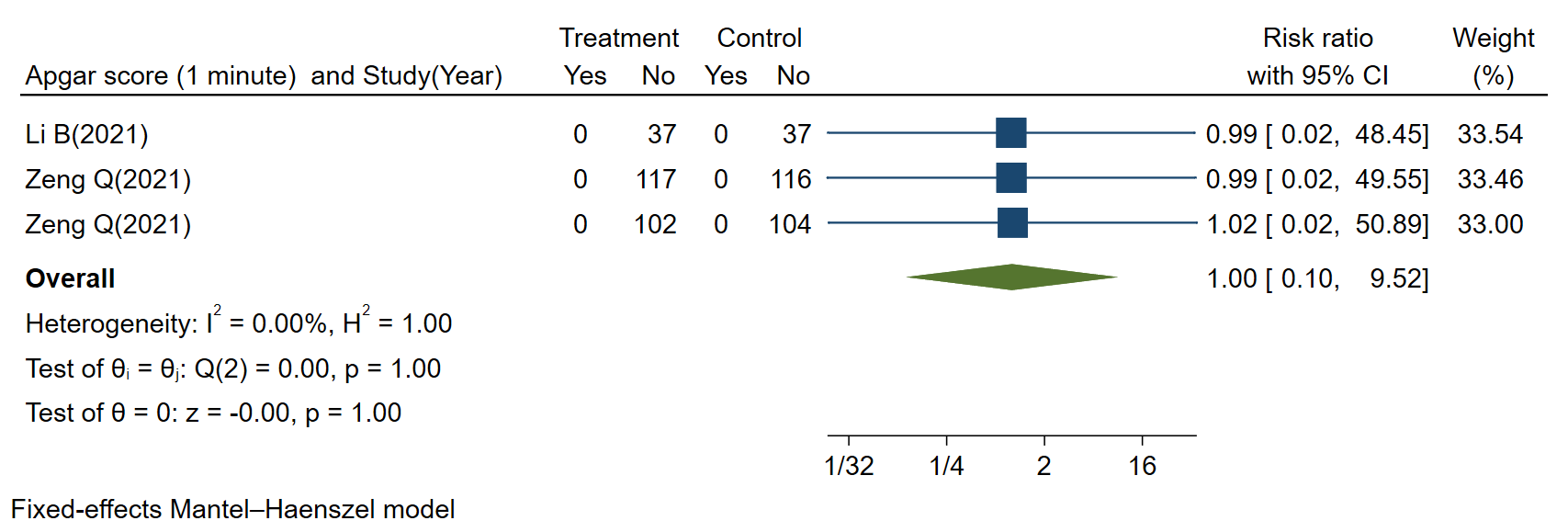


TAF: Tenofovir alafenamide;

95% CI: 95% confidence interval;

Supplementary Figure S4E. Fetal safety of TDF in physical growth


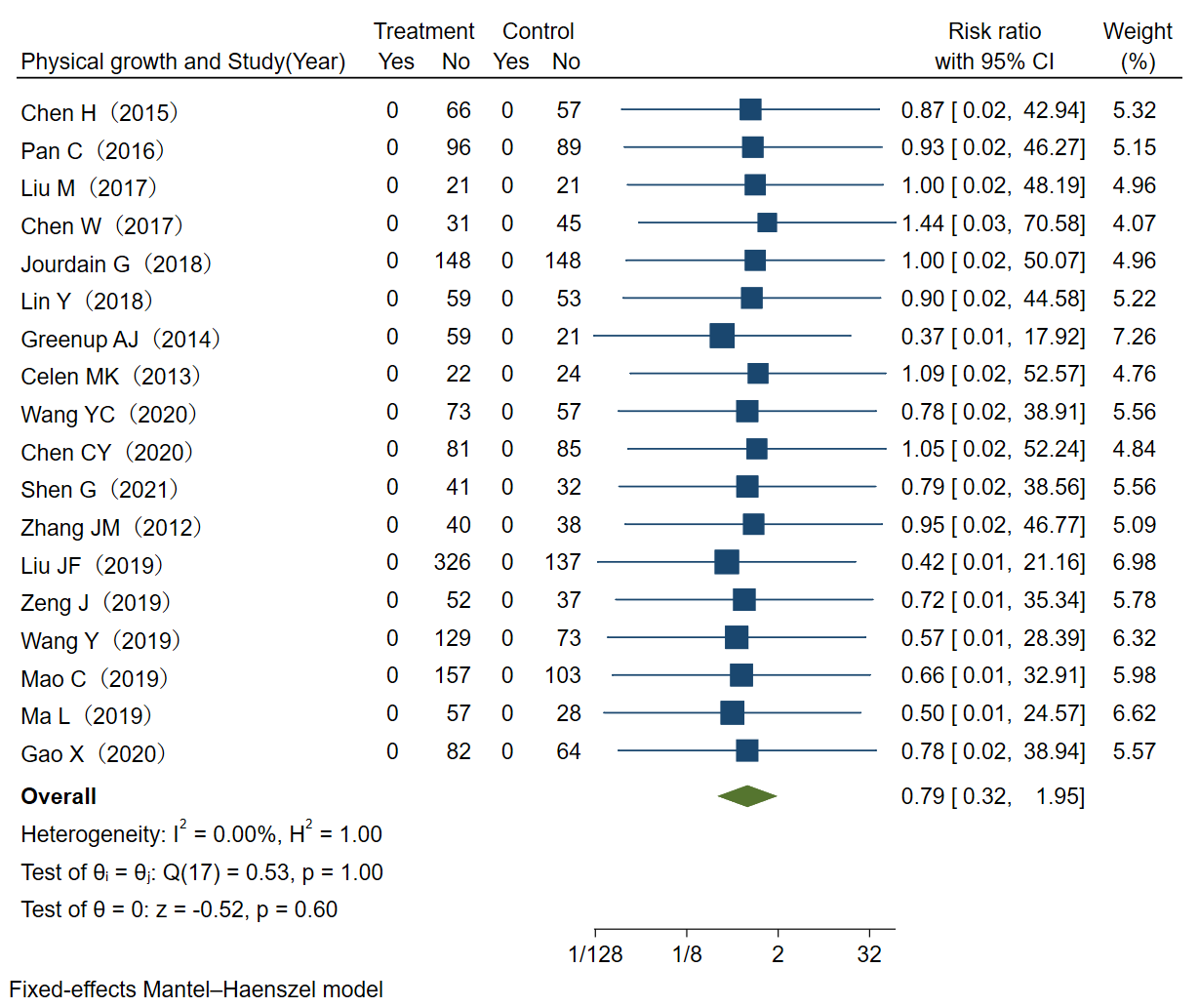


TDF: tenofovir disoproxil fumarate;

95% CI: 95% confidence interval;

Supplementary Figure S4F. Fetal safety of TAF in physical growth


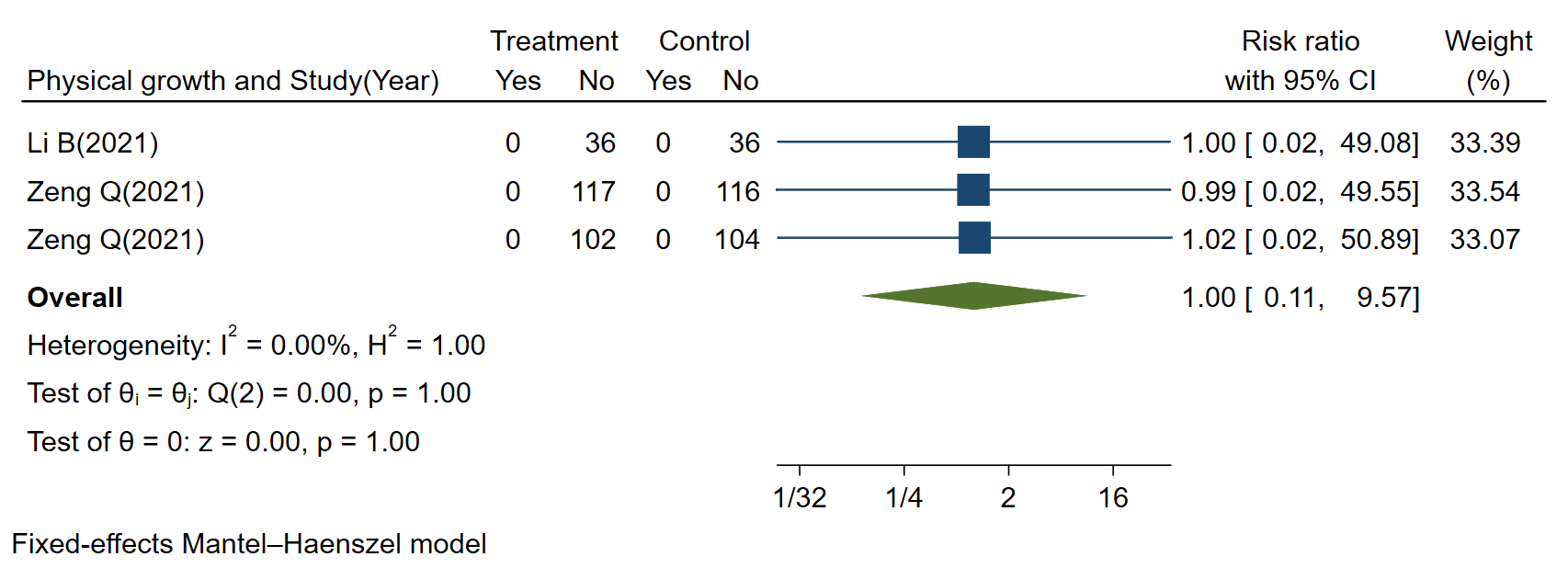


TAF: Tenofovir alafenamide;

95% CI: 95% confidence interval;

Supplementary Figure S4G. Fetal safety of TDF in grade 3 or 4 adverse events^


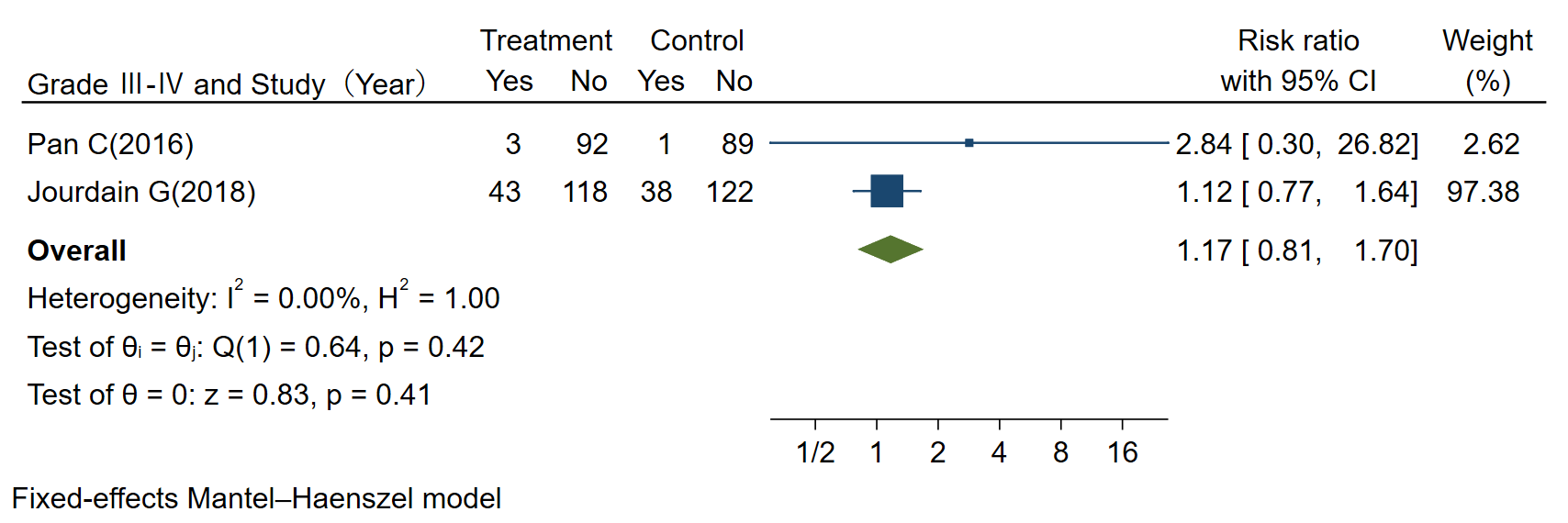


TDF: tenofovir disoproxil fumarate;

Yes/No: events numbers/no events numbers; 95% CI: 95% confidence interval.

^: There were no grade 3 or 4 adverse events reported in TAF studies.

Supplementary Figure S4H. Fetal safety of TDF in bone mineral density scores


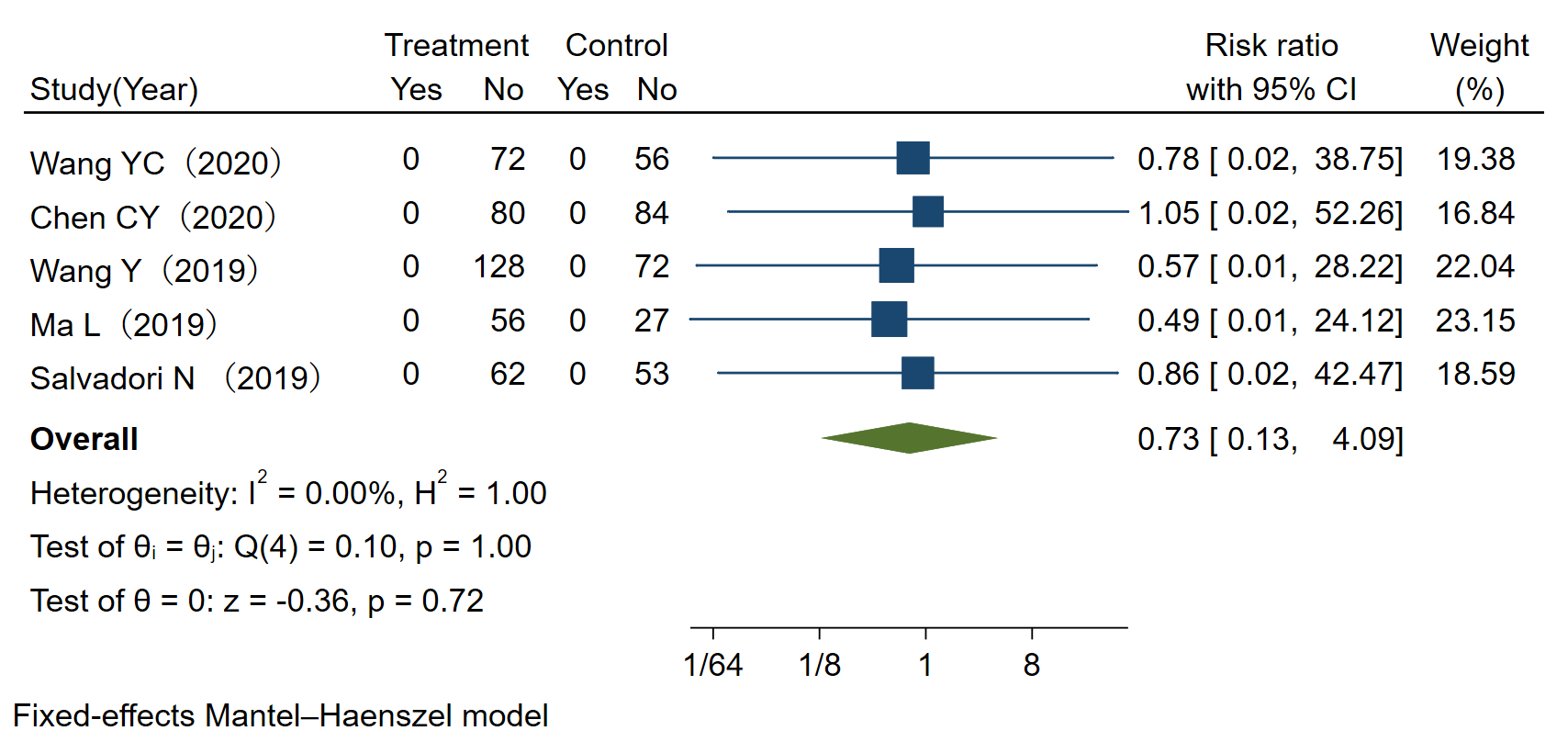


TDF: tenofovir disoproxil fumarate;

95% CI: 95% confidence interval;

Supplementary Figure S4I. Fetal safety of TDF in the physical growth of long-term observations


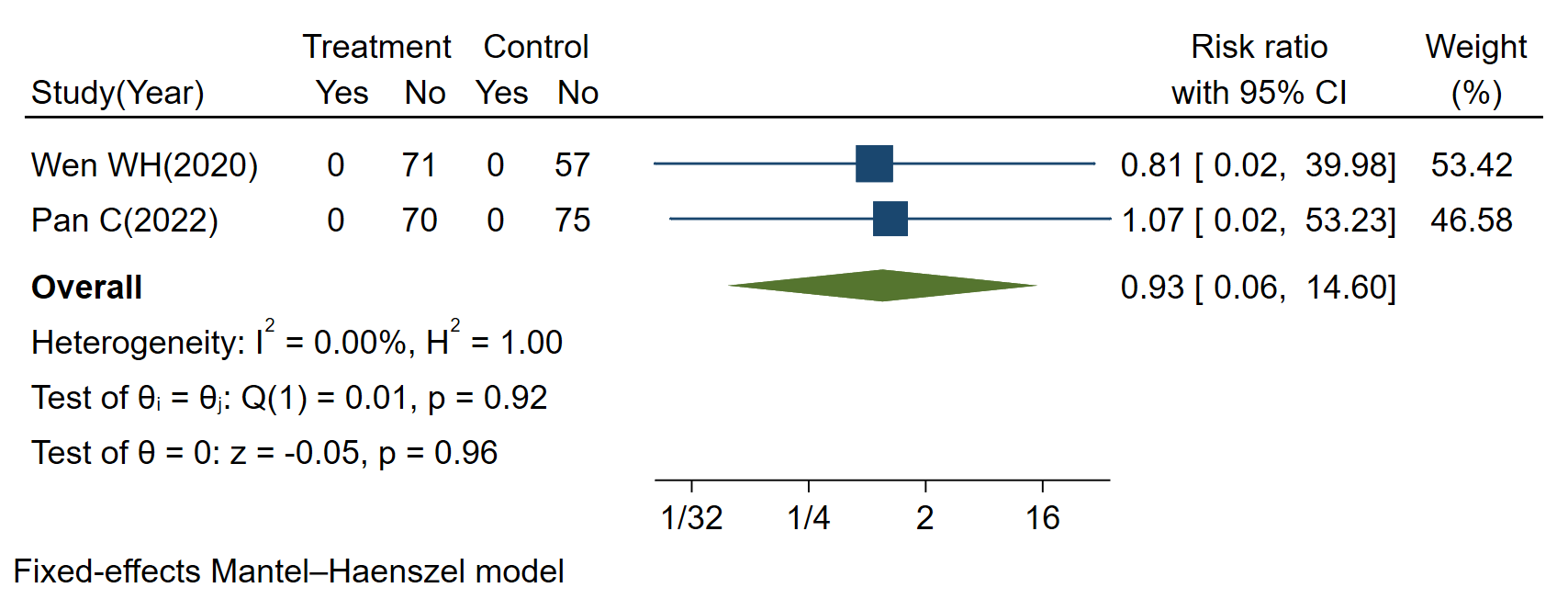


TDF: tenofovir disoproxil fumarate;

95% CI: 95% confidence interval;

Supplementary Figure S4J. Fetal safety of TDF in bone mineral density scores of long-term observations


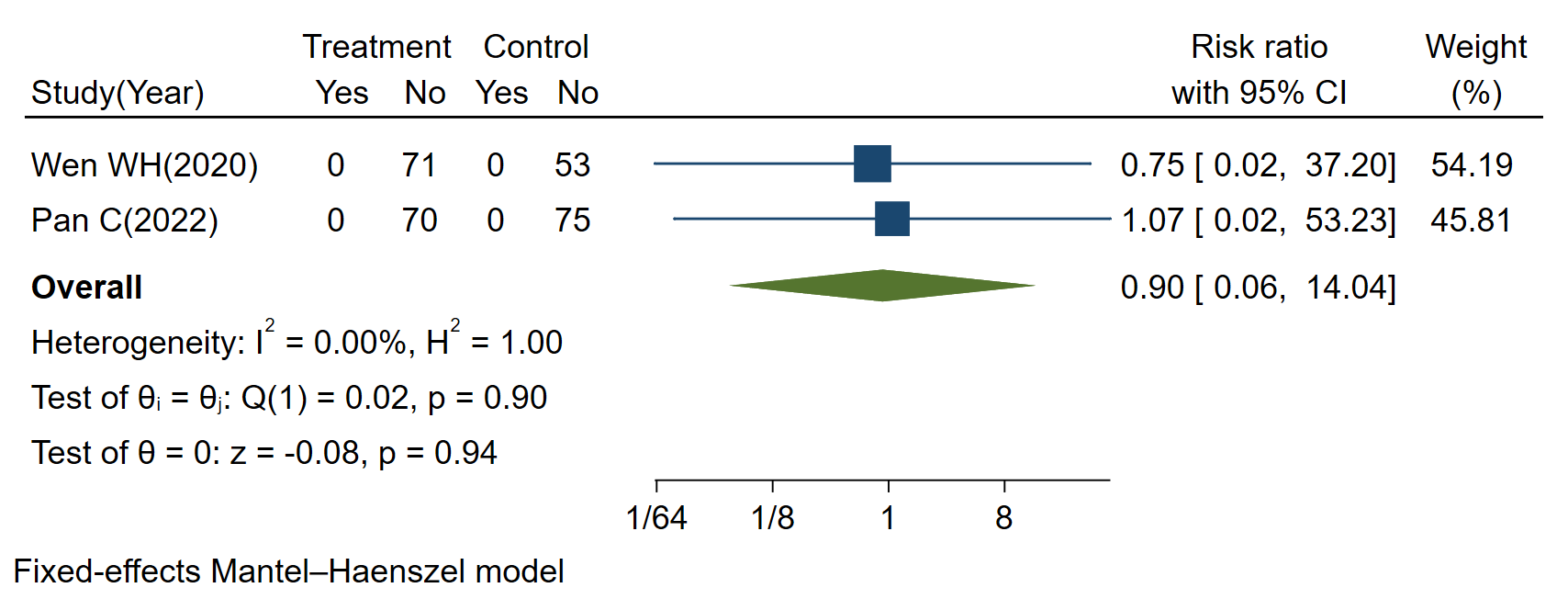


TDF: tenofovir disoproxil fumarate;

95% CI: 95% confidence interval;

**Supplementary Appendix 11.** **Maternal safety of peripartum antiviral prophylaxis using TDF or TAF**

Supplementary Figure S5A. Maternal safety of TDF in pregnancy complications^$^

After using Random-effects models with the DerSimonian-Laird method, The p-value for heterogeneity was 0.38.


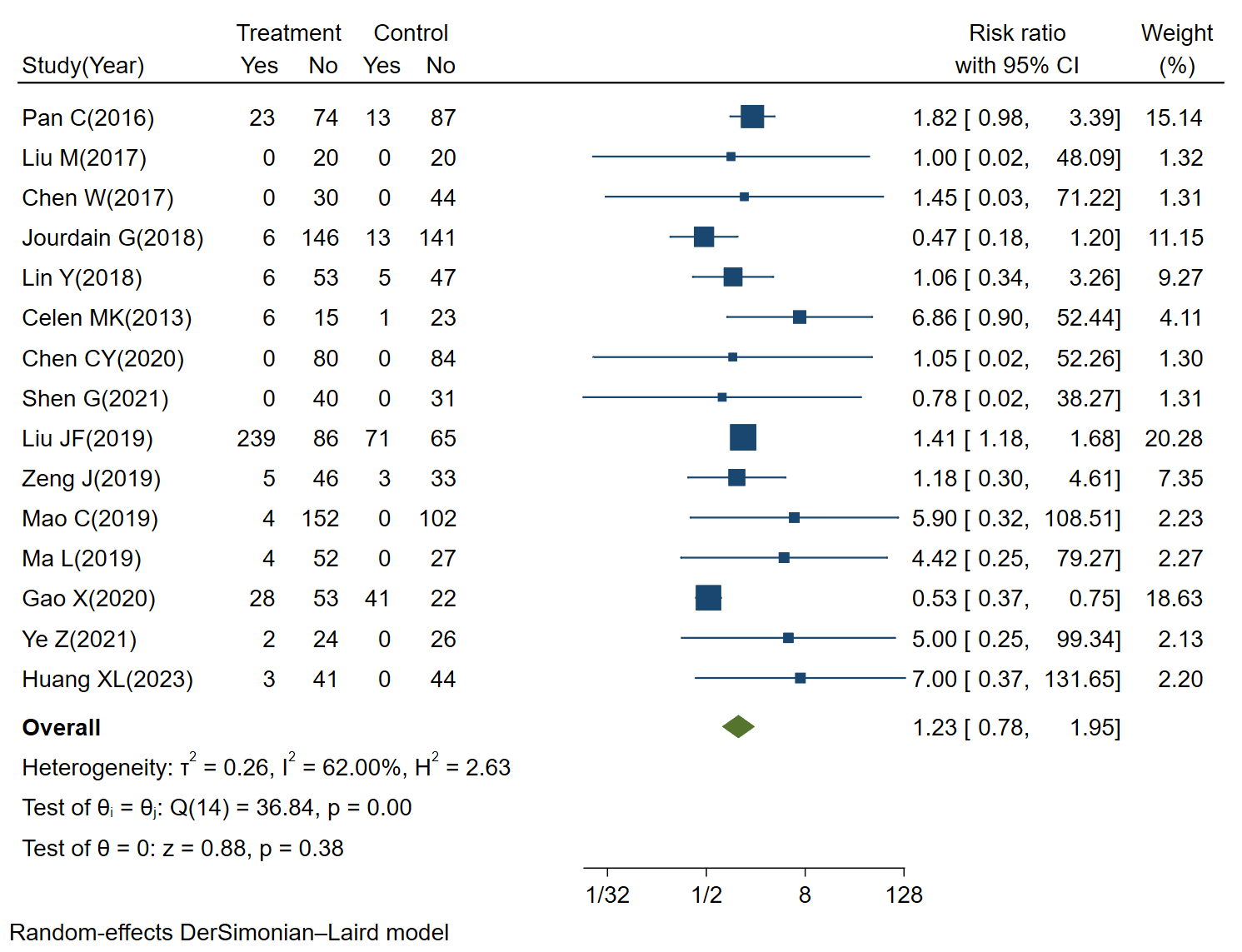


TDF: tenofovir disoproxil fumarate;

Yes/No: events numbers/no events numbers; 95% CI: 95% confidence interval.

^$^: Random-effects models with the DerSimonian-Laird method were used when there was significant heterogeneity (I^2^ value ≥50%)

Supplementary Figure S5B. Maternal safety of TAF in pregnancy complications


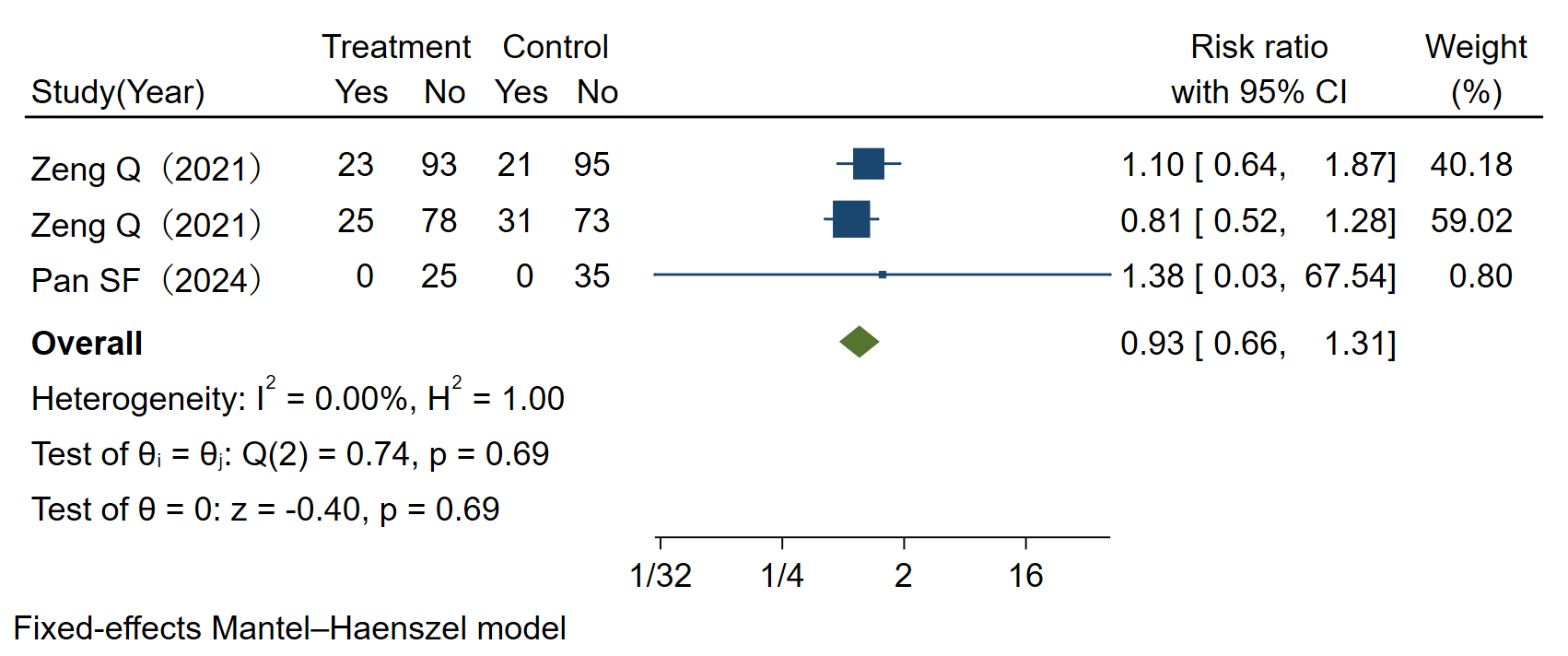


TAF: Tenofovir alafenamide;

Yes/No: events numbers/no events numbers; 95% CI: 95% confidence interval.

Supplementary Figure S5C. Maternal safety of TDF in the elevation of creatine kinase

The p-value = 0.03<0.05


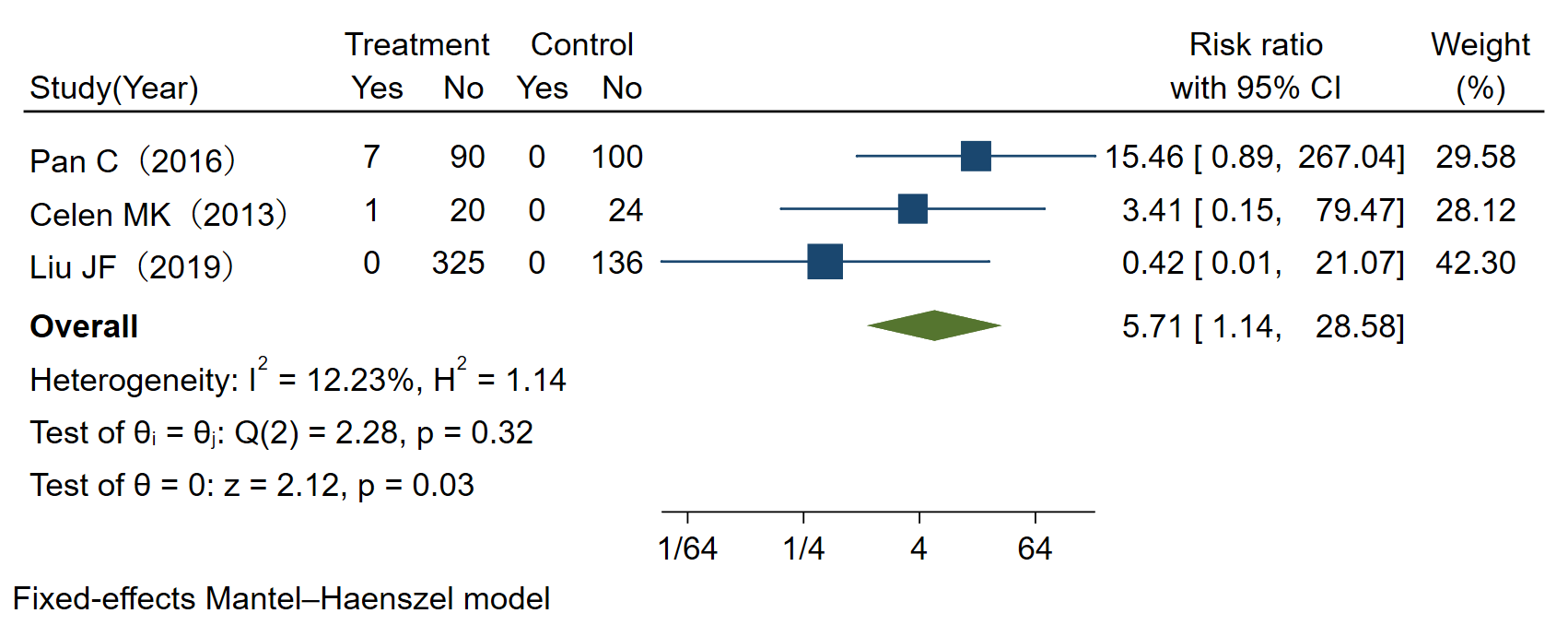


TDF: tenofovir disoproxil fumarate;

Yes/No: events numbers/no events numbers; 95% CI: 95% confidence interval.

Supplementary Figure S5D. Maternal safety of TDF in postpartum hemorrhage


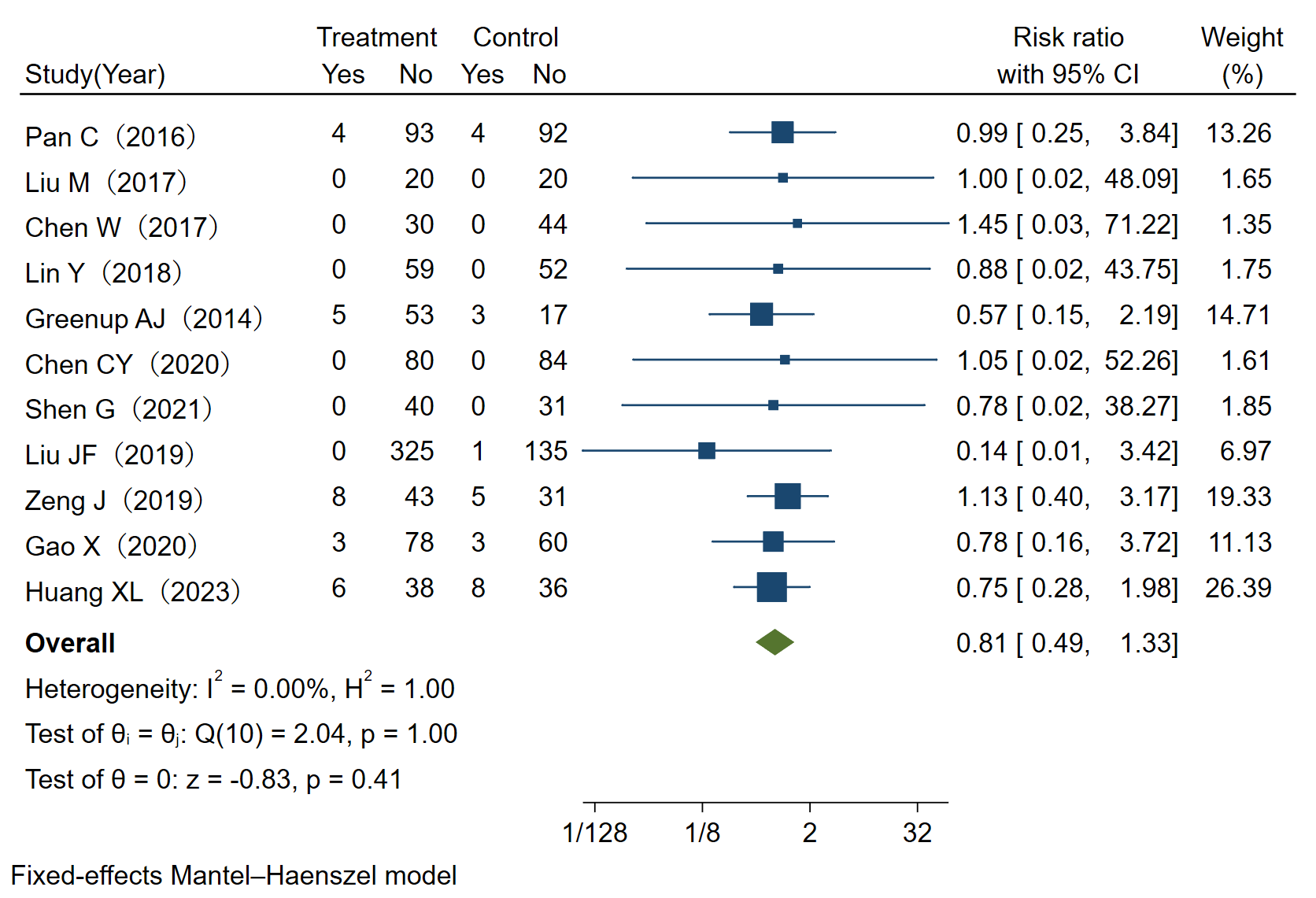


TDF: tenofovir disoproxil fumarate;

Yes/No: events numbers/no events numbers; 95% CI: 95% confidence interval.

Supplementary Figure S5E. Maternal safety of TAF in postpartum hemorrhage


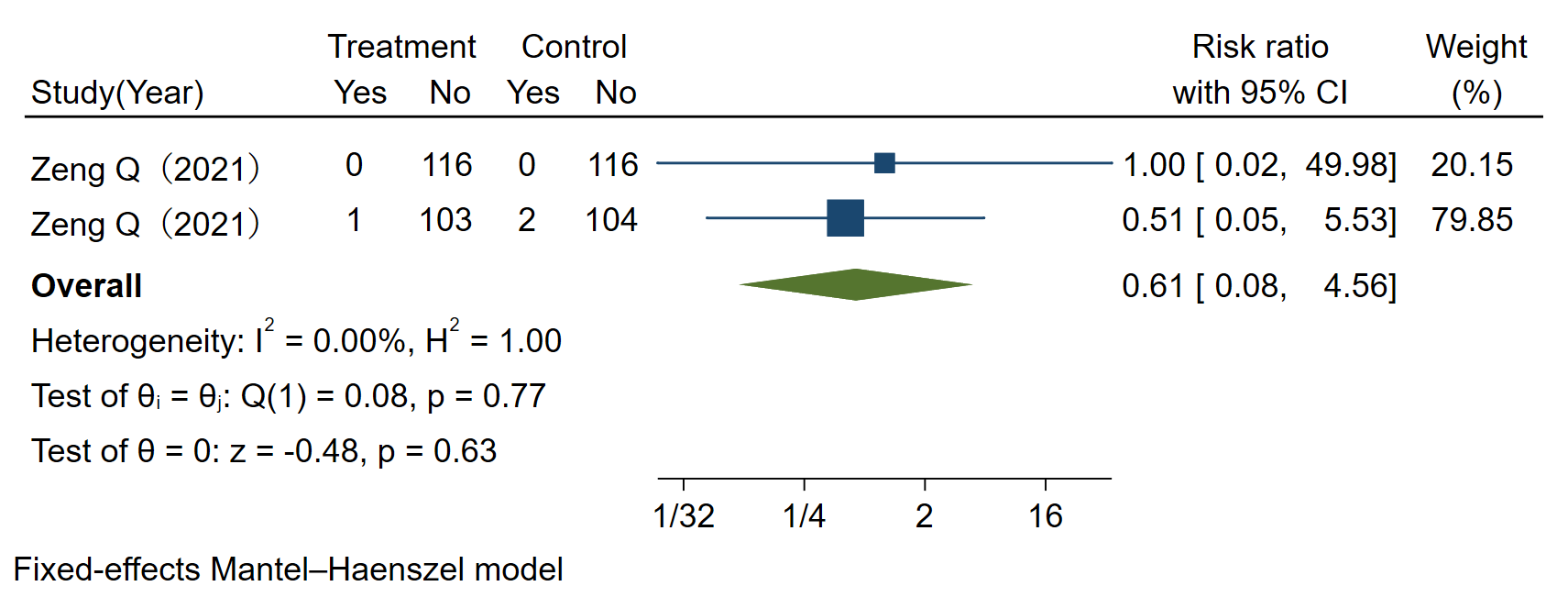


TAF: Tenofovir alafenamide;

95% CI: 95% confidence interval;

Supplementary Figure S5F. Maternal safety of TDF in severe adverse events (grades III and IV)^


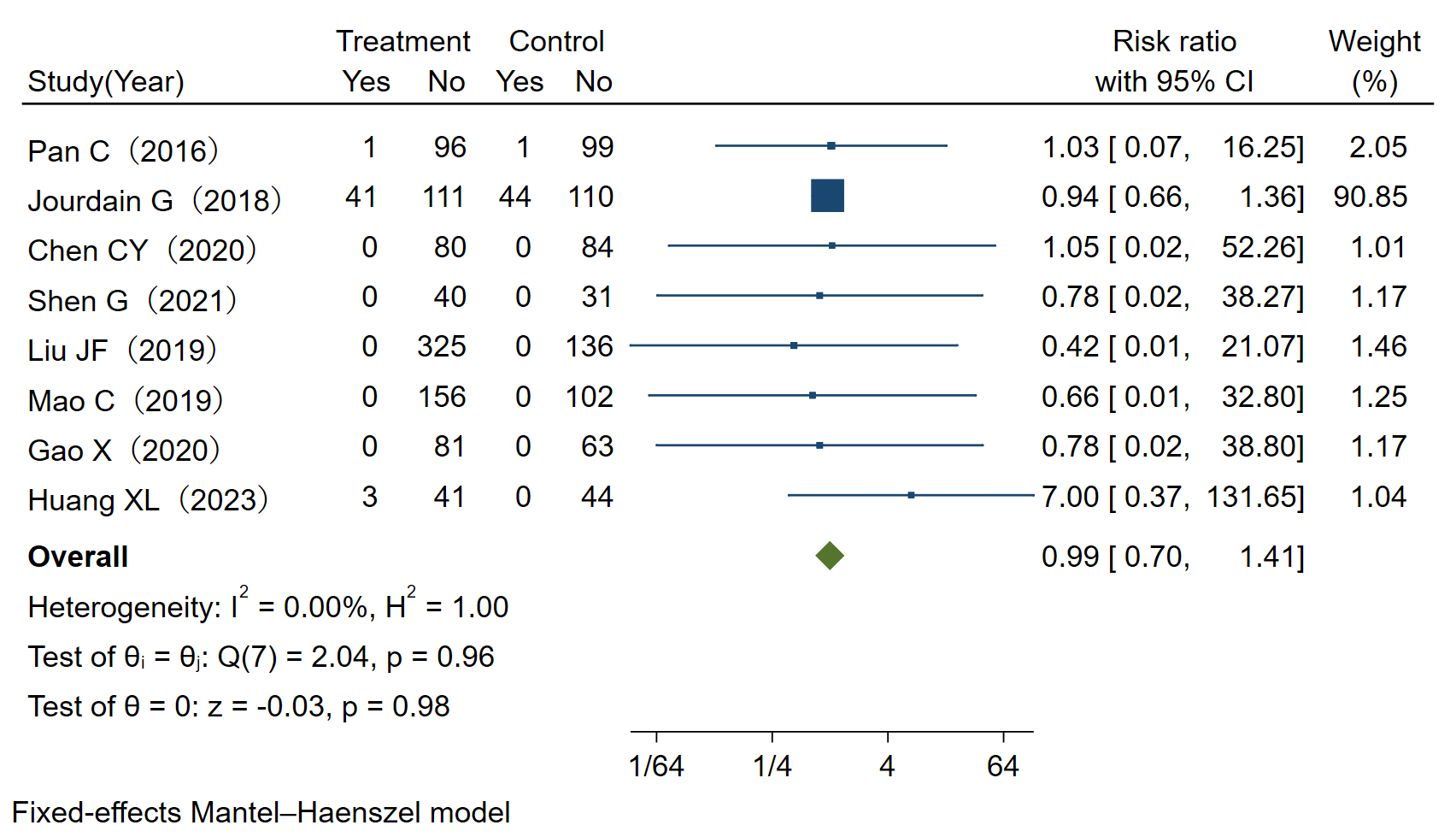


TDF: tenofovir disoproxil fumarate;

Yes/No: events numbers/no events numbers; 95% CI: 95% confidence interval.

^ There were no grade 3 or 4 adverse events reported in TAF studies.

**Supplementary Appendix 12. Publication bias**

Supplementary Figure S6A. Publication Bias Assessment (Funnel Plots) of the primary outcome in TDF studies


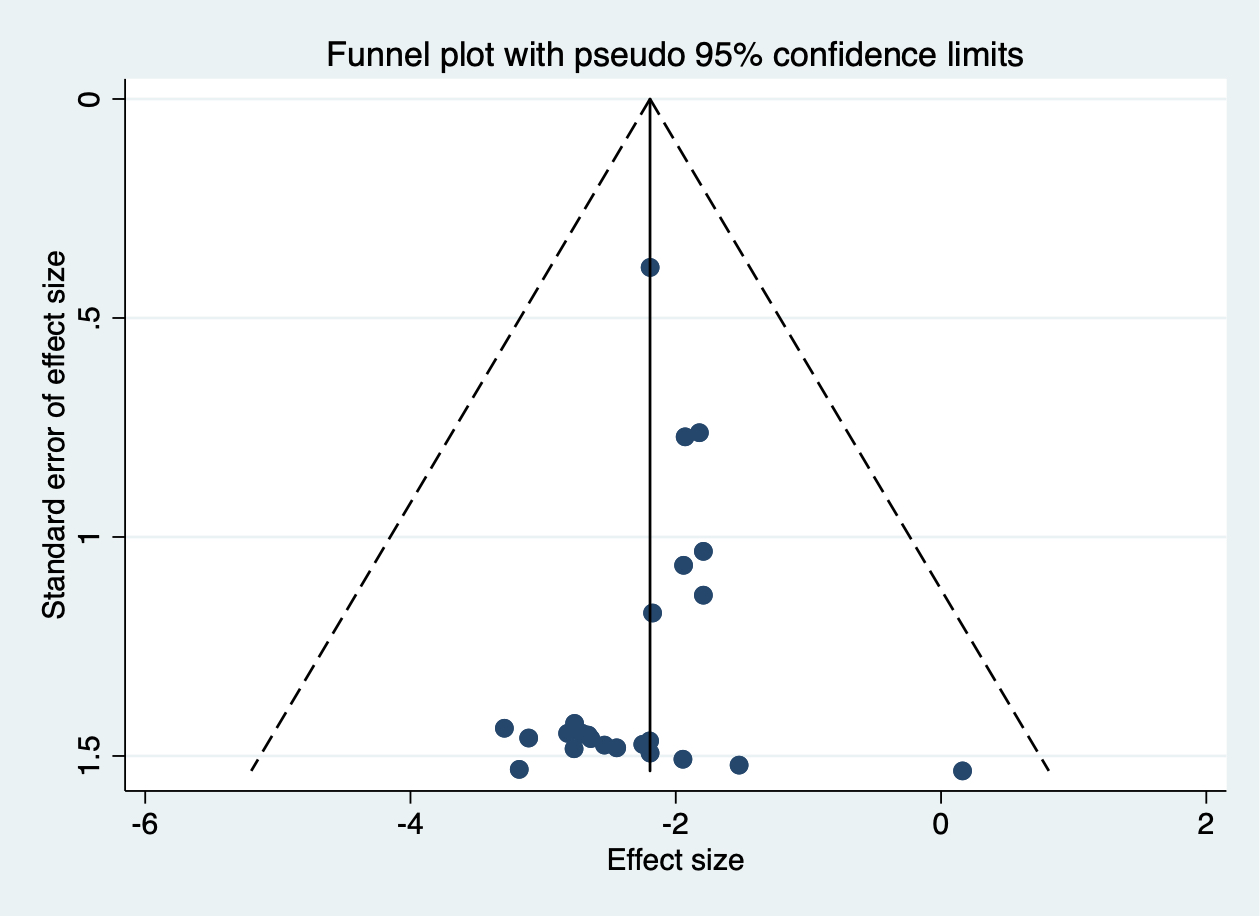


Supplementary Figure S6B. Publication Bias Assessment (Egger’s test) of the primary outcome in TDF studies


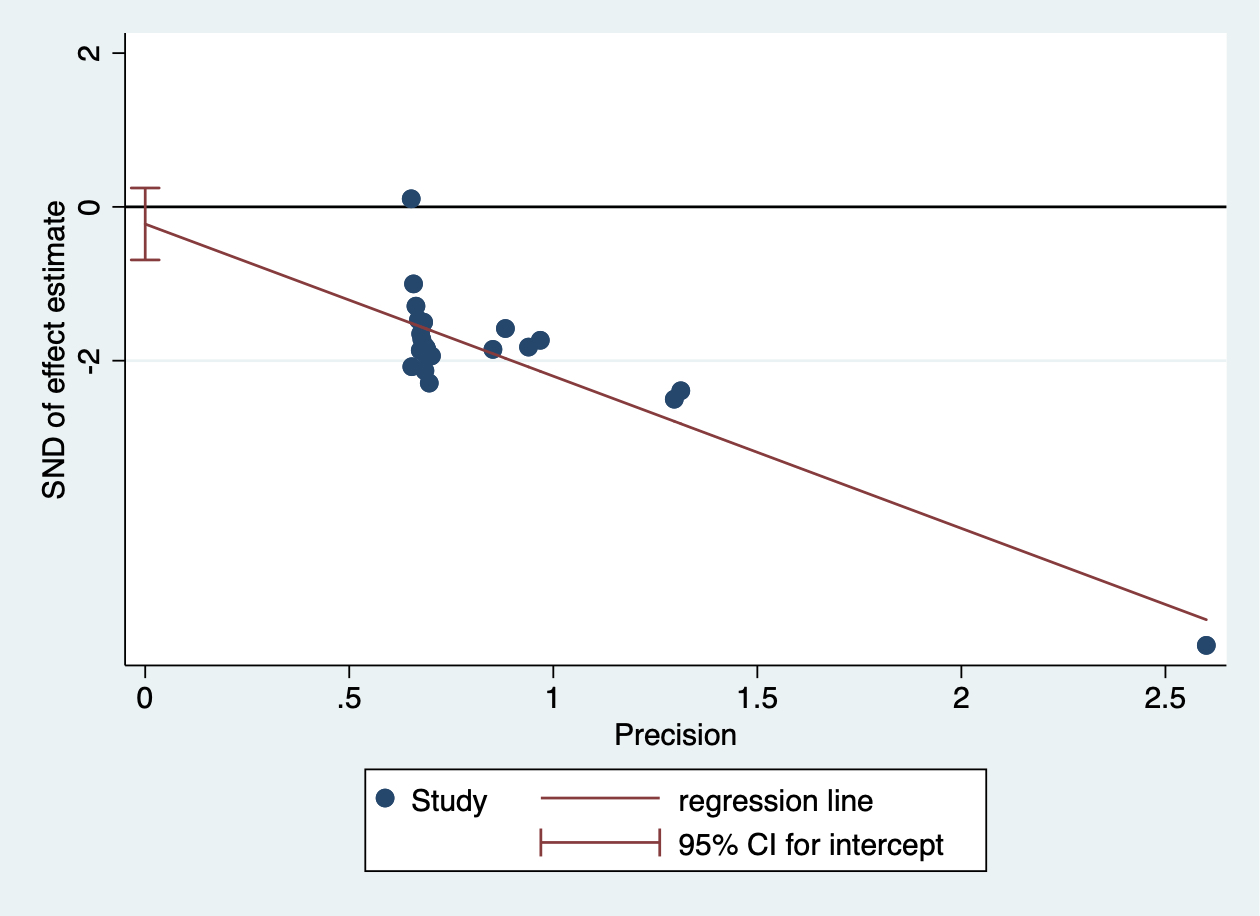


Egger’s test: p-value = 0.34.

Supplementary Figure S6C. Publication Bias Assessment (Funnel Plots) of the primary outcome in TAF studies


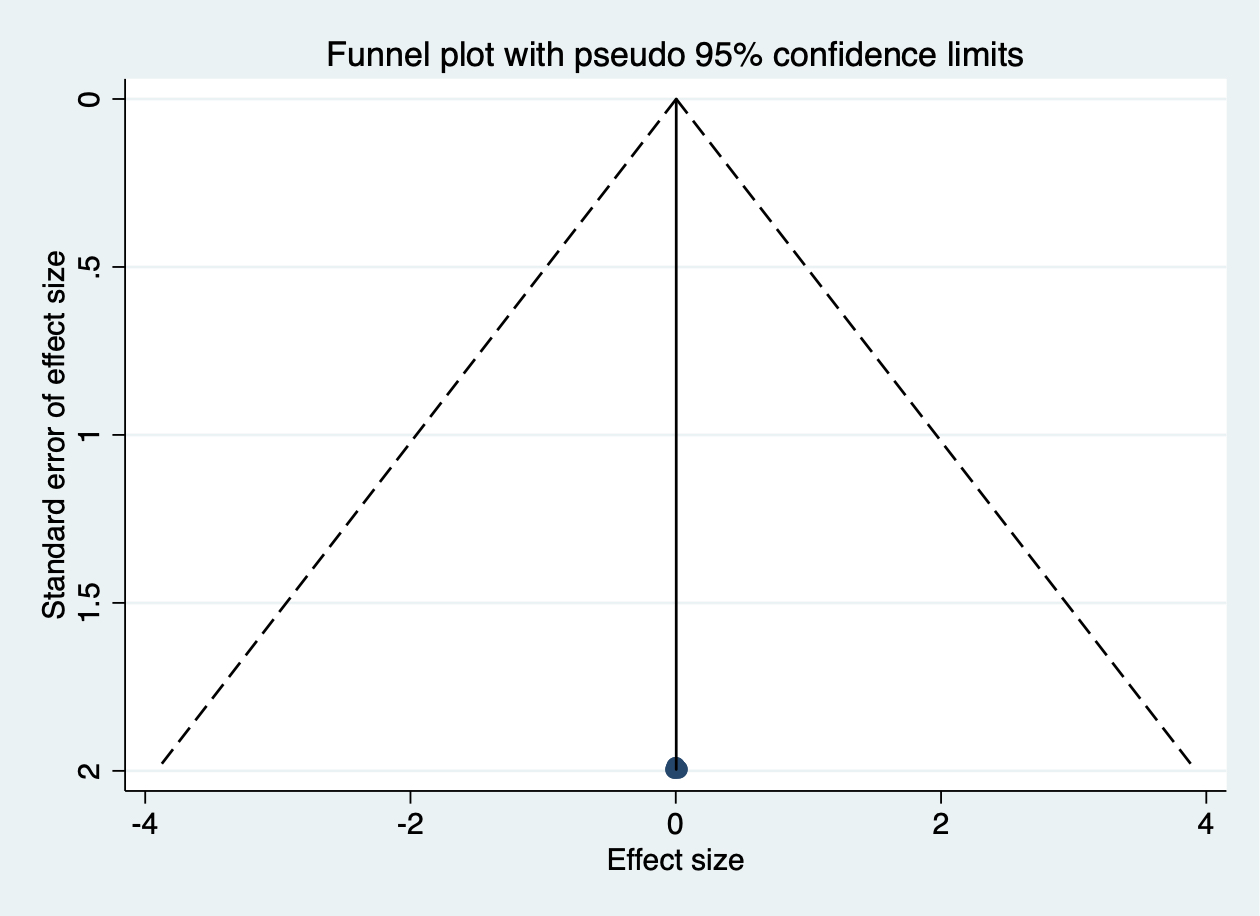


Supplementary Figure S6D. Publication Bias Assessment (Egger’s test) of the primary outcome in TAF studies


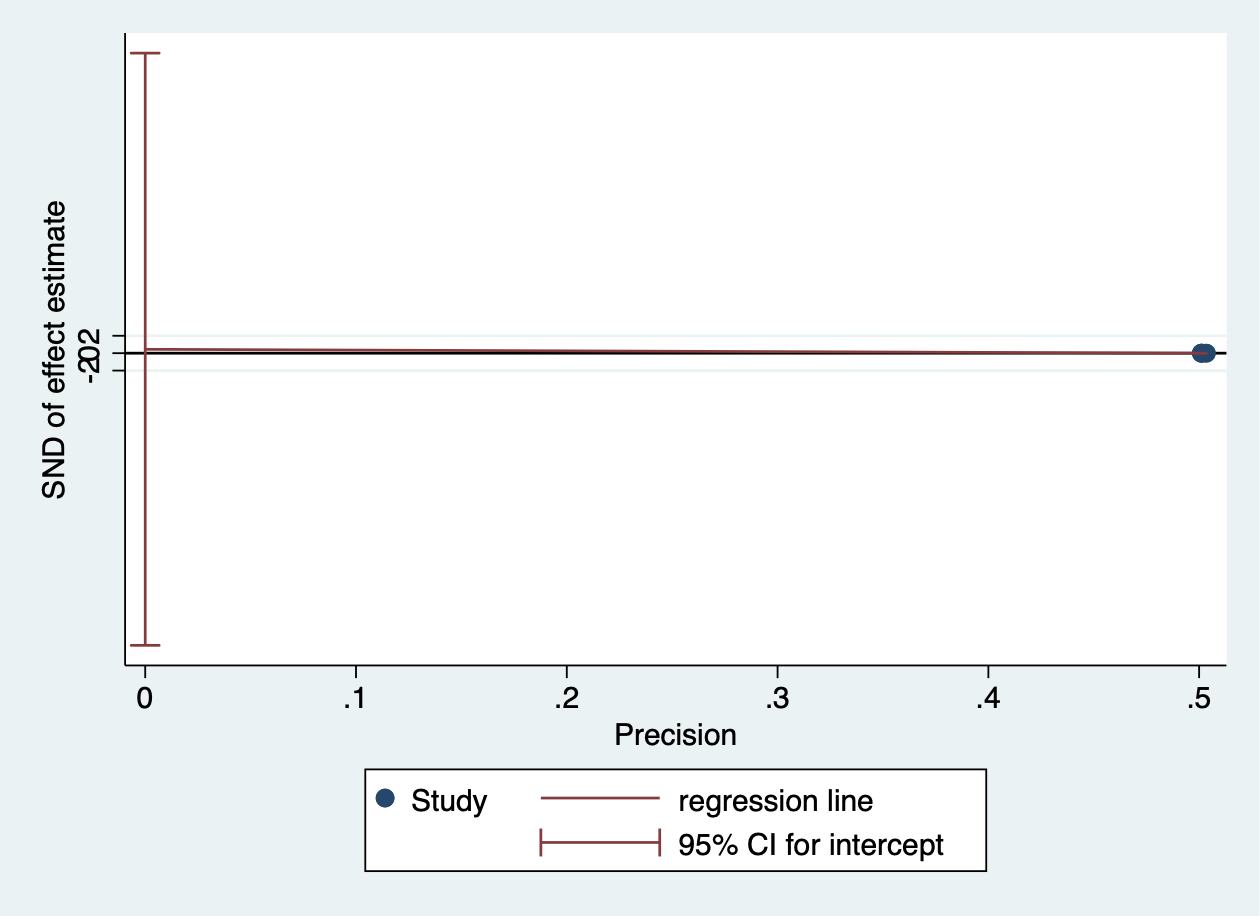


Egger’s test: p-value = 0.89.
